# CBX3 confers ferroptosis resistance during blood-borne metastasis

**DOI:** 10.1186/s13045-025-01777-0

**Published:** 2026-01-15

**Authors:** Chun Wu, Xuefei Liu, Boxi Zhao, Mao Zhao, Binyu Zhang, Guanyin Huang, Yixin Cheng, Shuqian Zheng, Jianyang Hu, Ling Guo, Weinan Guo, Jun Tan, Xin Hong

**Affiliations:** 1 Department of Biochemistry School of Medicine, SUSTech Homeostatic Medicine Institute Southern University of Science and Technology, Shenzhen, 518055 Guangdong China; 2 Department of Nasopharyngeal Carcinoma, State Key Laboratory of Oncology in South China, Sun Yat-sen University Cancer Center, Guangzhou, China; 3 Department of Hematology and Oncology, Shenzhen Children’s Hospital and School of Medicine, Southern University of Science and Technology, Shenzhen, China; 4 Department of Dermatology, Xijing Hospital Fourth Military Medical University, Xi’an, China; 5 Department of Physiology & Pathophysiology, School of Basic Medical Sciences, Capital Medical University, Beijing, 100069 China; 6 Department of Neurosurgery, Xiangya Hospital Central South University, Changsha, Hunan China; 7 National Clinical Research Center for Geriatric Disorders, Xiangya Hospital, Central South University, Changsha, Hunan China; 8 Key University Laboratory of Metabolism and Health of Guangdong, Southern University of Science and Technology, Shenzhen, Guangdong China; 9 Guangdong Provincial Key Laboratory of Cell Microenvironment and Disease Research, Southern University of Science and Technology, Shenzhen, Guangdong China

**Keywords:** CTCs, Ferroptosis resistance, CBX3, GPX4, Clinical correlation

## Abstract

The survival mechanisms of circulating tumor cells (CTCs) remain poorly understood while these rare cell populations transit through the blood stream and colonize distant organs including the brain. Using single-cell RNA-seq of microfluidically-enriched CTCs and patient-matched brain metastatic tumor cells of lung adenocarcinoma (LUAD), we revealed CTC-selective upregulation of core gene signatures associated with ferroptosis. The malignancy of CTCs was confirmed through comparative genomic instability and copy number variation (CNV) analyses of paired CTCs and metastatic tumor cells using both transcriptomic and whole-exome sequencing. Among the transcription factors elevated in CTCs, Chromobox 3 (CBX3) was the top hit, which was tightly correlated with GPX4 expression. Functionally, CBX3 co-operated with EP300 to protect CTCs from ferroptosis by upregulating GPX4 expression. Genetic depletion of CBX3 triggered ferroptosis and substantially reduced tumor cell survival and invasiveness in two independent cancer cell models. Conversely, CBX3 overexpression was sufficient to promote tumor growth, migration, and invasion in vitro, and metastatic progression in vivo. Clinically, CBX3 expression was significantly correlated with TNM stage and was predictive of both progression-free interval and overall survival in LUAD. In a prospective cohort of LUAD and melanoma patients, CTCs co-expressing CBX3 and GPX4 were selectively elevated in the blood samples of metastatic cases compared to the non-metastatic group, highlighting the clinical association between ferroptosis-resistant CTCs and metastatic progression.

**Supplementary Information:**

The online version contains supplementary material available at 10.1186/s13045-025-01777-0.

**To the editor**,

Brain metastases (BrM) occur when CTCs colonize the brain, a lethal process that frequently found in lung cancer and melanoma [1, 2]. Ferroptosis, a novel form of cell death driven by lipid peroxidation, is critically involved in cancer metastasis [3]. Chromobox 3 (CBX3), a member of the heterochromatin protein 1 (HP1) family, regulates epigenetics and transcription of cancer-associated genes [4]. Here, we integrated microfluidic isolation of patient CTCs with single-cell omics and uncovered that CBX3 co-operated with EP300 to protect CTCs from ferroptosis by modulating GPX4 expression.

We performed single-cell omic profiling of CTCs and BrM tumors from four LUAD patients (Fig. 1A-B and S1A, Table S1). CTCs (~ 0.1%−10% purity) were enriched using a size-based microfluidic separation platform (Figure S1B) [5], and subjected to scRNA-seq with BrM tumors, generating 42,215 single cells across 16 major types (Fig. 1C-D). A distinct cluster of 1153 CTCs expressed ZEB2, NRG1, and the epithelial markers (*KRT8*, *KRT18* and *EPCAM*) at low levels (Fig. 1D-E, Table S2−3) [6]. Antibody-derived tags (ADT) technology [7] was employed to simultaneously analyze EPCAM protein and mRNA in one patient (H41). We confirmed that CTCs specifically upregulated EPCAM expression, but not in contaminating immune cells (Fig. 1F-G and S1C).

Genomic instability is an intrinsic feature of malignant cells that distinguishes them from normal cells [8]. Genomic instability and inferCNV analyses revealed the highest genomic instability in tumors and CTCs as validated by whole-exome sequencing (WES) (Fig. 1H-J, S1D). At least one patient-matched somatic mutation was found in 5.46% (63/1153) of CTCs using scRNA-seq analysis (Fig. 1K and S1E). Consistently, a significant downregulation of epithelial marker mRNAs in CTCs was observed in HCC (Figure S1F). Thus, 1153 potential true CTCs were validated in four LUAD patients with BrM.

**Fig. 1:**
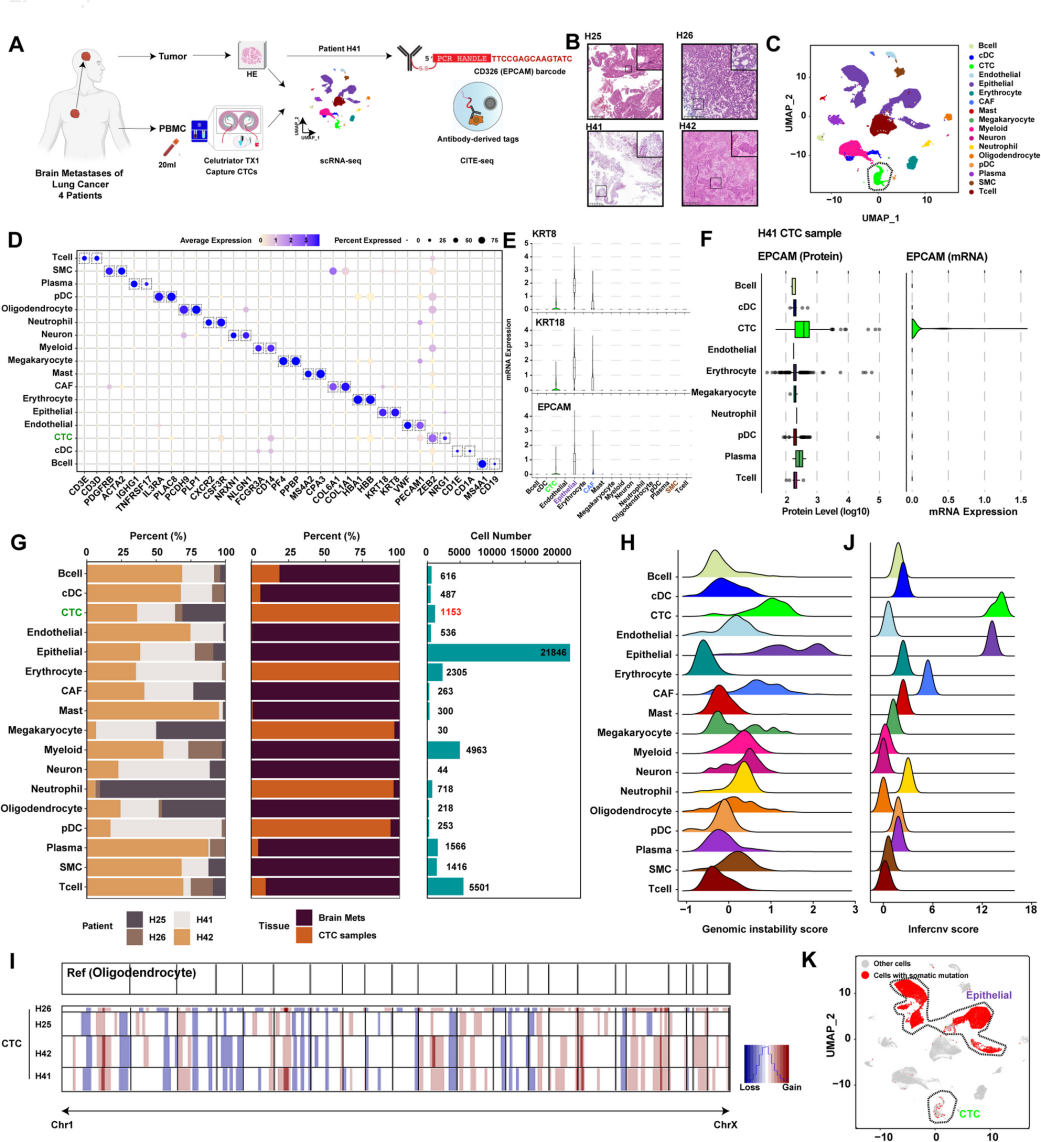
Single-cell omic characterization of CTCs from LUAD patients with BrM.**A.** Schematic illustration of sample collection, processing, and single-cell analysis.**B.**H&E-stained tissue sections showing the morphology of lung cancer brain metastasis.**C.** UMAP plot showing the subtypes of all cells, each dot indicated a single cell. Color-coded for the cell type.**D.** Dot plot showing the selected markers for each subtype of all cells. Dot size indicates the fraction of expressing cells and the colors represent normalized gene expression levels.**E**. Violin plot showing the mRNA expression level of *KRT8*, *KRT18 *and *EPCAM* for each subtype of all cells.**F.** Boxplot showing EPCAM protein levels and mRNA levels across different subtypes of CTCs sample isolated from the whole blood of patient H41.**G.** Bar plot showing the proportion of the different cell subtypes across patients (left) and tissues (middle). Bar plot showing the cell number of the different cell subtypes (right).**H.** Ridgeline plot showing the genomic instability score of all cell subtypes. Cells with a genomic instability score >1 (dashed line) are classified as possessing high chromosomal instability, indicative of malignancy.**I.** Heatmap showing large-scale CNVs for CTCs from four patients, inferring from single-cell RNA-seq analysis. Oligodendrocytes as reference cells. Colors indicate the CNV states. Red: amplifications (Gain); blue: deletions (Loss).**J. **Ridgeline plot showing inferCNV score of all cell subtypes. The calculation of InferCNV scores is based on Figure 1I: a score of 1 is added for each chromosome harboring CNV, with the final score determined by the copy number alteration status across all chromosomes.**K.** UMAP showing all cells from Figure 1C. Red highlights CTCs with at least one somatic mutation identified through WES

**Fig. 2:**
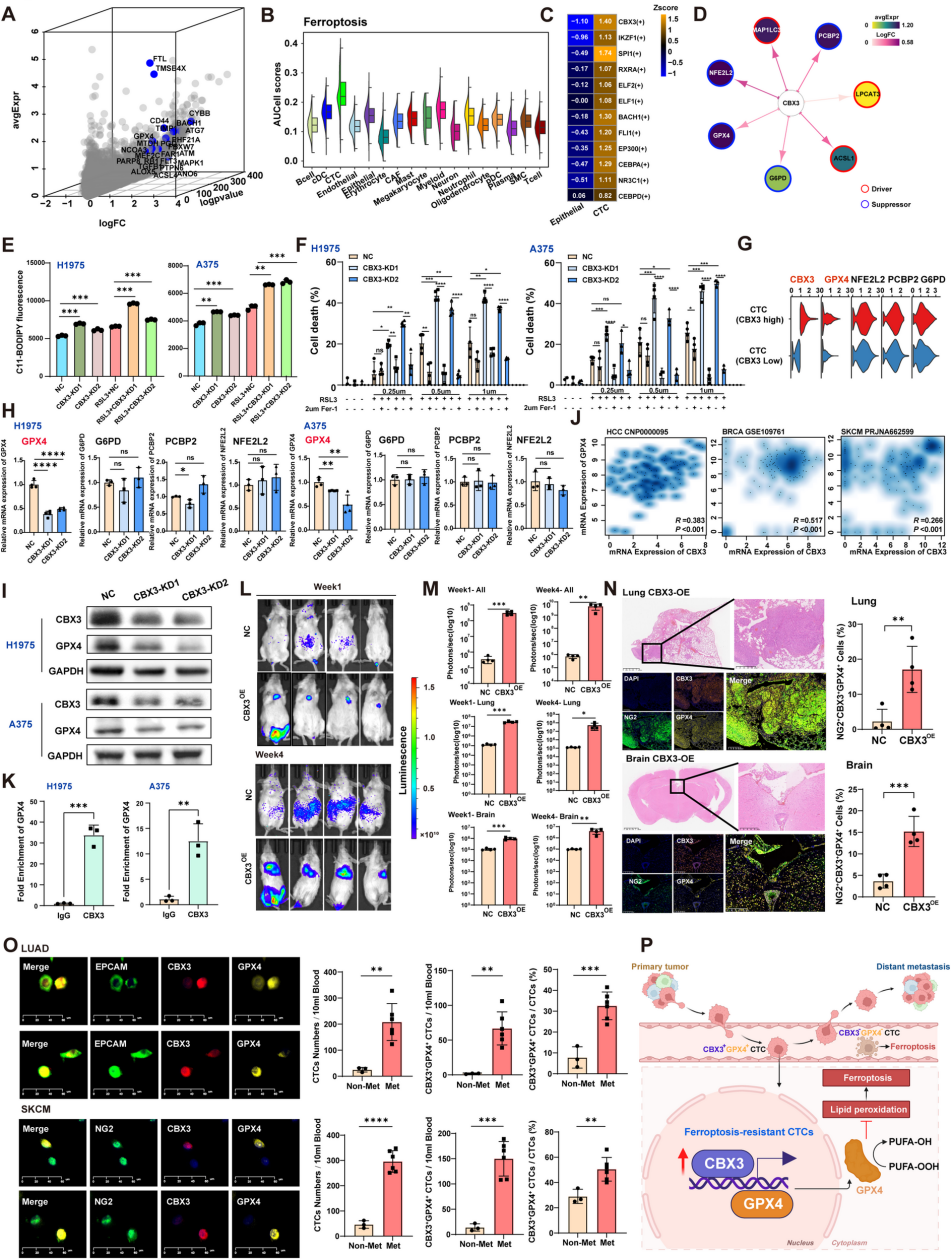
Experimental and clinical validations of CBX3 in ferroptosis resistance and CTC-mediated metastatic progression.**A.** Three-dimensional plot showing genes with elevated expression in CTCs. Blue-highlighted genes are ferroptosis pathway components.**B.** Box plot showing the ferroptosis scores among the all-cell subtypes. Ferroptosis scores were calculated using AUCell.**C. **Heatmap showing the activity of regulatory TFs in CTCs calculated by pySCENIC.**D.** Regulatory network of transcription factor CBX3 and its ferroptosis-associated target genes in CTCs.**E.** Quantification of lipid peroxidation levels by flow cytometry in H1975 and A375 cells with or without treatment of the ferroptosis inducer RSL3 following CBX3 KD, compared to control cells. Lipid peroxidation is measured using the BODIPY^TM^581/591 C11 molecular probe (Therom, USA, cat#D3861). Data are calculated based on three independent biological repeats and statistical significance was assessed by two-tailed Student’s t-test. **, *P*<0.01 and ***, *P*<0.001.**F. **Cell viability assay in H1975 and A375 following CBX3 KD, compared with Control cells. RSL3 as a ferroptosis inducer enhances CBX3 KD-increased ferroptosis (0.25 μM, 0.5 μM and 1 μM). The cell viability phenotype is rescued by lipophilic antioxidants, Ferrostatin-1 (2 μM). P values were calculated using a two-tailed Student’s t-test. ns, no significance, *, *P*<0.05,**, *P*<0.01, ***, *P*<0.001 and ****, *P*<0.0001.**G.** Violin plot showing the differential expression levels of ferroptosis resistance genes in CBX3-high and CBX3-low CTCs. The grouping of CTCs is based on mRNA expression levels of CBX3. CTCs were stratified into "CBX3 high" and "CBX3 low" groups based on the median value of CBX3 expression.**H.** RT-qPCR analysis showing the mRNA expression levels of *GPX4*, *G6PD*, *PCBP2 *and* NFE2L2 *in H1975 and A375 cells following CBX3 KD. Data are calculated based on three independent biological repeats and statistical significance was assessed by two-tailed Student’s t-test. ns, no significance, *, *P*<0.05,**, *P*<0.01 and ****, *P*<0.0001.**I.**Western blot analysis displaying the CBX3 and GPX4 expression in H1975 and A375 following CBX3 KD.**J. **Scatter plot showing the correlation between CBX3 expression and GPX4 expression in the HCC (liver cancer), BRCA (breast cancer) and SKCM (melanoma) dataset.**K.** ChIP-PCR assay was performed to detect the extent of CBX3 binding to the GPX4 promoter region in H1975 and A375 cells. P values were calculated using a two-tailed Student’s t-test **, *P*<0.01 and ***, *P*<0.001.**L.** Tumor metastasis model established by tail vein injection of control A375 cells expressing luciferase and CBX3-OE A375 cells expressing luciferase into immunocompromised NCG mice. The image shows IVIS imaging captured at week 1 and week 4. All images were adjusted to the same radiance scale.**M.** Bar plot shows the fluorescence values of whole body, lung and brain signal obtained from IVIS imaging captured at the week 1 and week 4 for the two experimental groups (NC and CBX3-OE groups). P values were calculated using a two-tailed Student’s t-test. *, *P*<0.05, **, *P*<0.01 and ***, *P*<0.001.**N.**H&E staining of lungs and brains dissected from two experimental groups (NC and CBX3-OE) of the metastatic mouse model (Top). mIHC images showing the expression of NG2 (green), GPX4 (yellow) and CBX3 (orange) in xenograft tumors in different groups (Bottom). Barplot showing the fraction of NG2^+^CBX3^+^GPX4^+^ cells among all DAPI+ cells in xenograft tumors in different groups. Y-axis, % of NG2^+^CBX3^+^GPX4^+^/DAPI^+^ cells. P values were calculated using a two-tailed Student’s t-test. *, *P*<0.05 and ***, *P*<0.001.**O.**Multi-immunofluorescence (mIF) staining of CTCs showing DAPI (nuclei), EPCAM (epithelial marker) or NG2 (melanoma marker), CBX3, and GPX4 (left). EPCAM^+^ cells were identified as lung CTCs and NG2^+^ cells was identified as melanoma CTCs. Barplot showing the number of CTCs, the number of CBX3^+^GPX4^+^ CTCs and the fraction of CBX3^+^GPX4^+^CTCs among all CTCs between non-metastasis and metastasis LUAD and SKCM patients. Data is calculated based on three independent non-metastasis patients and six independent metastasis patients were assessed by two-tailed Student’s t-test. **, *P*<0.01, ***, *P*<0.001 and ****, *P*<0.0001.**P.**Schematic illustration of the CTCs confers ferroptosis resistance through the CBX3/GPX4 axis to promote CTC survival and metastasis

These CTCs were enriched with Ferroptosis, but not Apoptosis signatures (Fig. 2A-B and S2A). CTCs exhibited elevated FTL and TFRC, and the “Iron uptake and transport pathway”, suggesting dysregulated iron homeostasis (Figure S2B-E). Transcription factor (TF) analysis identified CBX3 showing the most elevated TF activity score in CTCs, correlating with upregulated ferroptosis genes (Fig. 2**C-D**). Functionally, CBX3 knockdown (KD) in H1975 and A375 cells increased lipid peroxidation, elevated Fe^2+^/Fe^3+^, reduced GSH/GSSG, and enhanced susceptibility to RSL3-induced death, which was rescued by ferrostatin-1 (Fig. 2E-F, S2F, S3A-D), without affecting apoptosis (Figure S3E). Thus, CBX3 suppressed ferroptosis in CTCs.

The heterogenous CTCs were stratified into CBX3-high and low groups based on the median CBX3 expression. CBX3-high CTCs upregulated anti-ferroptosis genes including GPX4 (Fig. 2G). GPX4 expression decreased significantly after CBX3 KD (Fig. 2H-I). CBX3 and GPX4 expressions were positively correlated in several cancers (Fig. 2J). The binding of CBX3 at the GPX4 promoter was confirmed by ChIP-seq and ChIP-qPCR (Fig. 2K and S4A). CBX3 was reported to interact with EP300 to regulate gene transcription in glioblastoma [9]. Interestingly, both CBX3 and EP300 were elevated in CTCs (Fig. 2C). EP300 KD abolished GPX4 induction in CBX3 overexpression (OE) cells, and abrogated the binding of CBX3 to GPX4 promoter, indicating CBX3 co-operated with EP300 to modulate GPX4 expression (Figure S4B-C).

Oncogenic pathways like PI3K signaling were enriched in CBX3-high CTCs (Figure S5A). CBX3 KD impaired proliferation, clonogenicity, migration, and invasion (Figure S5B-D), whereas CBX3 OE enhanced these traits (Figure S5E-H). CBX3 OE A375 cells promoted metastases in immunocompromised mice (Fig. 2L-M). Multiplexed IHC confirmed elevated NG2^+^CBX3^+^GPX4^+^ metastases in lung, brain, heart, and spine of the OE group compared to NC controls (Fig. 2N and S6A). Thus, CBX3 promoted metastasis. In TCGA-LUAD cohort, CBX3 expression was correlated with overall survival and TNM stages (Figure S7A-B). In melanoma CTCs, high CBX3 was associated with disease progression (Figure S7C) [10]. Importantly, a prospective analysis of LUAD and melanoma patient cohort demonstrated that metastatic patients harbored significantly more CBX3^+^GPX4^+^ CTCs than non-metastatic cases (Fig. 2O, Table S4).

In conclusion, we identified CBX3 as a central driver conferring ferroptosis resistance in CTCs. CBX3^+^GPX4^+^ CTCs could serve as noninvasive biomarkers for the precise monitoring of metastatic progression (Fig. 2P). CBX3 may affect other stress-response pathways [11], and other TFs like SPI1 may regulate CTC biology. While our patient samples are limited, exploration using a larger patient cohort coupled with validations in CTC cell lines would provide deeper mechanistic insights into CTC-mediated metastasis.

## Supplementary Information

Supplementary Material 1

Supplementary Material 2

Supplementary Material 3

Supplementary Material 4

## Data Availability

scRNA-seq and whole exome sequencing raw data have been deposited in the Genome Sequence Archive in BIG Data Center, Beijing Institute of Genomics, Chinese Academy of Sciences under accession number HRA012575 that are publicly accessible at https://ngdc.cncb.ac.cn/gsa-human/s/L8gE89XL. Other data is provided within the manuscript or supplementary information files.

## Abbreviations

CTCs: Circulating Tumor Cells
LUAD: Lung Adenocarcinoma
HCC: Hepatocellular Carcinoma
BRCA: Breast Invasive Carcinoma
CNV: Copy Number Variation
CBX3: Chromobox 3
HP1: Heterochromatin Protein 1
CAF: Cancer-associated Fibroblasts
cDC: Conventional Dendritic Cells
pDC: Plasmacytoid Dendritic Cells
ADT: Antibody-derived Tags
WES: Whole-exome Sequencing
PD: Progressive Disease
RD: Responding Disease
