# Supplementary material for: CBX3 confers ferroptosis resistance during blood-borne metastasis
Source: J Hematol Oncol. 2026 Jan 15;19:9. doi: 10.1186/s13045-025-01777-0 (PMC12809899; doi:10.1186/s13045-025-01777-0)

Cell Line Authentication Service

STR Profiling Report

Testing Company: Shanghai QuiCell Biotechnology Co., Ltd

Sample Type: Cell Line

Testing Method: STR Genotyping

Report Time: 2025-11-4

COMPANY STATEMENT

1. THIS REPORT IS ONLY RESPONSIBLE FOR THE SAMPLES ANALYZED.
2. THE TESTING RESULTS AND THE ORGANIZATION NAME WILL NOT BE USED FOR ADVERTISEMENT, COMMERCIAL EXHIBITIONS, COMMERCIAL PERFORMANCE AND OTHER COMMERCIAL ACTIVITIES.
3. OBJECTIONS SHOULD BE RAISED WITHIN FIFTEEN DAYS AFTER THE RECEIPT OF THIS REPORT
4. THE PAPER REPORT WITH CONTENT ALTERING, ADDING OR WITHOUT THE STAMPED SEAL OF THE COMPANY ARE INVALID.

上海葵赛生物科技有限公司

Cell Line Authentication – STR Profiling Report

Sample code：

Table 1. Sample Code

Customer’ s code	Company Code
NCI-H1975	11-4-2

Sample Number:1

Sample Type: Cell line

Testing Type: STR

Testing Method:

DNA was extracted with Axygen's genome extraction kit, amplified by 21-STR amplification protocol, and the STR locus and sex gene Amelogenin were detected on ABI 3730XL genetic analyzer.

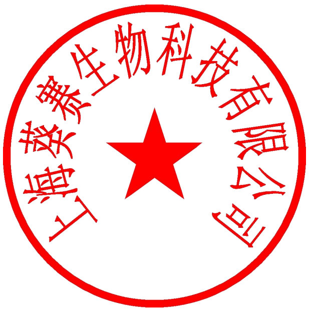

Test Results

1. STR profile

Table 2. STR and Amelogenin Genotyping Results of Cell line 11-4-2						
Loci	Sample information			Cell Bank information		
	Sample name: NCI-H1975			Cell line name: NCI-H1975		
	Allele1	Allele2	Allele3	Allele1	Allele2	Allele3
D3S1358	14	15		14	15	
vWA	18			18		
D7S820	8	11		8	11	
CSF1PO	12			12		
Penta E	12	16		12	16	
D8S1179	13	16		13	16	
D21S11	28			28		
D16S539	9	12		9	12	
D2S1338	17			17		
Penta D	12	13		12	13	
D19S433	15	15.2		15	15.2	
TH01	7			7		
D13S317	10			10	13	
TPOX	8	11		8	11	
D18S51	13			13		
D6S1043	12					
AMEL	X			X		
D1S1656	14			14	15	
D5S818	11	12		11	12	
D12S391	17			17		
FGA	21	24		21	24	

2. database annotation

Figure 1. STR matching analysis

Accession	Name	Nº Markers	Score	Amel	CSF1PO	D1S1656	D2S1338	D3S1358	D5S818	D6S1043	D7S820	D8S1179	D12S391	D13S317	D16S539	D18S51	D19S433	D21S11	FGA	Penta D	Penta E	TH01	TPOX	vWA
NA	Query	NA	NA	X	12	14	17	14,15	11,12	12	8,11	13,16	17	10	9,12	13	15,15.2	28	21,24	12,13	12,16	7	8,11	18
CVCL_1511 *Best*	NCI-H1975	19	96.67%	X	12	14,15	17	14,15	11,12		8,11	13,16	17	10,13	9,12	13	15,15.2	28	21,24	12,13	12,16	7	8,11	18

Note: The STR online match analysis of the test cell against EXPASY database, showing cell number (Cell No.) and cell name.

3. Authentication

☑The DNA typing of this cell line was basically matched in the cell line search. The EXPASY database shows that the cell name is**NCI-H1975** and the cell number corresponds to **CVCL_1511**. No multiple alleles were found in this cell line during this test.

Appendix:

1. Genotyping Strategy and Site Distribution

Table S1. Experimental Strategy and Sites

	Strategy 1	Strategy 2	Strategy 3	Strategy 4
1	D3S1358	D8S1179	D19S433	AMEL
2	VWA	D21S11	TH01	D1S1656
3	D7S820	D16S539	D13S317	D5S818
4	CSF1PO	D2S1338	TPOX	D12S391
5	PENTAE	PENTAD	D18S51	FGA
6			D6S1043	

The allele match algorithm compares the 8 core loci plus amelogenin only, even though alleles from all lociwill be reported when available.

2. EXPASY tools was used to carry on the cell line comparison, which contains 2455 cell lines STR data from ATCC, DSMZ, JCRB ,ECACC， GNE and RIKEN databases. If the cell is not included in the above cell library, users need to compared with other databases.

Applied Biosystems
GeneMapper® ID-X 1.5

Project: 11-4-2

Sample Name	SQ
NCI-H1975	

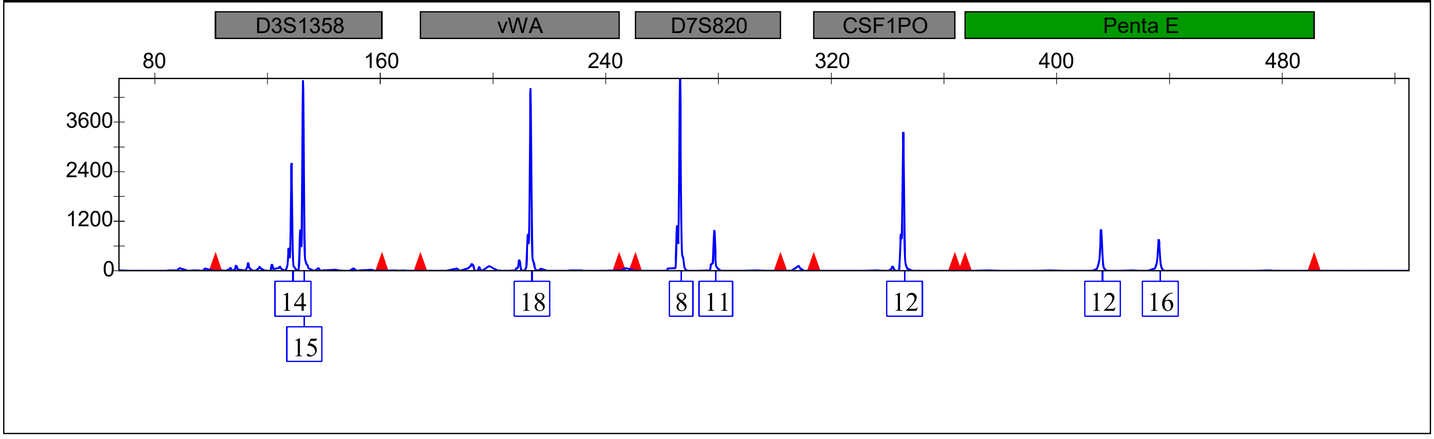

NCI-H1975

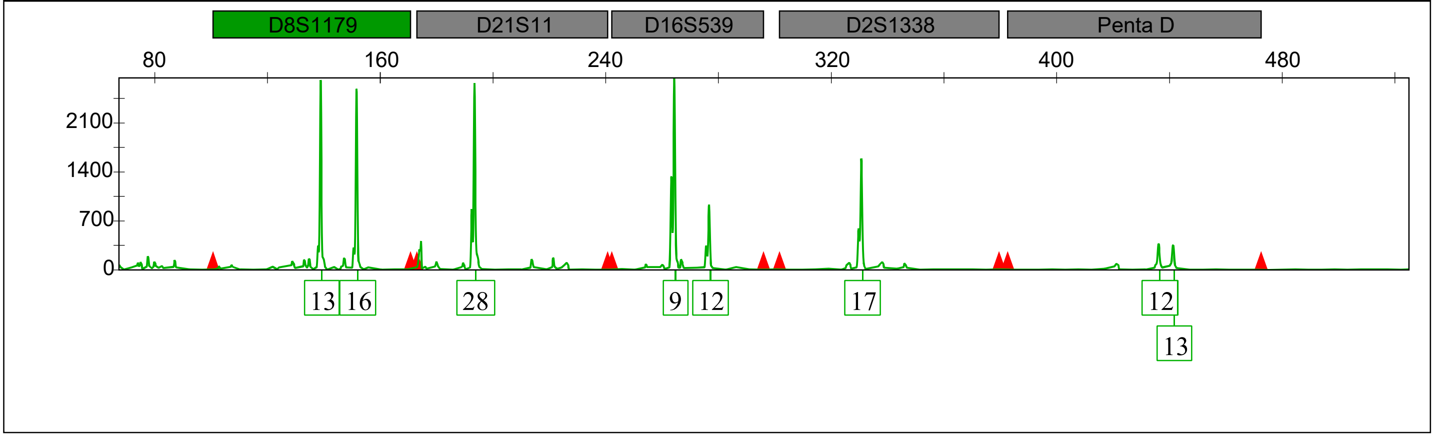

NCI-H1975

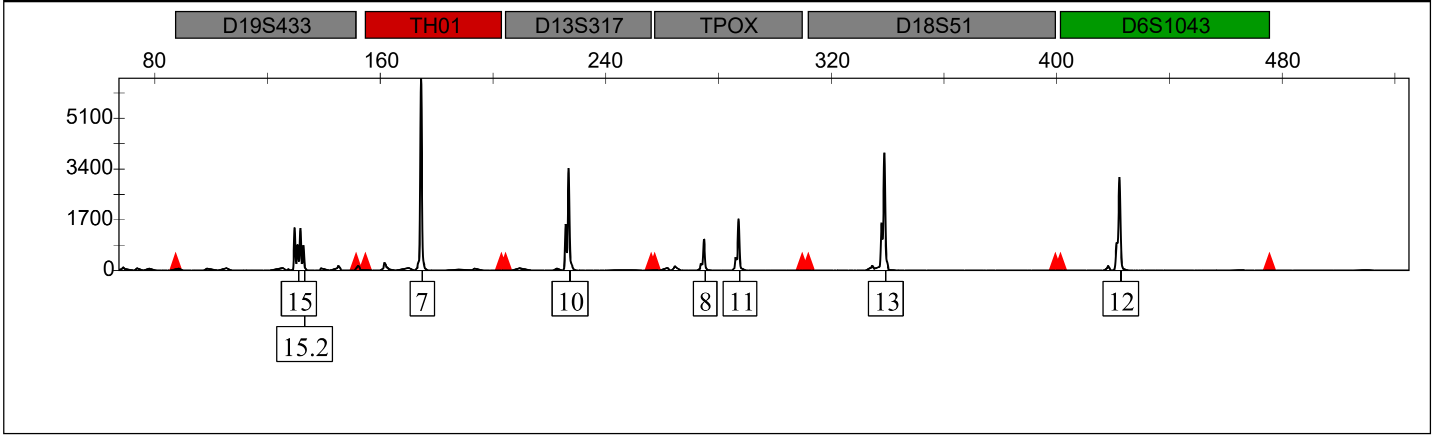

NCI-H1975

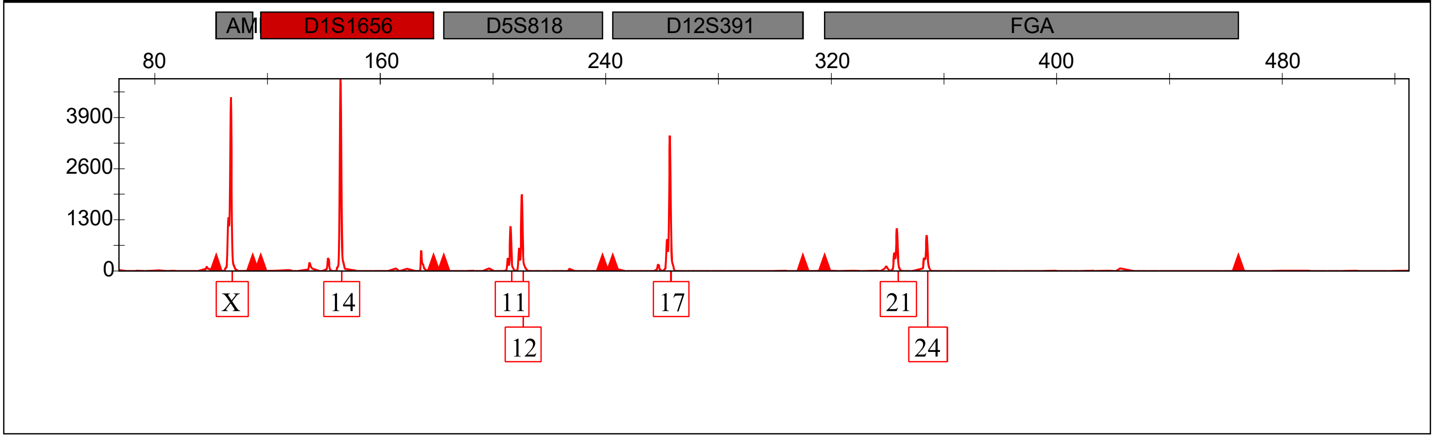

Cell Line Authentication Service

STR Profiling Report

Testing Company: Shanghai QuiCell Biotechnology Co., Ltd

Sample Type: Cell Line

Testing Method: STR Genotyping

Report Time: 2025-9-29

COMPANY STATEMENT

1. THIS REPORT IS ONLY RESPONSIBLE FOR THE SAMPLES ANALYZED.
2. THE TESTING RESULTS AND THE ORGANIZATION NAME WILL NOT BE USED FOR ADVERTISEMENT, COMMERCIAL EXHIBITIONS, COMMERCIAL PERFORMANCE AND OTHER COMMERCIAL ACTIVITIES.
3. OBJECTIONS SHOULD BE RAISED WITHIN FIFTEEN DAYS AFTER THE RECEIPT OF THIS REPORT
4. THE PAPER REPORT WITH CONTENT ALTERING, ADDING OR WITHOUT THE STAMPED SEAL OF THE COMPANY ARE INVALID.

Cell Line Authentication – STR Profiling Report

Sample code：

Table 1. Sample Code

Customer’ s code	Company Code
A375	2025-9-29-LC

Sample Number:1

Sample Type: Cell line

Testing Type: STR

Testing Method:

DNA was extracted with Axygen's genome extraction kit, amplified by 21-STR amplification protocol, and the STR locus and sex gene Amelogenin were detected on ABI 3730XL genetic analyzer.

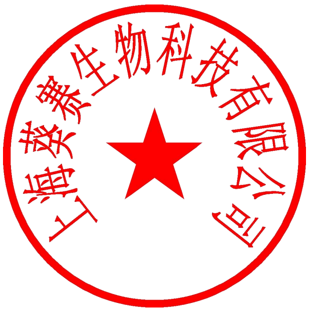

Test Results

1. STR profile

Table 2. STR and Amelogenin Genotyping Results of Cell line 2025-9-29-LC						
Loci	Sample information			Cell Bank information		
	Sample name: A375			Cell line name: A-375		
	Allele1	Allele2	Allele3	Allele1	Allele2	Allele3
D3S1358	15	17		15	17	
vWA	16	17		16	17	
D7S820	9			9		
CSF1PO	11	12		11	12	
Penta E	10	12		10	12	
D8S1179	11	14		11	14	
D21S11	29	30		29	30	
D16S539	9			9		
D2S1338	16	24		16	24	
Penta D	9	15		9	15	
D19S433	13	14.2		13	14.2	
TH01	8			8		
D13S317	11	14		11	14	
TPOX	8	10		8	10	
D18S51	12	17		12	17	
D6S1043	11	14				
AMEL	X			X		
D1S1656	16	17.3		16	17.3	
D5S818	12			12		
D12S391	18	21		18	21	
FGA	23			23		

2. database annotation

Figure 1. STR matching analysis

Accession	Name	Nº Markers	Score	Amel	CSF1PO	D1S1656	D2S1338	D3S1358	D5S818	D6S1043	D7S820	D8S1179	D12S391	D13S317	D16S539	D18S51	D19S433	D21S11	FGA	Penta D	Penta E	TH01	TPOX	vWA
NA	Query	NA	NA	X	11,12	16,17.3	16,24	15,17	12	11,14	9	11,14	18,21	11,14	9	12,17	13,14.2	29,30	23	9,15	10,12	8	8,10	16,17
CVCL_0132	A-375	19	100.00%	X	11,12	16,17.3	16,24	15,17	12		9	11,14	18,21	11,14	9	12,17	13,14.2	29,30	23	9,15	10,12	8	8,10	16,17

Note: The STR online match analysis of the test cell against EXPASY database, showing cell number (Cell No.) and cell name.

3. Authentication

☑The DNA typing of this cell line was completely matched in the cell line search. The EXPASY database shows that the cell name is **A-375** and the cell number corresponds to **CVCL_0132** No multiple alleles were found in this cell line during this test.

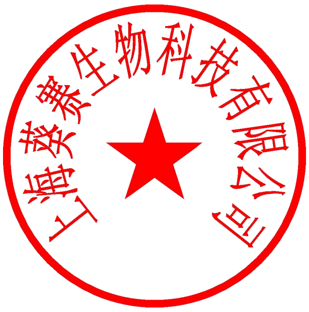

Appendix:

1. Genotyping Strategy and Site Distribution

Table S1. Experimental Strategy and Sites

	Strategy 1	Strategy 2	Strategy 3	Strategy 4
1	D3S1358	D8S1179	D19S433	AMEL
2	VWA	D21S11	TH01	D1S1656
3	D7S820	D16S539	D13S317	D5S818
4	CSF1PO	D2S1338	TPOX	D12S391
5	PENTAE	PENTAD	D18S51	FGA
6			D6S1043	

The allele match algorithm compares the 8 core loci plus amelogenin only, even though alleles from all lociwill be reported when available.

2. EXPASY tools was used to carry on the cell line comparison, which contains 2455 cell lines STR data from ATCC, DSMZ, JCRB ,ECACC，GNE and RIKEN databases. If the cell is not included in the above cell library, users need to compared with other databases.

Sample Name	SQ

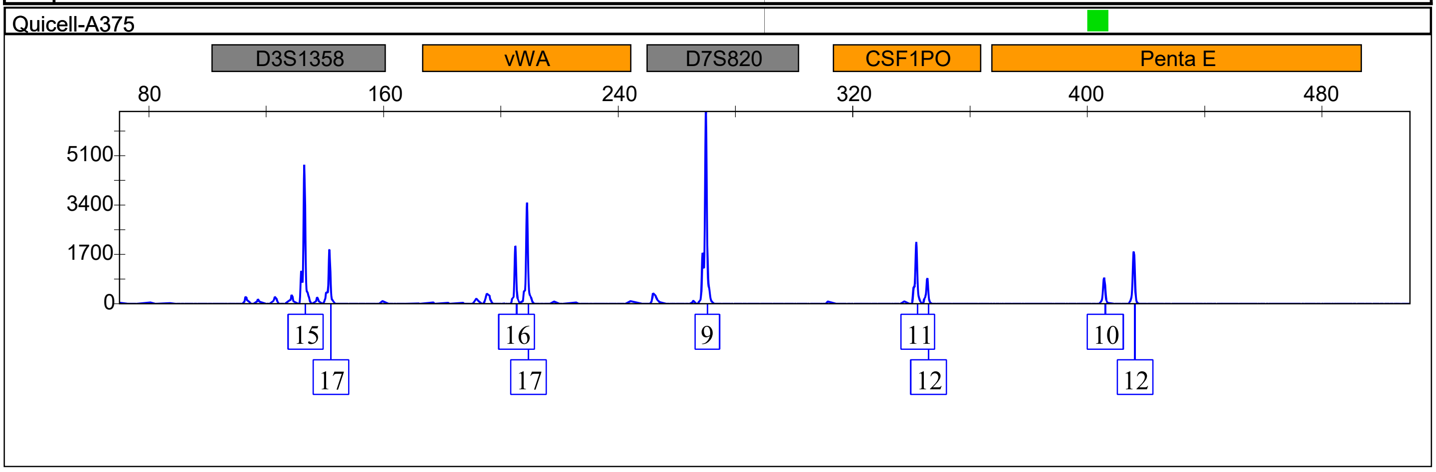

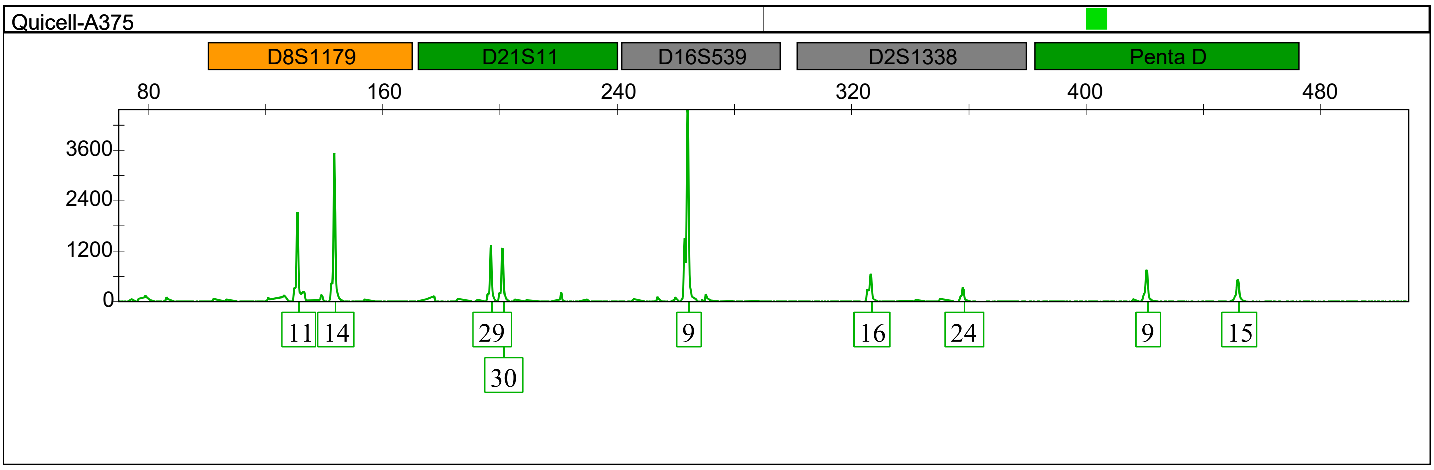

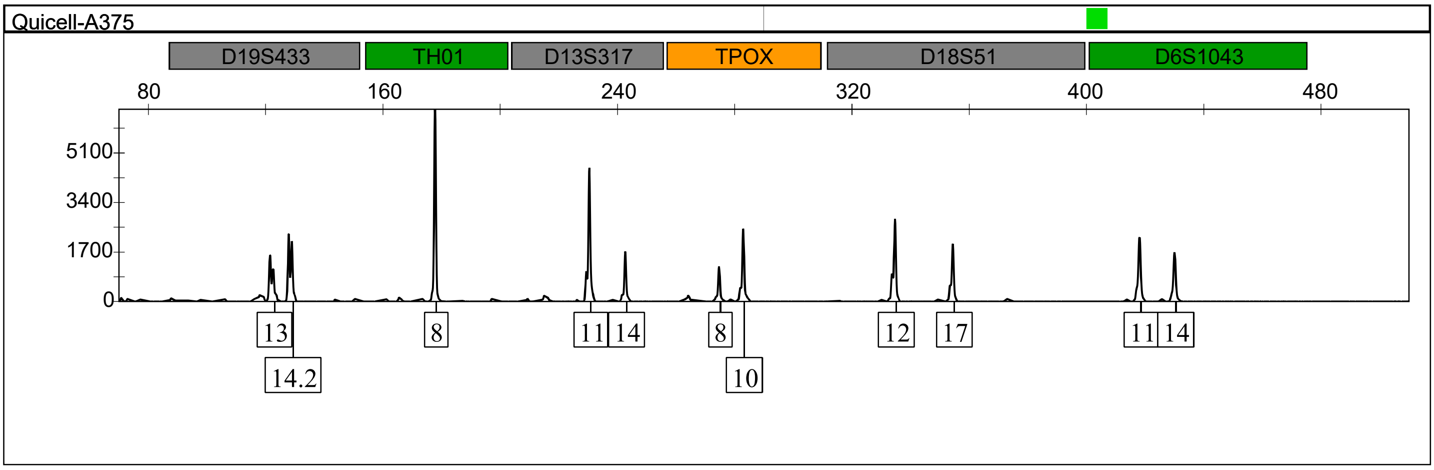

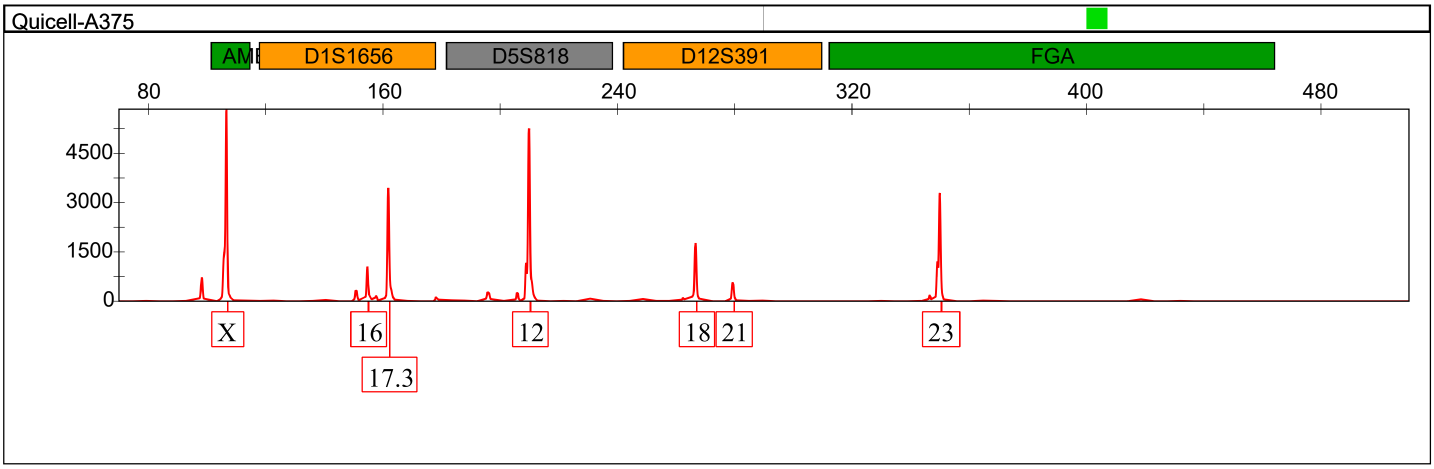

Supplement: Supplementary file 1 — Supplementary Material 1 [file 13045_2025_1777_MOESM1_ESM.pdf]
