# Supplementary material for: CBX3 confers ferroptosis resistance during blood-borne metastasis
Source: J Hematol Oncol. 2026 Jan 15;19:9. doi: 10.1186/s13045-025-01777-0 (PMC12809899; doi:10.1186/s13045-025-01777-0)
Supplement: Supplementary file 2 — Supplementary Material 2 [file 13045_2025_1777_MOESM2_ESM.docx]

**CBX3 confers** **f****erroptosis resistance during blood-borne metastasis**

Chun Wu^1,2, #^, Xuefei Liu^1, 3, #^, Boxi Zhao^1, #^, Mao Zhao^4^, Binyu Zhang^1,5^, Guanyin Huang^1^, Yixin Cheng^2^, Shuqian Zheng^1^, Jianyang Hu^1^, Ling Guo^2^, Weinan Guo^4,^*, Jun Tan^6,7^*, Xin Hong^1,8,9, 10,^ *

**Content**

[Supplementary figures 2](#_Toc217468296)

[Supplementary tables 14](#_Toc217468297)

[Table S1. Clinical characteristics of patients for single-cell RNA sequencing. 14](#_Toc217468298)

[Table S2. List of genes significantly elevated in CTCs. 14](#_Toc217468299)

[Table S3. The mRNA expression of CTCs. 15](#_Toc217468300)

[Table S4. Clinical characteristics of a prospective cohort of patients for CTC isolation and characterization. 23](#_Toc217468301)

[Table S5. List of siRNA sequences. 24](#_Toc217468302)

[Table S6. List of antibody catalog numbers. 24](#_Toc217468303)

[Table S7. List of qRT-PCR primer sequences. 24](#_Toc217468304)

## Supplementary figures

**
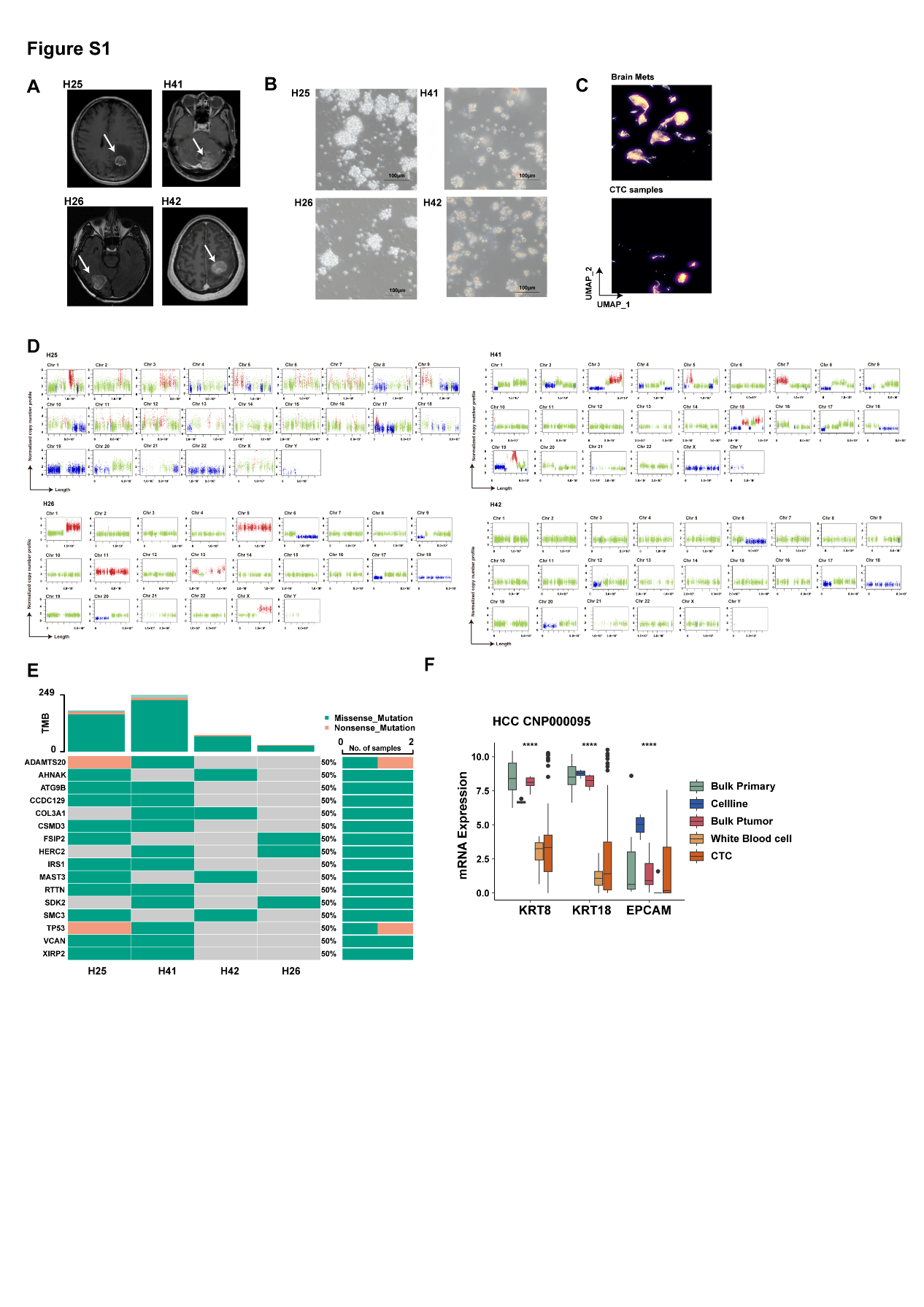
**

**Figure S1 Microfluidic isolation and molecular characterization of CTCs from LUAD patients with BrM.**

**A.** Brain CT imaging reveals an intracranial space-occupying lesion.

**B.** Representative images of CTCs from in-house LC-BM patients. Scale bars, 100μm.

**C.** Density plot showing the distribution of brain metastases and microfluidically-enriched CTC samples.

**D.** Somatic copy number variation (SCNV) analysis by FREEC identifies genomic variations in WES data. Y axis, normalized copy number profile. X axis, each chromosome length. Red: amplifications (Gain); blue: deletions (Loss); green: Normal.

**E.** Waterfall plot of the distribution of mutations found in patients with BM by whole-exome sequencing (WES).

**F.** Box plot showing the mRNA expression level of classic epithelial cells (*KRT8*, *KRT18*, *EPCAM*) markers among primary lung tumor tissues, paracancerous lung tumor tissues, tumor cell line, white blood cells and CTCs in CNP000095 dataset. P values were calculated using one-way ANOVA test. ****, *P < 0.0001*.


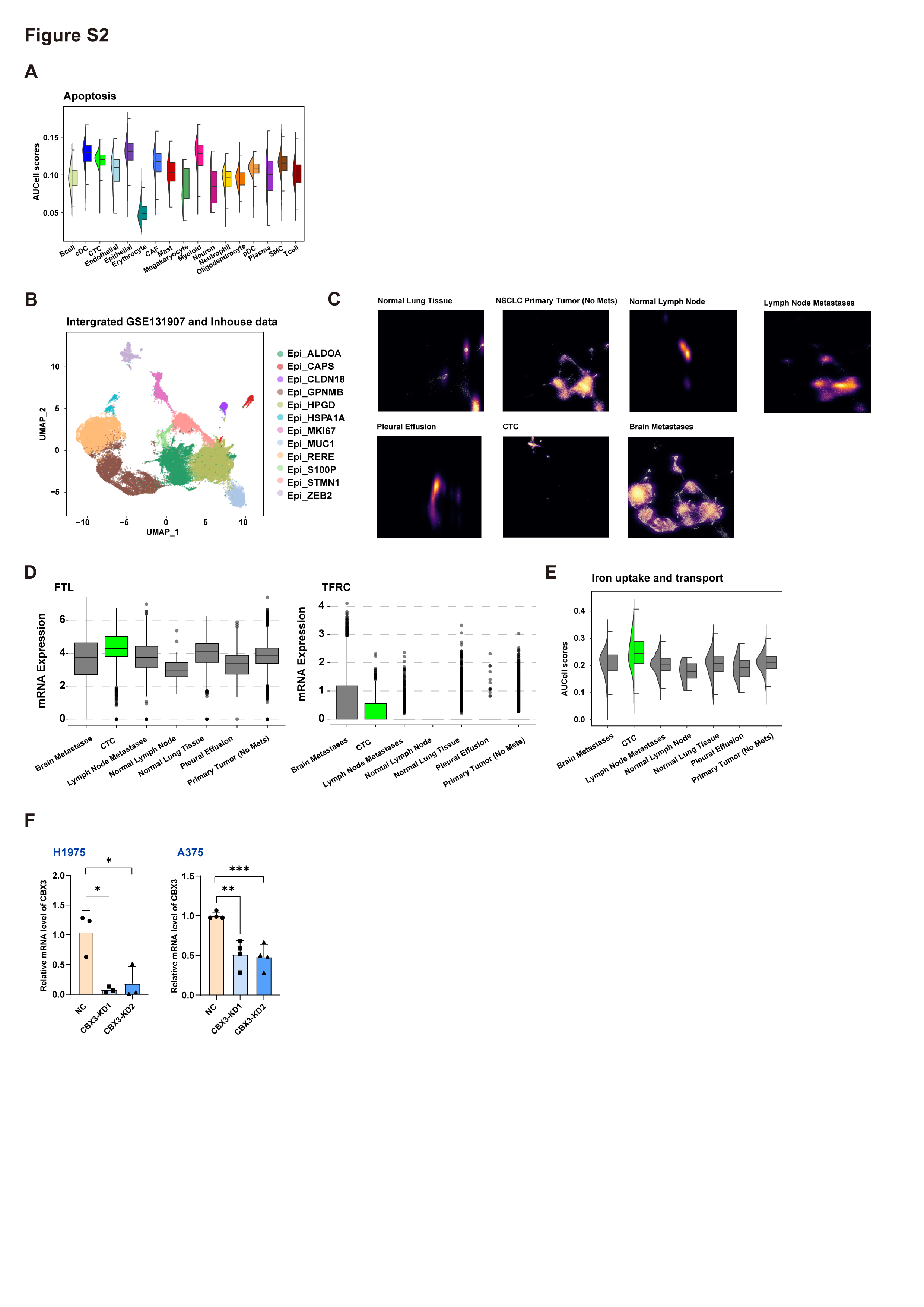


**Figure S2** **CTCs were in a state of iron homeostasis dysregulation with potential iron accumulation**

**A.** Box plot showing the apoptosis scores among the all-cell subtypes. Apoptosis scores were calculated using AUCell.

**B.** UMAP plot showing the subtypes of epithelial cells, each dot indicated a single cell. Color-coded for the cell type. All cells were from Inhouse data and GSE131907.

**C.** Density plot showing the distribution of normal lung tissue, non-metastatic primary lung adenocarcinoma (LUAD), normal lymph nodes, LUAD lymph node metastases, pleural effusions, CTCs, and LUAD brain metastases.

1. Boxplot showing FTL and TFRC mRNA levels across different tissues.
2. Box plot showing the iron uptake and transport pathway scores among different tissues. The iron uptake and transport pathway scores were calculated using AUCell.

**F.** RT-qPCR analysis showing the mRNA expression levels of *CBX3* in H1975 and A375 cells following CBX3 KD. Data are calculated based on three independent biological repeats and statistical significance was assessed by two-tailed Student’s t-test. *, *P*<0.05, **, *P*<0.01 and ***, *P*<0.001.


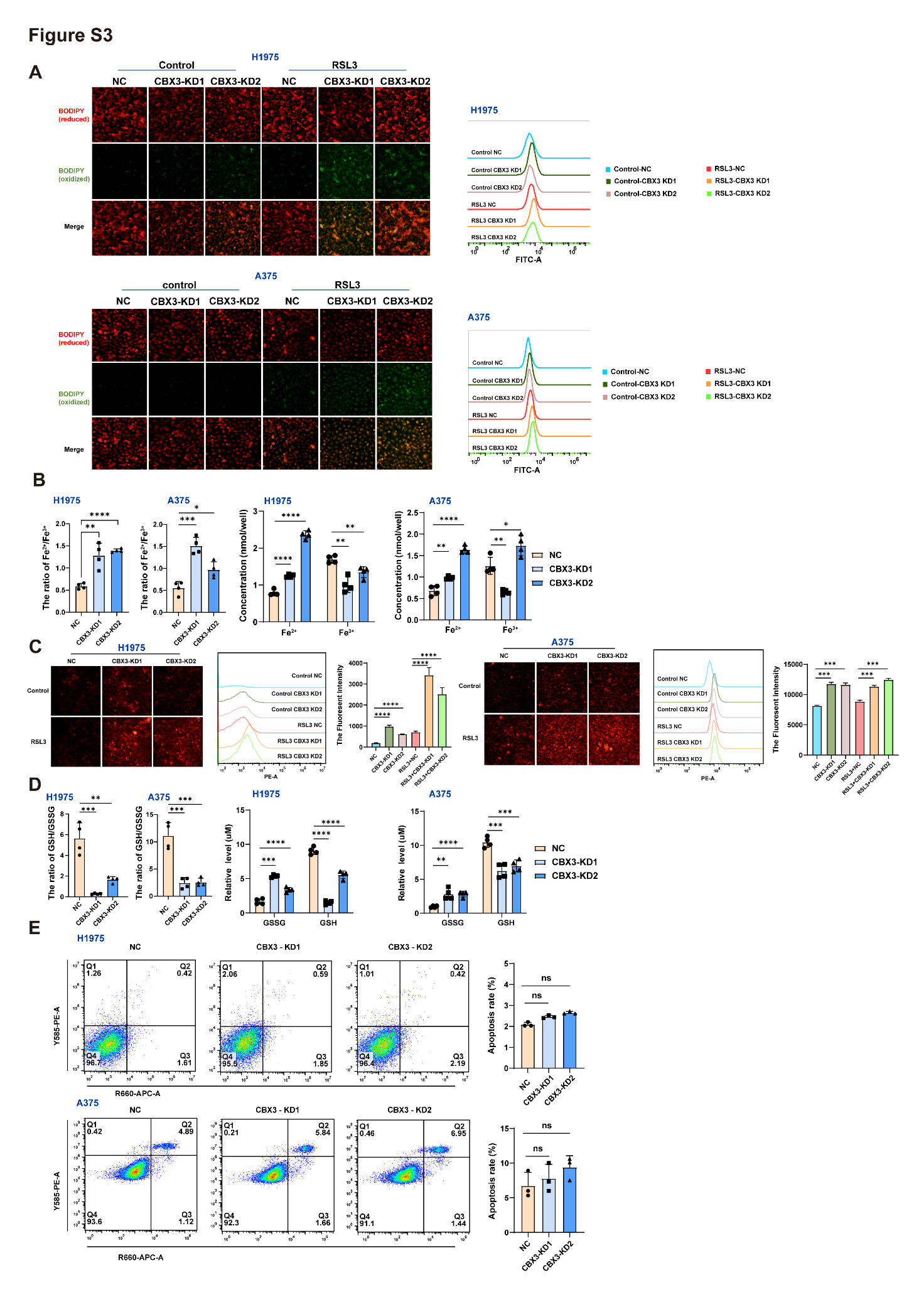


**Figure S3 CBX3 promotes ferroptosis resistance.**

**A.** Lipid ROS was measured by BODIPY^TM^ 581/591 C11 staining coupled to flow cytometry in CBX3-KD H1975 and A375 cells treated with or without the ferroptosis inducer RSL3. The left panel shows fluorescent images, the middle panel displays flow cytometry histograms, and the right panel presents quantitative analysis of flow cytometry. Data are calculated based on three independent biological repeats and statistical significance was assessed by two-tailed Student’s t-test.  **, *P*<0.01, ***, *P*<0.001 and ****, *P*<0.0001.

**B.** Increased the ratio of Fe^2+^/Fe^3+^ in CBX3 KD H1975 and A375, compared with vector Control. Y axis, relative ratio between Fe^2+^/Fe^3+^ levels (Left panel). The Fe^2+^ and Fe^3+^ concentration (mol/well) in CBX3 KD H1975 and A375, compared with vector Control. Y axis, the Fe^2+^ and Fe^3+^ concentration (Right panel). Data are calculated based on four independent biological repeats and statistical significance was assessed by two-tailed Student’s t-test. *, *P*<0.05, **, *P*<0.01, ***, *P*<0.001 and ****, *P*<0.0001.

**C.** Ferrous iron (Fe^2+^) levels were detected using a fluorescent probe (red fluorescence) staining coupled to flow cytometry in CBX3-KD H1975 and A375 cells treated with or without the ferroptosis inducer RSL3. The left panel shows fluorescent images, the middle panel displays flow cytometry histograms, and the right panel presents quantitative analysis of flow cytometry. Data are calculated based on three independent biological repeats and statistical significance was assessed by two-tailed Student’s t-test.  **, *P*<0.01, ***, *P*<0.001 and ****, *P*<0.0001.

**D.** Reduced the ratio of GSH/GSSG in CBX3 KD (KD) H1975 and A375, compared with vector Control. Y axis, relative ratio between GSH/GSSG levels (Left panel). The relative level of GSH and GSSG (μM) in CBX3 KD (KD) H1975 and A375, compared with vector Control. Y axis, the relative level of GSH and GSSG (Right panel). Data are calculated based on four independent biological repeats and statistical significance was assessed by two-tailed Student’s t-test.  **, *P*<0.01, ***, *P*<0.001 and ****, *P*<0.0001.

**E.** Apoptosis level and relative quantitative analysis of CBX3-NC and CBX3-KD cells detected by flow cytometry. Data are calculated based on three independent biological repeats and statistical significance was assessed by two-tailed Student’s t-test.  ns, no significance.


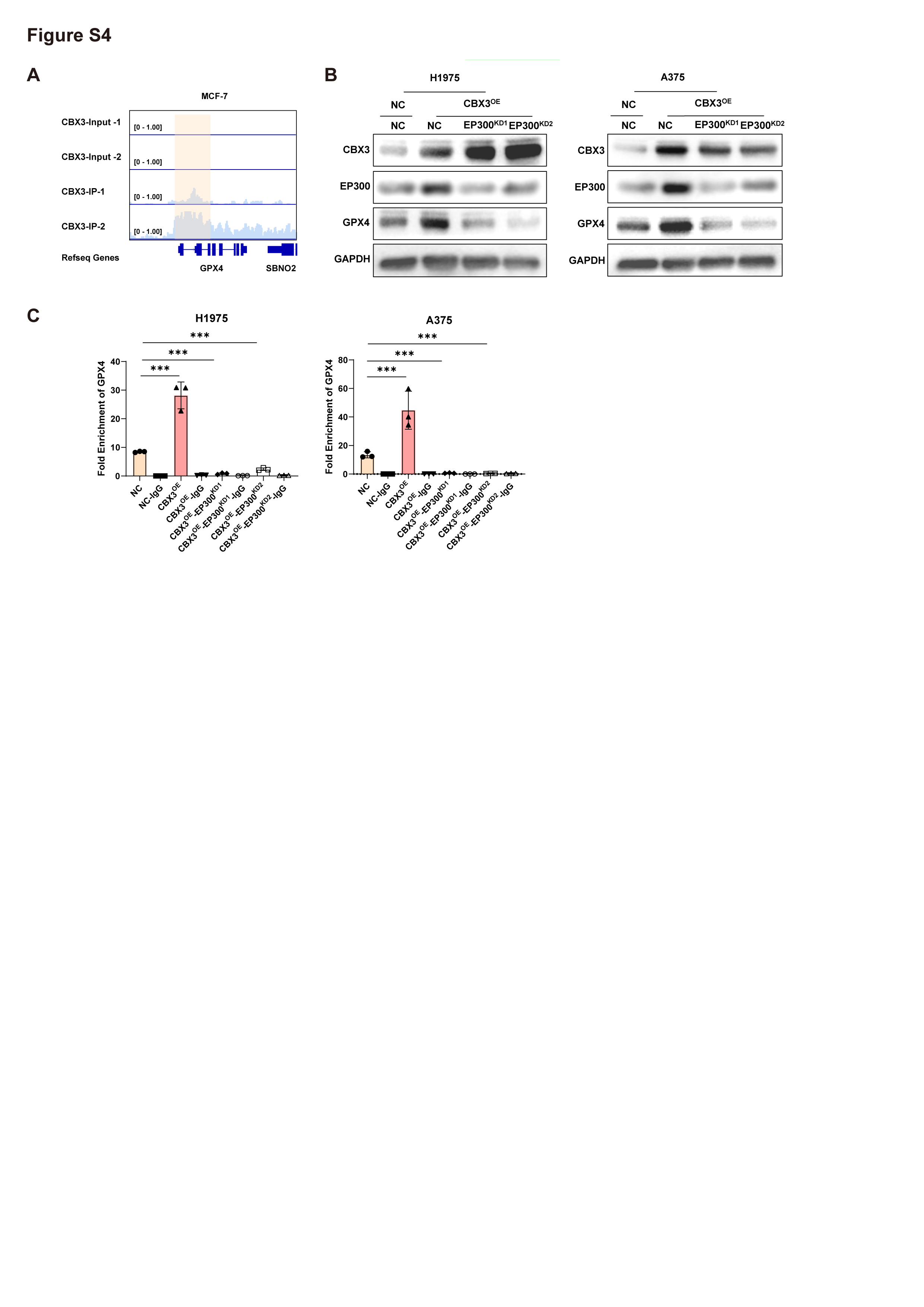


**Figure S4 CBX3 cooperatively regulates GPX4 by recruiting the co-factor EP300.**

**A.** Binding of CBX3 to the GPX4 gene in MCF7 cell line. All peaks were called by MACS2 (*P* < 0.05).

**B.** Western blot analysis showing the effect of NC, CBX3 overexpression, and CBX3 overexpression with EP300 knockdown conditions on the protein expression level of CBX3, EP300 and GPX4 in H1975 and A375 cells.

**C.**CHIP-qPCR assay was performed to assess CBX3 binding at the GPX4 locus in H1975 and A375 cells under control (NC), CBX3 overexpression (CBX3-OE), and CBX3 overexpression combined with EP300 knockdown (CBX3-OE + EP300-KD) conditions. Data are calculated based on three independent biological repeats and statistical significance was assessed by two-tailed Student’s t-test.  ***, *P*<0.001

**
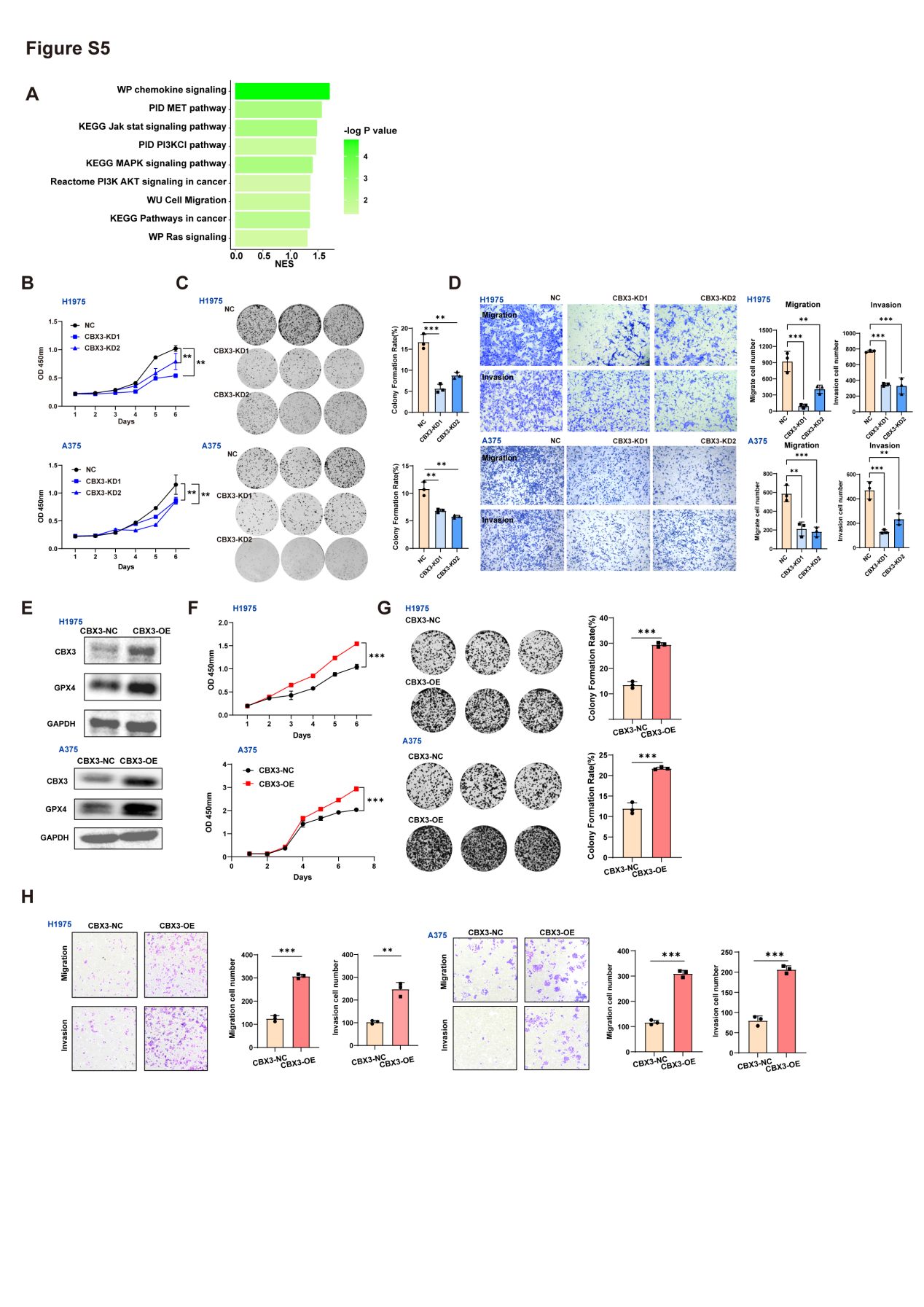
**

**Figure S5 Functional characterization of CBX3 KD and overexpression (CBX3 OE) in H1975 and A373 cells.**

**A.** Bar graph showing the pathway enriched in the CBX3 high CTCs. The x-axis indicates the Normalized Enrichment score (NES).The color indicates the -log10 P value.

**B.** CCK-8 assay showing the effect of CBX3 KD on the proliferation of H1975 and A375 cells, demonstrating reduced cell growth upon CBX3 KD. Data are calculated based on three independent biological repeats and statistical significance was assessed by two-tailed Student’s t-test.  **, *P*<0.01

**C.** The colony formation rate of NC cells and CBX3-KD cells of H1975 and A375. Data are calculated based on three independent biological repeats and statistical significance was assessed by two-tailed Student’s t-test.  **, *P*<0.01 and ***, *P*<0.001.

**D.** Transwell migration and invasion assays assessing the impact of CBX3 KD on the migration and invasion abilities of H1975 and A375 cells, with significant inhibition observed in both cell lines. Data are calculated based on three independent biological repeats and statistical significance was assessed by two-tailed Student’s t-test.  **, *P*<0.01 and ***, *P*<0.001.

**E.** Western blot analysis displaying the CBX3 and GPX4 expression in H1975 and A375 following CBX3 overexpression.

**F.** CCK-8 assay showing the effect of CBX3 overexpression (CBX3-OE) on the proliferation of H1975 and A375 cells, demonstrating induced cell growth upon CBX3 overexpression. Data are calculated based on three independent biological repeats and statistical significance was assessed by two-tailed Student’s t-test.  ***, *P*<0.001

**G.** The colony formation rate of NC cells and CBX3-OE cells of H1975 and A375. Data are calculated based on three independent biological repeats and statistical significance was assessed by two-tailed Student’s t-test.  ***, *P*<0.001.

**H.** Transwell migration and invasion assays assessing the impact of CBX3-OE on the migration and invasion abilities of H1975 and A375 cells, with significant stimulation observed in both cell lines. Data are calculated based on three independent biological repeats and statistical significance was assessed by two-tailed Student’s t-test.  **, *P*<0.01 and ***, *P*<0.001.


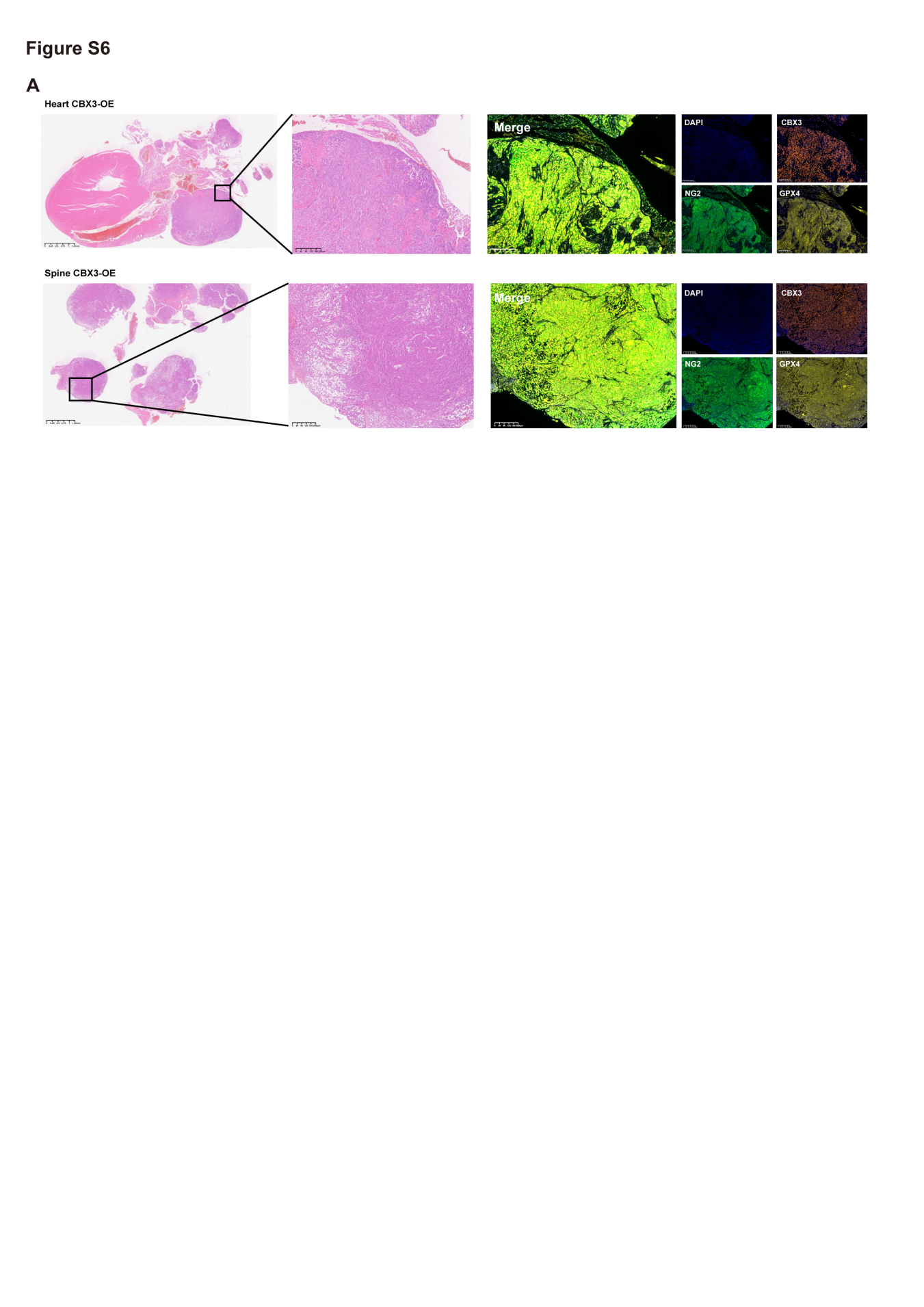


**Figure S6 H&E and mIHC analyses of metastatic lesions in heart and spine from the CBX3 OE group.**

1. H&E staining of heart and spine dissected from CBX3-OE of the metastatic mouse model (Left). mIHC images showing the expression of NG2 (green), GPX4 (yellow) and CBX3 (orange) in xenograft tumors in different tissues (Right).


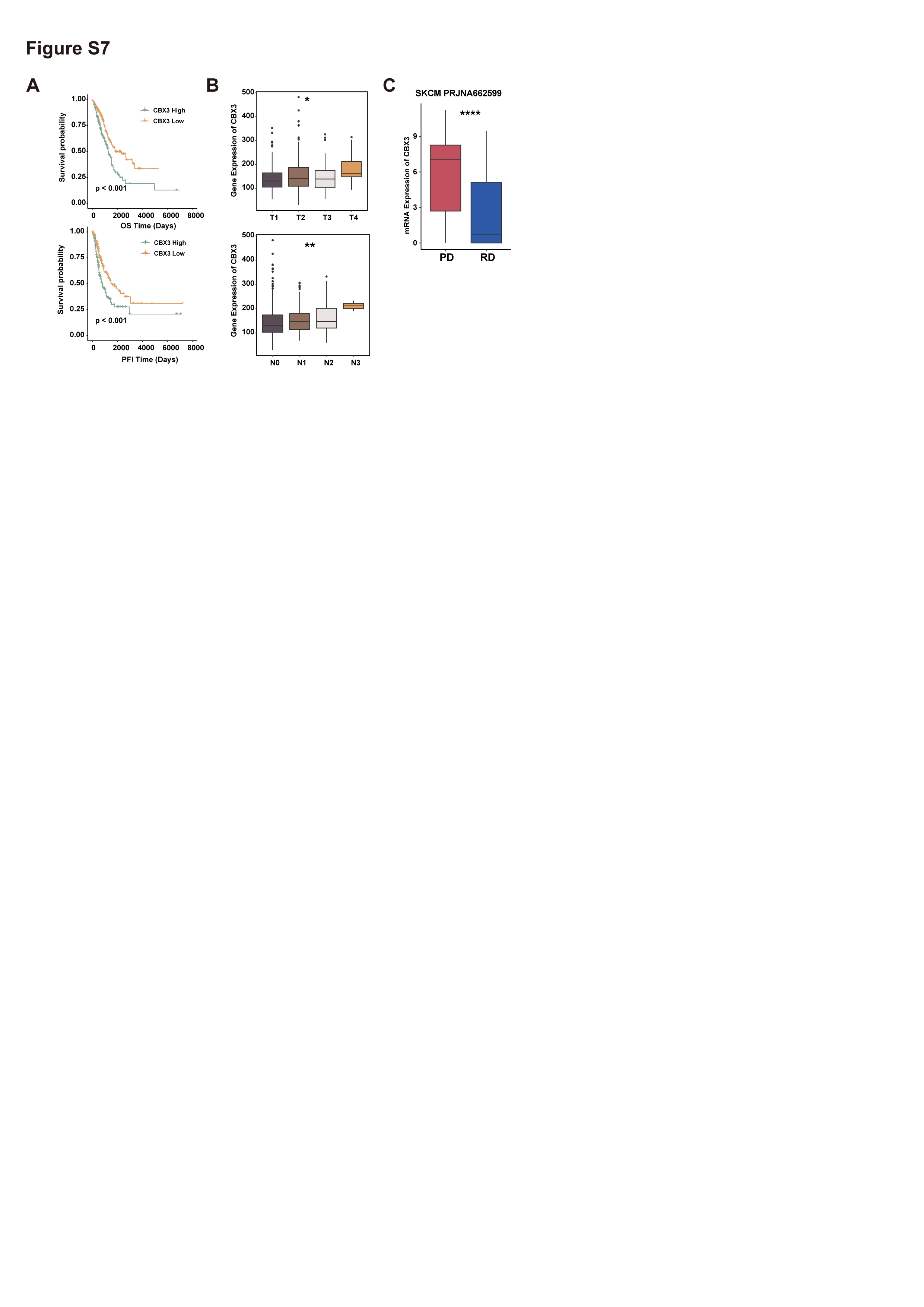


**Figure S7 Correlational analysis of CBX3 expression and clinical outcome.**

**A.** Kaplan-Meier estimation of overall survival (OS) and progression-free interval (PFI) time of patients in The Cancer Genome Atlas (TCGA)-LUAD dataset based on the expression level of CBX3.

**B.** Box plot showing the comparison of *CBX3* mRNA expression in different tumor stage (TCGA-LUAD cohort).

**C.** Box plot showing the expression levels of *CBX3* in primary CTCs derived from patients with responding disease (RD) or progressing disease (PD) in SKCM dataset. P values were calculated using a two-sided paired Wilcoxon signed-rank test. ****, *P*<0.0001.

## Supplementary tables

### Table S1. Clinical characteristics of patients for single-cell RNA sequencing.

| **Patient** | **Dataset** | **SC - Brain metastasis** | **WES (PBMC+ Brain metastasis)** | **Whole blood (CTC isolation)** | **Age** | **Gender** | **Smoking** | **Lung cancer subtype** |
| --- | --- | --- | --- | --- | --- | --- | --- | --- |
| H25 | Inhouse | √ | √ | √ | 58 | male | √ | Adenocarcinoma |
| H26 | Inhouse | √ | √ | √ | 35 | male | √ | Adenocarcinoma |
| H41 | Inhouse | √ | √ | √ | 67 | male | √ | Squamous cell carcinoma |
| H42 | Inhouse | √ | √ | √ | 53 | female | X | Adenocarcinoma |

*** SC - Brain metastasis:** **Single-cell RNA sequencing (scRNA-seq) of brain metastatic lesions**

****WES: whole-exome sequencing**

### Table S2. List of genes significantly elevated in CTCs.

| **feature** | **group** | **avgExpr** | **logFC** | **statistic** | **auc** | **pval** | **padj** | **pct_in** | **pct_out** |
| --- | --- | --- | --- | --- | --- | --- | --- | --- | --- |
| S100A8 | CTC | 3.296975 | 2.859429 | 43831301 | 0.925795 | 0 | 0 | 96.2706 | 20.62247 |
| S100A9 | CTC | 3.467282 | 2.504693 | 41026463 | 0.866552 | 0 | 0 | 96.53079 | 38.01081 |
| S100A4 | CTC | 3.464748 | 2.373003 | 43098426 | 0.910316 | 0 | 0 | 97.83174 | 58.31669 |
| CTSS | CTC | 2.615465 | 2.068222 | 43125908 | 0.910896 | 0 | 0 | 93.32177 | 40.30003 |
| VCAN | CTC | 2.167251 | 2.038011 | 42081998 | 0.888847 | 0 | 0 | 83.0876 | 8.867079 |
| MNDA | CTC | 2.249369 | 2.033133 | 43933741 | 0.927959 | 0 | 0 | 92.5412 | 13.09727 |
| ZEB2 | CTC | 2.453509 | 1.801329 | 41070829 | 0.867489 | 0 | 0 | 93.49523 | 32.19522 |
| VIM | CTC | 3.231655 | 1.782991 | 40628885 | 0.858155 | 0 | 0 | 97.83174 | 69.05411 |
| IFI30 | CTC | 2.689813 | 1.755737 | 40427048 | 0.853891 | 0 | 0 | 93.58196 | 55.14831 |
| SH3BGRL3 | CTC | 2.80115 | 1.734514 | 42195529 | 0.891245 | 0 | 0 | 96.79098 | 69.93327 |
| AIF1 | CTC | 1.966948 | 1.635486 | 41871834 | 0.884408 | 0 | 0 | 91.4137 | 17.5442 |
| PLXDC2 | CTC | 2.215469 | 1.631174 | 40706464 | 0.859793 | 0 | 0 | 90.63313 | 36.81262 |
| FCER1G | CTC | 2.094448 | 1.627363 | 40887033 | 0.863607 | 0 | 0 | 93.66869 | 23.12113 |
| SRGN | CTC | 2.695531 | 1.599265 | 37755323 | 0.79746 | 6.28E-302 | 3.36E-300 | 95.31657 | 47.16526 |
| CYBB | CTC | 1.852484 | 1.592982 | 42106956 | 0.889374 | 0 | 0 | 89.07199 | 15.35726 |
| LCP1 | CTC | 2.028563 | 1.567804 | 41854955 | 0.884051 | 0 | 0 | 95.05637 | 27.1565 |
| CYP1B1 | CTC | 1.556223 | 1.460568 | 40067880 | 0.846305 | 0 | 0 | 74.76149 | 9.531927 |
| ITGB2 | CTC | 1.756898 | 1.443852 | 42030235 | 0.887754 | 0 | 0 | 91.4137 | 21.14364 |
| CST3 | CTC | 2.452752 | 1.4225 | 38597147 | 0.815241 | 3.64E-306 | 2.00E-304 | 94.18907 | 63.87658 |
| NCF2 | CTC | 1.50556 | 1.405512 | 43617453 | 0.921278 | 0 | 0 | 89.24545 | 7.420486 |
| DPYD | CTC | 2.472066 | 1.40048 | 38325291 | 0.809499 | 6.74E-305 | 3.66E-303 | 93.92888 | 56.88715 |
| DOCK8 | CTC | 1.793154 | 1.37343 | 40945562 | 0.864843 | 0 | 0 | 92.28101 | 26.93975 |
| LST1 | CTC | 1.496166 | 1.345708 | 43166872 | 0.911761 | 0 | 0 | 90.45967 | 10.80561 |
| LSP1 | CTC | 1.587569 | 1.343567 | 41924380 | 0.885518 | 0 | 0 | 90.28621 | 15.37188 |
| S100A12 | CTC | 1.395607 | 1.330096 | 41671941 | 0.880186 | 0 | 0 | 79.87858 | 4.661244 |
| ARPC1B | CTC | 2.229547 | 1.298076 | 41685599 | 0.880474 | 0 | 0 | 94.79618 | 65.78345 |
| COTL1 | CTC | 1.844999 | 1.293257 | 40136186 | 0.847748 | 0 | 0 | 92.97485 | 37.92801 |
| CD52 | CTC | 1.582121 | 1.292927 | 39409864 | 0.832407 | 0 | 0 | 81.43972 | 15.48634 |
| AOAH | CTC | 1.49839 | 1.291365 | 40695425 | 0.85956 | 0 | 0 | 82.74068 | 13.91067 |
| SAMHD1 | CTC | 1.472593 | 1.244396 | 41699443 | 0.880767 | 0 | 0 | 87.94449 | 19.01515 |
| CLEC12A | CTC | 1.29952 | 1.232454 | 42799780 | 0.904008 | 0 | 0 | 84.90893 | 4.98758 |
| SLC8A1 | CTC | 1.588174 | 1.228846 | 38620023 | 0.815724 | 0 | 0 | 80.83261 | 20.30831 |
| MYO1F | CTC | 1.380779 | 1.226963 | 42641870 | 0.900672 | 0 | 0 | 89.24545 | 11.22449 |
| EVI2B | CTC | 1.441732 | 1.21977 | 41605198 | 0.878776 | 0 | 0 | 88.29141 | 15.36457 |
| LGALS1 | CTC | 2.089473 | 1.218278 | 38092771 | 0.804587 | 5.84E-303 | 3.14E-301 | 92.97485 | 52.80064 |
| PRKCB | CTC | 1.457592 | 1.21511 | 41227612 | 0.870801 | 0 | 0 | 88.29141 | 15.66168 |
| GABARAP | CTC | 2.363777 | 1.215045 | 40963817 | 0.865229 | 0 | 0 | 97.39809 | 72.7193 |
| IGSF6 | CTC | 1.381588 | 1.198831 | 40699223 | 0.85964 | 0 | 0 | 82.82741 | 11.78949 |
| TBXAS1 | CTC | 1.36988 | 1.189795 | 41268040 | 0.871655 | 0 | 0 | 84.90893 | 12.64429 |
| IQGAP2 | CTC | 1.511583 | 1.185994 | 40443827 | 0.854246 | 0 | 0 | 89.59237 | 19.93814 |
| FYB1 | CTC | 1.596425 | 1.176179 | 39404679 | 0.832297 | 0 | 0 | 89.07199 | 25.74887 |
| SPI1 | CTC | 1.324847 | 1.164534 | 42376016 | 0.895057 | 0 | 0 | 89.33218 | 11.33652 |
| EMP3 | CTC | 1.476144 | 1.128974 | 40426325 | 0.853876 | 0 | 0 | 89.85256 | 26.53792 |
| PLEK | CTC | 1.484606 | 1.120296 | 39542304 | 0.835204 | 0 | 0 | 89.24545 | 19.83586 |
| CD36 | CTC | 1.18929 | 1.119818 | 40610014 | 0.857756 | 0 | 0 | 75.88899 | 5.408894 |
| ATG7 | CTC | 1.4872 | 1.119553 | 40794721 | 0.861657 | 0 | 0 | 89.76583 | 34.27743 |
| MARCH1 | CTC | 1.378028 | 1.112748 | 39266702 | 0.829383 | 0 | 0 | 80.05204 | 24.17564 |
| RNF130 | CTC | 1.644428 | 1.108969 | 40853459 | 0.862898 | 0 | 0 | 91.15351 | 48.47791 |
| TSPO | CTC | 2.205189 | 1.103691 | 39468854 | 0.833653 | 0 | 0 | 95.66349 | 67.29336 |
| RNASEK | CTC | 1.92719 | 1.102702 | 40038404 | 0.845683 | 0 | 0 | 94.70945 | 63.80839 |
| AP1S2 | CTC | 1.274368 | 1.088486 | 41443509 | 0.875361 | 0 | 0 | 86.64354 | 16.99625 |
| ARHGAP24 | CTC | 1.420435 | 1.079413 | 38227643 | 0.807436 | 0 | 0 | 80.39896 | 30.09595 |
| ARHGAP26 | CTC | 1.697374 | 1.077713 | 38283098 | 0.808607 | 0 | 0 | 89.07199 | 46.71472 |
| IRAK3 | CTC | 1.272196 | 1.076125 | 40594444 | 0.857427 | 0 | 0 | 83.43452 | 17.81696 |
| FGL2 | CTC | 1.196271 | 1.069149 | 39767239 | 0.839955 | 0 | 0 | 75.62879 | 9.480785 |
| ARHGAP15 | CTC | 1.6861 | 1.065896 | 36976135 | 0.781002 | 0 | 0 | 89.24545 | 31.53524 |
| SYK | CTC | 1.300356 | 1.056537 | 40943189 | 0.864793 | 0 | 0 | 86.817 | 25.52725 |
| SMAP2 | CTC | 1.506455 | 1.056332 | 39062430 | 0.825068 | 0 | 0 | 87.07719 | 37.86713 |
| KYNU | CTC | 1.402592 | 1.053691 | 38578650 | 0.81485 | 0 | 0 | 83.0876 | 22.24441 |
| HDAC9 | CTC | 1.498345 | 1.041021 | 36323396 | 0.767215 | 8.68E-288 | 4.36E-286 | 77.18994 | 34.28474 |
| RTN1 | CTC | 1.085531 | 1.033018 | 40992462 | 0.865834 | 0 | 0 | 76.75629 | 4.639326 |
| SSH2 | CTC | 1.640497 | 1.024986 | 38923398 | 0.822132 | 0 | 0 | 90.98005 | 52.06273 |
| BACH1 | CTC | 1.554337 | 1.022131 | 39113357 | 0.826144 | 0 | 0 | 88.5516 | 46.66845 |
| DOCK2 | CTC | 1.297194 | 1.019526 | 39678343 | 0.838077 | 0 | 0 | 85.51605 | 18.28698 |
| GLIPR1 | CTC | 1.256494 | 1.011806 | 40286541 | 0.850924 | 0 | 0 | 84.90893 | 21.84989 |
| USP15 | CTC | 1.643642 | 1.004573 | 37717903 | 0.796669 | 3.67E-285 | 1.81E-283 | 89.15872 | 54.15469 |
| GMFG | CTC | 1.286578 | 1.002052 | 40618308 | 0.857931 | 0 | 0 | 91.67389 | 19.7287 |

### Table S3. The mRNA expression of CTCs.

|  | KRT8 | KRT18 | EPCAM | NRG1 | ZEB2 |  |  | KRT8 | KRT18 | EPCAM | NRG1 | ZEB2 |
| --- | --- | --- | --- | --- | --- | --- | --- | --- | --- | --- | --- | --- |
| H41_CTC_AAACGTAAGGATGACG | 0 | 0 | 0 | 0 | 12 |  | H42_CTC_GGCGTAGGTTAGGTTC | 1 | 1 | 0 | 0 | 26 |
| H41_CTC_AAACGTCCAAGCGTGC | 1 | 0 | 0 | 0 | 29 |  | H42_CTC_GGCGTTACATCCCAGT | 0 | 0 | 0 | 0 | 47 |
| H41_CTC_AAACTTGCAGGACTAT | 0 | 0 | 0 | 0 | 9 |  | H42_CTC_GGCTTCCTCAGCCCGA | 0 | 1 | 0 | 0 | 11 |
| H41_CTC_AAAGCCAGTTTGGAAG | 0 | 0 | 0 | 1 | 2 |  | H42_CTC_GGCTTCTCACTATACT | 1 | 4 | 0 | 0 | 91 |
| H41_CTC_AACAGCTCACATACTT | 0 | 1 | 0 | 0 | 1 |  | H42_CTC_GGCTTGGTCGCTGAGT | 0 | 0 | 0 | 0 | 1 |
| H41_CTC_AACATAGAGATTCACG | 0 | 0 | 0 | 4 | 19 |  | H42_CTC_GGGACAATCCGTAGGT | 0 | 0 | 0 | 5 | 37 |
| H41_CTC_AACCGAAGTTATCAGC | 0 | 1 | 0 | 0 | 61 |  | H42_CTC_GGGACCAGTTTCGGAT | 0 | 0 | 0 | 0 | 3 |
| H41_CTC_AACGAATGTGATAGAT | 0 | 0 | 0 | 0 | 0 |  | H42_CTC_GGGACCTCATTAGCTT | 2 | 2 | 0 | 0 | 19 |
| H41_CTC_AACGATTTCCGGATAC | 0 | 1 | 0 | 1 | 30 |  | H42_CTC_GGGATATAGCATGCCG | 1 | 0 | 0 | 0 | 11 |
| H41_CTC_AACGCCCGTCTAGCGC | 2 | 0 | 0 | 0 | 11 |  | H42_CTC_GGGATTGAGTGATCGC | 0 | 0 | 0 | 0 | 2 |
| H41_CTC_AACGCTTGTTTCCGTC | 0 | 1 | 0 | 2 | 21 |  | H42_CTC_GGGCAATGTAGCTAGA | 1 | 0 | 0 | 0 | 18 |
| H41_CTC_AACGGTAGTGCGCGTT | 0 | 1 | 0 | 0 | 8 |  | H42_CTC_GGGCATGGTTGTCACG | 1 | 0 | 0 | 0 | 17 |
| H41_CTC_AACTGACCATTACTAC | 0 | 0 | 0 | 0 | 9 |  | H42_CTC_GGGTGTCCAGTTAAGC | 1 | 0 | 0 | 0 | 8 |
| H41_CTC_AACTTGGTCGCTCGTC | 1 | 0 | 0 | 0 | 17 |  | H42_CTC_GGGTTCCGTCTGGCGT | 1 | 0 | 0 | 0 | 8 |
| H41_CTC_AAGTATGAGTGCAAGG | 0 | 0 | 0 | 0 | 2 |  | H42_CTC_GGGTTGGGTTAGCAAG | 1 | 1 | 0 | 0 | 25 |
| H41_CTC_AAGTTGGGTTTAAGCC | 0 | 0 | 0 | 8 | 10 |  | H42_CTC_GGGTTTGCAATCTGCC | 0 | 1 | 0 | 0 | 48 |
| H41_CTC_AATATCGCATTAAGTG | 1 | 0 | 0 | 1 | 5 |  | H42_CTC_GGTACGGGTTAACCGT | 0 | 1 | 0 | 0 | 25 |
| H41_CTC_AATATGCTCCCCAAGG | 0 | 0 | 0 | 1 | 30 |  | H42_CTC_GGTAGCGGTTATGGCT | 2 | 0 | 0 | 0 | 6 |
| H41_CTC_AATATGCTCGCGTCTC | 0 | 1 | 0 | 1 | 35 |  | H42_CTC_GGTCACTAGCCAGTTA | 2 | 0 | 0 | 1 | 24 |
| H41_CTC_AATCAGCCAACGTTCC | 1 | 0 | 0 | 1 | 14 |  | H42_CTC_GGTCACTAGTTGCATG | 0 | 0 | 0 | 0 | 41 |
| H41_CTC_AATCGCATCCGGGTCA | 0 | 2 | 0 | 0 | 4 |  | H42_CTC_GGTCGCATCGACCTAA | 1 | 2 | 0 | 0 | 0 |
| H41_CTC_AATGACCCATAGGTCG | 0 | 0 | 0 | 2 | 26 |  | H42_CTC_GGTGAACGTAGGTCGG | 0 | 1 | 0 | 0 | 26 |
| H41_CTC_ACAAGGTAGGATGAAC | 0 | 0 | 0 | 0 | 18 |  | H42_CTC_GGTGGTAAGTAGCCAC | 1 | 0 | 0 | 0 | 20 |
| H41_CTC_ACACGGTTCAGTTGGC | 0 | 0 | 0 | 0 | 7 |  | H42_CTC_GTAAGGCGTTAGGTTC | 1 | 0 | 0 | 0 | 6 |
| H41_CTC_ACAGCTATCACCTAAG | 1 | 0 | 0 | 5 | 14 |  | H42_CTC_GTAAGTAAGATTACGT | 1 | 0 | 0 | 1 | 34 |
| H41_CTC_ACAGCTCAGGCGATAG | 0 | 0 | 0 | 0 | 31 |  | H42_CTC_GTAATTCAGCGATGGA | 0 | 1 | 0 | 1 | 7 |
| H41_CTC_ACAGCTCAGTCAATCG | 0 | 1 | 0 | 0 | 15 |  | H42_CTC_GTAATTCAGGATGAAC | 1 | 1 | 0 | 0 | 31 |
| H41_CTC_ACCACACAGCATAGTT | 1 | 0 | 0 | 3 | 24 |  | H42_CTC_GTACGATCAAGGTGAA | 1 | 0 | 0 | 0 | 34 |
| H41_CTC_ACCCAATGTAGCGCCT | 0 | 0 | 0 | 0 | 15 |  | H42_CTC_GTACGGTTCAATCACG | 0 | 0 | 0 | 3 | 63 |
| H41_CTC_ACCCGATAGCGAATCC | 0 | 1 | 0 | 2 | 33 |  | H42_CTC_GTAGGAACAATGGAAG | 0 | 0 | 0 | 0 | 24 |
| H41_CTC_ACCCGCTTCCGTGCAG | 0 | 0 | 0 | 0 | 31 |  | H42_CTC_GTATGGCTCGTCTATC | 0 | 0 | 0 | 0 | 7 |
| H41_CTC_ACCTGAATCAGGTCAC | 0 | 0 | 0 | 4 | 0 |  | H42_CTC_GTCATAGTCTATGGGC | 0 | 1 | 0 | 0 | 2 |
| H41_CTC_ACGCAGCCACAAGGCC | 0 | 0 | 0 | 0 | 4 |  | H42_CTC_GTCATCAAGGTCACGT | 0 | 0 | 0 | 0 | 7 |
| H41_CTC_ACGGCAGGTGAAGTTG | 1 | 0 | 0 | 2 | 32 |  | H42_CTC_GTCATCAAGTCAACCA | 0 | 1 | 0 | 0 | 18 |
| H41_CTC_ACGGCCATCCCTGGCT | 0 | 0 | 0 | 0 | 8 |  | H42_CTC_GTCCAGGTCGTAATCG | 0 | 1 | 0 | 0 | 8 |
| H41_CTC_ACGGCTTAGGTTACAT | 0 | 0 | 0 | 1 | 19 |  | H42_CTC_GTCCATTTCGTAATCG | 0 | 2 | 0 | 0 | 28 |
| H41_CTC_ACGGGTTTCACTTGAG | 0 | 0 | 0 | 0 | 7 |  | H42_CTC_GTCCCATCATCGTAGG | 1 | 0 | 0 | 0 | 8 |
| H41_CTC_ACGTTTAGTAGGTGTA | 0 | 0 | 0 | 0 | 1 |  | H42_CTC_GTCGGATTCTAAGCAT | 1 | 1 | 0 | 0 | 14 |
| H41_CTC_ACTCCAAAGTGAGAGT | 0 | 1 | 0 | 0 | 2 |  | H42_CTC_GTGAATGGTTTAATCG | 1 | 0 | 0 | 0 | 13 |
| H41_CTC_ACTCGTTTCACTCCTG | 1 | 0 | 0 | 0 | 19 |  | H42_CTC_GTGCACTAGGTAGTCC | 0 | 0 | 0 | 0 | 4 |
| H41_CTC_ACTGTTGAGCTATGAT | 1 | 0 | 0 | 0 | 2 |  | H42_CTC_GTGCCCAAGTGAGGTG | 0 | 2 | 0 | 0 | 0 |
| H41_CTC_AGACTGCTCCTGAGTA | 0 | 0 | 0 | 1 | 19 |  | H42_CTC_GTGCTATGTGAGACCG | 1 | 0 | 0 | 0 | 13 |
| H41_CTC_AGAGAATCAACCAACG | 0 | 0 | 1 | 0 | 1 |  | H42_CTC_GTGGCCATCCTTCCTC | 1 | 1 | 0 | 0 | 18 |
| H41_CTC_AGAGACCGTCATGATC | 0 | 0 | 0 | 3 | 35 |  | H42_CTC_GTGGCGAGTGATGAGC | 0 | 0 | 0 | 2 | 31 |
| H41_CTC_AGAGATGCAGGTTCGA | 0 | 1 | 0 | 0 | 40 |  | H42_CTC_GTGGTAAGTTTGACCG | 1 | 0 | 0 | 0 | 9 |
| H41_CTC_AGAGGTTAGTTGCACA | 0 | 1 | 0 | 0 | 14 |  | H42_CTC_GTGTGTTGTGGCAGCA | 0 | 0 | 1 | 0 | 1 |
| H41_CTC_AGCATGAGTCTTGGGC | 0 | 0 | 0 | 0 | 31 |  | H42_CTC_GTTACCCCAACTGGGC | 0 | 2 | 0 | 1 | 9 |
| H41_CTC_AGCCTCCTCCAACTGA | 1 | 0 | 0 | 0 | 7 |  | H42_CTC_GTTAGTTAGGCAATCC | 0 | 0 | 0 | 0 | 33 |
| H41_CTC_AGCCTTGGTTCCGCAA | 0 | 1 | 0 | 0 | 13 |  | H42_CTC_GTTATCAGTTCGCTAG | 1 | 1 | 0 | 1 | 22 |
| H41_CTC_AGCGTTATCCGCGCTT | 1 | 0 | 0 | 13 | 24 |  | H42_CTC_GTTATGGTCGAACCAC | 0 | 0 | 0 | 0 | 34 |
| H41_CTC_AGGAATGCAAGCCGTA | 0 | 0 | 0 | 0 | 4 |  | H42_CTC_GTTGAAGGTAAGTGAT | 1 | 1 | 0 | 0 | 22 |
| H41_CTC_AGGAGATTCATATAGC | 0 | 1 | 0 | 0 | 16 |  | H42_CTC_TAAATGCTCTATGTGG | 0 | 2 | 0 | 0 | 1 |
| H41_CTC_AGGAGTTAGCGCTCTC | 0 | 0 | 1 | 0 | 12 |  | H42_CTC_TAACGCGCACAACTAA | 0 | 0 | 0 | 0 | 12 |
| H41_CTC_AGGAGTTAGTCTCTGT | 0 | 0 | 0 | 1 | 18 |  | H42_CTC_TAACGGCTCGTAACCA | 0 | 0 | 0 | 0 | 16 |
| H41_CTC_AGGCAATAGCATGTGC | 1 | 1 | 0 | 1 | 32 |  | H42_CTC_TACACAACAACTACTT | 0 | 1 | 0 | 0 | 19 |
| H41_CTC_AGGCATTCAAGCCTAG | 0 | 1 | 0 | 2 | 47 |  | H42_CTC_TACACAACAAGCGACC | 0 | 0 | 0 | 0 | 26 |
| H41_CTC_AGGGATTAGGCAACCT | 0 | 1 | 0 | 18 | 60 |  | H42_CTC_TACCGACAGCATATCG | 0 | 1 | 0 | 1 | 29 |
| H41_CTC_AGGGTTAGTCAACTCG | 0 | 0 | 1 | 0 | 2 |  | H42_CTC_TACCGACAGTATGGAA | 0 | 0 | 0 | 0 | 13 |
| H41_CTC_AGGTGATAGTAACCTG | 1 | 0 | 0 | 3 | 10 |  | H42_CTC_TACCTAAGTAAGGTTG | 0 | 3 | 0 | 0 | 22 |
| H41_CTC_AGGTTCGCATTAAGTG | 0 | 0 | 1 | 0 | 3 |  | H42_CTC_TACGCTGGTTCGTTGG | 1 | 1 | 0 | 0 | 15 |
| H41_CTC_AGTATCACAAATTCGA | 0 | 1 | 0 | 0 | 1 |  | H42_CTC_TACTGCAAGGATCCCG | 0 | 0 | 0 | 0 | 13 |
| H41_CTC_AGTATCACATGCTCGT | 0 | 0 | 0 | 8 | 16 |  | H42_CTC_TAGCTCCCACTTGATT | 0 | 0 | 1 | 0 | 1 |
| H41_CTC_AGTATGGGTAATTGCC | 0 | 0 | 0 | 2 | 13 |  | H42_CTC_TAGGTCATCCATTGCT | 0 | 1 | 0 | 12 | 40 |
| H41_CTC_AGTGGATAGGTGCGTT | 0 | 0 | 0 | 0 | 3 |  | H42_CTC_TATAGGCCAAACCGAT | 0 | 0 | 0 | 0 | 18 |
| H41_CTC_ATAACCACATCCACGT | 1 | 0 | 0 | 0 | 52 |  | H42_CTC_TATAGGCCACAGCCTT | 0 | 0 | 0 | 0 | 2 |
| H41_CTC_ATACGTTAGAAGGAGA | 0 | 3 | 0 | 4 | 12 |  | H42_CTC_TATAGTCAGCATGGCT | 0 | 0 | 0 | 0 | 29 |
| H41_CTC_ATACTTGGTGGCGTGA | 0 | 0 | 0 | 1 | 17 |  | H42_CTC_TATCCGTTCATGAGTT | 1 | 0 | 0 | 0 | 42 |
| H41_CTC_ATAGCGGAGGTCAATT | 0 | 0 | 2 | 0 | 6 |  | H42_CTC_TATCGTAGTTGTAGTC | 0 | 0 | 0 | 0 | 2 |
| H41_CTC_ATCACGATCGCTAGGT | 0 | 1 | 0 | 4 | 14 |  | H42_CTC_TATCGTTCATGAGTTA | 0 | 0 | 0 | 0 | 13 |
| H41_CTC_ATCCCAGGTTCCGTGA | 0 | 0 | 1 | 8 | 30 |  | H42_CTC_TCAAACTAGTTGGATT | 0 | 0 | 0 | 1 | 11 |
| H41_CTC_ATCTTCATCTGGTGTA | 0 | 0 | 0 | 0 | 38 |  | H42_CTC_TCACACAAGGATTAAG | 0 | 0 | 0 | 0 | 33 |
| H41_CTC_ATGCAGGAGGCGTCAT | 0 | 0 | 0 | 7 | 27 |  | H42_CTC_TCACATAGTGAGTGCG | 1 | 0 | 0 | 0 | 42 |
| H41_CTC_ATGTACCGTGGAAGCT | 1 | 0 | 0 | 0 | 26 |  | H42_CTC_TCACCCGCAAAGGCAA | 1 | 0 | 0 | 0 | 0 |
| H41_CTC_ATGTTCTCATCCGCTG | 0 | 0 | 0 | 1 | 46 |  | H42_CTC_TCACGGGTCGCTTCGC | 1 | 0 | 0 | 0 | 10 |
| H41_CTC_ATTACAGCAAGCTGGC | 1 | 0 | 0 | 0 | 57 |  | H42_CTC_TCAGCTGGTTAGCTTA | 1 | 0 | 0 | 0 | 44 |
| H41_CTC_ATTCGGGCAGCCAGGT | 0 | 0 | 0 | 1 | 20 |  | H42_CTC_TCAGTTGAGCGAACCT | 0 | 1 | 0 | 0 | 14 |
| H41_CTC_ATTGGCCAGGGGATGC | 0 | 0 | 0 | 0 | 5 |  | H42_CTC_TCATCGAAGGTTAGTT | 0 | 1 | 0 | 0 | 13 |
| H41_CTC_CAAATGTTCACTCTGT | 1 | 0 | 0 | 0 | 29 |  | H42_CTC_TCCAATGAGGCTACAC | 0 | 1 | 0 | 1 | 9 |
| H41_CTC_CAACTAACATAACCGG | 0 | 0 | 0 | 1 | 9 |  | H42_CTC_TCCCACGCAGGATCAT | 0 | 0 | 0 | 14 | 39 |
| H41_CTC_CAAGACAGTGGAGCTG | 0 | 0 | 1 | 4 | 56 |  | H42_CTC_TCCCGATCAACCAGCA | 0 | 0 | 0 | 1 | 5 |
| H41_CTC_CAAGCCACATGACGGC | 1 | 0 | 0 | 36 | 46 |  | H42_CTC_TCCTAGAAGGCGTATC | 0 | 0 | 0 | 0 | 27 |
| H41_CTC_CAAGGTTCAAGGGTCC | 0 | 0 | 0 | 0 | 26 |  | H42_CTC_TCCTCGATCCTCGATT | 1 | 0 | 0 | 0 | 37 |
| H41_CTC_CACGATGCATCGATCC | 0 | 1 | 0 | 4 | 45 |  | H42_CTC_TCCTCTTCACTCAACC | 0 | 0 | 0 | 0 | 10 |
| H41_CTC_CACTACTTCCCTGTCA | 0 | 0 | 0 | 0 | 28 |  | H42_CTC_TCCTGGTCATTGGAGT | 0 | 2 | 0 | 0 | 2 |
| H41_CTC_CACTCGCTCCATCATC | 0 | 1 | 0 | 2 | 51 |  | H42_CTC_TCCTGTTAGGCAACTC | 1 | 1 | 0 | 0 | 17 |
| H41_CTC_CACTGCCTCGTTGCTT | 1 | 0 | 0 | 4 | 46 |  | H42_CTC_TCCTTCTCACTAGGTG | 0 | 0 | 0 | 0 | 4 |
| H41_CTC_CAGCTAATCGTTGCGG | 0 | 2 | 0 | 0 | 42 |  | H42_CTC_TCGACGTAGGGCTAGA | 0 | 0 | 0 | 1 | 13 |
| H41_CTC_CAGGACCCAGGCATTG | 0 | 1 | 0 | 0 | 30 |  | H42_CTC_TCGCAACAGGGTGATG | 2 | 0 | 0 | 1 | 7 |
| H41_CTC_CAGGCACCAGGCAACC | 0 | 0 | 0 | 1 | 19 |  | H42_CTC_TCGCATAAGTACGGCT | 0 | 0 | 0 | 0 | 20 |
| H41_CTC_CAGGTTATCAGCGATC | 0 | 0 | 0 | 3 | 11 |  | H42_CTC_TCGGGGTCACTTGACC | 0 | 0 | 0 | 1 | 14 |
| H41_CTC_CAGTAGGAGGTATAGA | 1 | 0 | 0 | 13 | 15 |  | H42_CTC_TCGGTCAGTAGCTAGA | 1 | 0 | 0 | 0 | 22 |
| H41_CTC_CAGTCTAAGAATCCAT | 0 | 0 | 0 | 1 | 6 |  | H42_CTC_TCGTCACCATGACGAT | 1 | 0 | 0 | 0 | 11 |
| H41_CTC_CAGTTATAGGAATGAC | 0 | 1 | 0 | 0 | 21 |  | H42_CTC_TCGTTAGAGCGAGACA | 0 | 0 | 0 | 1 | 1 |
| H41_CTC_CAGTTCAAGATTAAGG | 1 | 0 | 0 | 1 | 6 |  | H42_CTC_TCTAAGGTCATCAGTG | 0 | 1 | 0 | 0 | 11 |
| H41_CTC_CATGTCAAGGGCAGAC | 0 | 1 | 0 | 0 | 18 |  | H42_CTC_TCTACGGGTGAGACCG | 0 | 1 | 0 | 0 | 13 |
| H41_CTC_CATTAGGTCCGTCCGT | 0 | 0 | 1 | 0 | 5 |  | H42_CTC_TCTACGGGTTTAGGCT | 0 | 0 | 0 | 0 | 11 |
| H41_CTC_CCAACCTCAAGTGCCA | 0 | 0 | 0 | 0 | 17 |  | H42_CTC_TCTCATATCGACCGTG | 0 | 1 | 0 | 0 | 7 |
| H41_CTC_CCACGTAGTATGGCAA | 0 | 0 | 0 | 0 | 6 |  | H42_CTC_TCTCCATAGTTACGGC | 0 | 0 | 0 | 0 | 26 |
| H41_CTC_CCACTCGCAACCAACG | 0 | 1 | 0 | 3 | 30 |  | H42_CTC_TCTCTATGTGGCTACC | 0 | 0 | 0 | 1 | 8 |
| H41_CTC_CCAGCAACAACGGTCA | 0 | 1 | 0 | 0 | 38 |  | H42_CTC_TCTGGGACACTTGGGA | 0 | 3 | 0 | 0 | 39 |
| H41_CTC_CCAGGCATCACCTGAA | 0 | 0 | 0 | 4 | 32 |  | H42_CTC_TGAAATGAGGTAGTAA | 0 | 1 | 0 | 1 | 19 |
| H41_CTC_CCCATGGAGCGAGTTC | 0 | 0 | 0 | 1 | 12 |  | H42_CTC_TGAAATGAGTGAGTGG | 1 | 0 | 0 | 0 | 31 |
| H41_CTC_CCCGTTGCATTAGGGT | 0 | 0 | 0 | 3 | 9 |  | H42_CTC_TGAACCGCATGGTTGG | 2 | 1 | 0 | 0 | 37 |
| H41_CTC_CCCTCACAGAAGGACT | 0 | 0 | 0 | 3 | 30 |  | H42_CTC_TGAATGAAGGTAGTCC | 1 | 0 | 0 | 0 | 7 |
| H41_CTC_CCCTCACAGGGGTAGC | 0 | 0 | 0 | 0 | 6 |  | H42_CTC_TGAATGTTCGATACGC | 0 | 0 | 0 | 0 | 10 |
| H41_CTC_CCCTTTAGTCTGTGTG | 0 | 0 | 0 | 4 | 27 |  | H42_CTC_TGACTAACAATTCATC | 0 | 0 | 0 | 0 | 9 |
| H41_CTC_CCGAATATCGATACTG | 1 | 0 | 0 | 0 | 32 |  | H42_CTC_TGACTCCCATTCATCG | 0 | 0 | 0 | 1 | 7 |
| H41_CTC_CCGAATTGTCTGGGCT | 0 | 0 | 0 | 3 | 27 |  | H42_CTC_TGAGCATCAGGCTTGA | 0 | 0 | 0 | 1 | 23 |
| H41_CTC_CCGACCAAGGTAGGCA | 0 | 0 | 0 | 1 | 5 |  | H42_CTC_TGAGCGAAGCAAGGTG | 0 | 1 | 0 | 0 | 21 |
| H41_CTC_CCGCTTGCAGTTGGAT | 1 | 1 | 0 | 0 | 3 |  | H42_CTC_TGAGGCGGTTCGGCGT | 0 | 0 | 0 | 0 | 12 |
| H41_CTC_CCGGCTAAGTCATTCC | 0 | 1 | 0 | 2 | 20 |  | H42_CTC_TGAGTCTGTTAGCCCA | 0 | 0 | 0 | 0 | 3 |
| H41_CTC_CCGGTACGTTCCCAGA | 0 | 0 | 0 | 0 | 8 |  | H42_CTC_TGATCCGGTTGTCTGG | 0 | 0 | 0 | 0 | 15 |
| H41_CTC_CCGGTATAGTAACCCA | 0 | 0 | 0 | 2 | 22 |  | H42_CTC_TGATGAGGTAATGGCA | 1 | 0 | 0 | 0 | 4 |
| H41_CTC_CCGTATGGTCTTGAGT | 1 | 0 | 0 | 0 | 10 |  | H42_CTC_TGCCACCTCTAATGCG | 0 | 0 | 0 | 0 | 48 |
| H41_CTC_CCGTTAGGTGGCGACA | 0 | 1 | 0 | 0 | 11 |  | H42_CTC_TGCCCAAGTCCATTCC | 0 | 0 | 0 | 0 | 11 |
| H41_CTC_CCTATTATCGAATTAC | 0 | 0 | 0 | 0 | 32 |  | H42_CTC_TGCCCATCAAGTGTCG | 2 | 0 | 0 | 0 | 14 |
| H41_CTC_CCTCAATTCACTCGGG | 1 | 0 | 0 | 8 | 36 |  | H42_CTC_TGCCGTGAGGGTGAGC | 1 | 0 | 0 | 0 | 30 |
| H41_CTC_CCTCCTGGTGCGCAAG | 0 | 1 | 0 | 0 | 13 |  | H42_CTC_TGCCGTGAGGTACATT | 0 | 0 | 0 | 3 | 18 |
| H41_CTC_CCTCGGTTCCATCTAC | 0 | 1 | 0 | 2 | 26 |  | H42_CTC_TGCCTGCTCGAATTAC | 0 | 0 | 0 | 0 | 7 |
| H41_CTC_CCTGCGAAGTAGCATT | 0 | 1 | 0 | 13 | 31 |  | H42_CTC_TGCGGCTGTTAATCTG | 1 | 1 | 0 | 0 | 2 |
| H41_CTC_CCTTTAATCGCTATCT | 1 | 0 | 0 | 0 | 1 |  | H42_CTC_TGCTCGCTCCAGCGTG | 0 | 1 | 0 | 1 | 85 |
| H41_CTC_CGATCACAGAAGGTGT | 0 | 1 | 0 | 2 | 44 |  | H42_CTC_TGCTCGCTCTAAGCGC | 0 | 1 | 0 | 0 | 25 |
| H41_CTC_CGATGGGGTTAGCCCA | 0 | 0 | 0 | 0 | 8 |  | H42_CTC_TGGCATATCGACATTG | 0 | 0 | 0 | 0 | 2 |
| H41_CTC_CGCACAACAACTTCAT | 0 | 2 | 0 | 1 | 31 |  | H42_CTC_TGGCTGACATTCGCCG | 1 | 1 | 0 | 0 | 10 |
| H41_CTC_CGCACATAGGTTCGCG | 0 | 0 | 0 | 1 | 24 |  | H42_CTC_TGGGATTCAGTTGGCA | 0 | 1 | 0 | 3 | 21 |
| H41_CTC_CGCAGATTCAATCGCA | 1 | 0 | 0 | 2 | 14 |  | H42_CTC_TGGTCACAGCTTACGA | 0 | 0 | 0 | 10 | 78 |
| H41_CTC_CGCAGTTAGGATTGAA | 0 | 0 | 0 | 0 | 11 |  | H42_CTC_TGGTGTTGTTAGCGTT | 1 | 0 | 0 | 0 | 21 |
| H41_CTC_CGCCATTCACTCATCA | 1 | 0 | 0 | 0 | 20 |  | H42_CTC_TGGTTATAGGGCTAGA | 0 | 0 | 0 | 0 | 11 |
| H41_CTC_CGCCTCACAATGACAC | 0 | 0 | 0 | 1 | 24 |  | H42_CTC_TGTAACCGTTCGCTGA | 0 | 1 | 0 | 0 | 0 |
| H41_CTC_CGCGAAGGTGAGCCGA | 1 | 0 | 0 | 8 | 13 |  | H42_CTC_TGTAGGTCAGGCAAGG | 1 | 1 | 0 | 0 | 38 |
| H41_CTC_CGCTGATAGGAGCTGA | 0 | 3 | 2 | 1 | 25 |  | H42_CTC_TGTATAGCACCTCACG | 0 | 7 | 0 | 0 | 70 |
| H41_CTC_CGGCTGATCACATGAT | 0 | 0 | 0 | 0 | 10 |  | H42_CTC_TGTCACCCACAACCCT | 0 | 0 | 0 | 0 | 41 |
| H41_CTC_CGGGACTCAATTGGTA | 0 | 1 | 0 | 0 | 18 |  | H42_CTC_TGTCCCATCCCGTAGG | 0 | 1 | 0 | 0 | 4 |
| H41_CTC_CGGGACTCACAACTAA | 0 | 0 | 0 | 0 | 10 |  | H42_CTC_TGTCCCGCAAAGGGTA | 0 | 1 | 0 | 0 | 35 |
| H41_CTC_CGGGGTAGTTTACTGA | 1 | 0 | 0 | 1 | 20 |  | H42_CTC_TGTGCATGTGCAGGAT | 1 | 0 | 0 | 0 | 38 |
| H41_CTC_CGGTTAGCATTCGCAC | 0 | 1 | 0 | 7 | 11 |  | H25_CTC_AAACGAATCGTTCATT | 0 | 0 | 0 | 6 | 115 |
| H41_CTC_CGTACTAAGTAACCCA | 0 | 0 | 0 | 0 | 6 |  | H25_CTC_AAACGCTTCAAACTGC | 0 | 0 | 0 | 16 | 68 |
| H41_CTC_CGTAGGTTCCCCATGC | 0 | 1 | 0 | 0 | 11 |  | H25_CTC_AAAGTCCGTACATACC | 0 | 0 | 0 | 0 | 94 |
| H41_CTC_CGTATGATCCATCGTT | 2 | 0 | 0 | 0 | 21 |  | H25_CTC_AAATGGAAGTATCGCC | 0 | 0 | 0 | 0 | 4 |
| H41_CTC_CGTATGATCTCATTCA | 0 | 0 | 0 | 12 | 16 |  | H25_CTC_AACAAAGAGAGTAACT | 0 | 0 | 0 | 0 | 72 |
| H41_CTC_CGTGCCTTCGCTCACC | 1 | 0 | 0 | 6 | 33 |  | H25_CTC_AACCAACTCTGGACCG | 0 | 0 | 0 | 0 | 0 |
| H41_CTC_CGTGCTAGTTTGGAAG | 0 | 0 | 1 | 0 | 13 |  | H25_CTC_AACCTGAAGACTTAAG | 0 | 0 | 0 | 3 | 80 |
| H41_CTC_CGTTGCCTCCGCATGG | 0 | 2 | 0 | 0 | 4 |  | H25_CTC_AACCTGAAGTGAATAC | 0 | 0 | 0 | 0 | 0 |
| H41_CTC_CTAAGGTCAGGAAGCG | 1 | 0 | 0 | 0 | 5 |  | H25_CTC_AACGGGACAACTAGAA | 0 | 0 | 0 | 0 | 73 |
| H41_CTC_CTACCATTCACGTAGC | 1 | 0 | 0 | 1 | 12 |  | H25_CTC_AACGGGAGTTTACGAC | 0 | 0 | 0 | 17 | 31 |
| H41_CTC_CTACCCTGTTGCATCG | 0 | 0 | 0 | 1 | 16 |  | H25_CTC_AAGAACATCACCCATC | 0 | 0 | 0 | 0 | 7 |
| H41_CTC_CTAGGTTGTTTACTAG | 1 | 0 | 0 | 0 | 3 |  | H25_CTC_AAGAACATCCTCTGCA | 0 | 0 | 0 | 0 | 69 |
| H41_CTC_CTATAGGTCAAGTGCC | 2 | 0 | 0 | 0 | 16 |  | H25_CTC_AAGATAGTCCTGTACC | 0 | 0 | 0 | 1 | 21 |
| H41_CTC_CTCACCATCGAATCAT | 0 | 0 | 0 | 7 | 14 |  | H25_CTC_AAGTCGTAGCACTTTG | 0 | 0 | 0 | 0 | 0 |
| H41_CTC_CTCATTATCGATGCGT | 0 | 0 | 0 | 3 | 11 |  | H25_CTC_AAGTCGTGTGGACTAG | 0 | 0 | 0 | 4 | 39 |
| H41_CTC_CTCGTAGGTTTCCAGG | 0 | 0 | 0 | 0 | 2 |  | H25_CTC_AATCGACGTACAAACA | 0 | 0 | 0 | 0 | 111 |
| H41_CTC_CTCTAATCACCTCGTG | 0 | 1 | 0 | 0 | 28 |  | H25_CTC_ACACAGTGTCACGCTG | 0 | 0 | 0 | 17 | 25 |
| H41_CTC_CTCTCAACATCCAATT | 0 | 0 | 0 | 0 | 5 |  | H25_CTC_ACACAGTTCACCTTGC | 0 | 0 | 0 | 0 | 3 |
| H41_CTC_CTCTCGATCATCAAGT | 0 | 1 | 0 | 3 | 59 |  | H25_CTC_ACACGCGGTCAGTCTA | 0 | 0 | 0 | 0 | 42 |
| H41_CTC_CTGATTAGTTAGGCCC | 1 | 0 | 0 | 0 | 7 |  | H25_CTC_ACATGCAAGGTCACAG | 0 | 0 | 0 | 0 | 5 |
| H41_CTC_CTGCCTATCCTCTCAG | 1 | 0 | 0 | 4 | 22 |  | H25_CTC_ACATTTCAGGGTGGGA | 0 | 0 | 0 | 7 | 92 |
| H41_CTC_CTGCTGCTCCTCGACC | 1 | 0 | 0 | 0 | 52 |  | H25_CTC_ACCAACACACCAGACC | 0 | 0 | 0 | 14 | 187 |
| H41_CTC_CTGGTCAGTCTTGACG | 2 | 0 | 0 | 4 | 27 |  | H25_CTC_ACCATTTCAATTAGGA | 0 | 0 | 0 | 0 | 32 |
| H41_CTC_CTTAACCTCCGGGTAT | 0 | 0 | 1 | 1 | 10 |  | H25_CTC_ACCATTTGTGAGACCA | 0 | 0 | 0 | 1 | 61 |
| H41_CTC_CTTACAAGTTGCACCA | 0 | 0 | 0 | 0 | 25 |  | H25_CTC_ACCCAAATCAATCCCT | 0 | 0 | 0 | 1 | 0 |
| H41_CTC_CTTACGCCAACTGTGG | 0 | 0 | 0 | 4 | 11 |  | H25_CTC_ACCCTCACAGGTGTGA | 0 | 0 | 0 | 0 | 0 |
| H41_CTC_CTTATGAGTGATCAGA | 2 | 0 | 0 | 4 | 18 |  | H25_CTC_ACCCTCATCATTTCCA | 0 | 0 | 0 | 0 | 1 |
| H41_CTC_CTTGACCCAGCCATTC | 0 | 0 | 1 | 5 | 22 |  | H25_CTC_ACCCTTGTCAACCCGG | 0 | 0 | 0 | 0 | 8 |
| H41_CTC_CTTGACTTCCATGCCC | 0 | 0 | 1 | 0 | 14 |  | H25_CTC_ACCGTTCAGTATAGAC | 0 | 0 | 0 | 43 | 105 |
| H41_CTC_CTTGAGGCACTTCGGT | 0 | 0 | 1 | 2 | 14 |  | H25_CTC_ACGATCAAGTACGAGC | 0 | 0 | 0 | 35 | 136 |
| H41_CTC_CTTGGGTAGATTGCAT | 1 | 0 | 0 | 0 | 12 |  | H25_CTC_ACGGTCGCACCATATG | 0 | 0 | 0 | 0 | 2 |
| H41_CTC_CTTGGTAAGCCTGAAC | 0 | 1 | 0 | 0 | 26 |  | H25_CTC_ACGTTCCGTGACTCGC | 0 | 0 | 0 | 6 | 36 |
| H41_CTC_GCAACTATCCGCAACG | 0 | 1 | 0 | 15 | 14 |  | H25_CTC_ACTGTCCAGGGCTGAT | 0 | 0 | 1 | 59 | 111 |
| H41_CTC_GCAATTGTCCCCAGAG | 0 | 0 | 0 | 0 | 5 |  | H25_CTC_ACTGTGATCATGCCGG | 0 | 0 | 0 | 0 | 6 |
| H41_CTC_GCACCGATCCGTGGAT | 0 | 1 | 0 | 0 | 15 |  | H25_CTC_ACTTCCGAGAACGTGC | 0 | 0 | 0 | 1 | 19 |
| H41_CTC_GCACGGTCATCCCGTG | 0 | 0 | 0 | 0 | 13 |  | H25_CTC_ACTTTCAAGTCATGAA | 0 | 0 | 0 | 0 | 31 |
| H41_CTC_GCACTGGTCGATGGAG | 0 | 0 | 0 | 1 | 1 |  | H25_CTC_AGAAATGCATGTCTAG | 0 | 0 | 1 | 0 | 0 |
| H41_CTC_GCAGGATGTCTGGCTA | 0 | 1 | 0 | 0 | 7 |  | H25_CTC_AGACACTTCCAAACCA | 0 | 0 | 0 | 0 | 5 |
| H41_CTC_GCAGGCTCATAACCAA | 0 | 0 | 0 | 0 | 6 |  | H25_CTC_AGACACTTCCCAGGAC | 0 | 0 | 0 | 0 | 53 |
| H41_CTC_GCAGTAAGTATAGACG | 1 | 0 | 0 | 4 | 28 |  | H25_CTC_AGACAGGTCCTTATAC | 0 | 0 | 0 | 0 | 81 |
| H41_CTC_GCCATAGCATTACACT | 0 | 0 | 0 | 8 | 22 |  | H25_CTC_AGACTCAAGCGTTCCG | 0 | 0 | 0 | 12 | 49 |
| H41_CTC_GCCATCTCATTCCTGA | 0 | 1 | 0 | 0 | 53 |  | H25_CTC_AGATCGTCACCGTGGT | 0 | 0 | 0 | 36 | 34 |
| H41_CTC_GCCCAAGTCCGGGCTA | 1 | 0 | 0 | 0 | 63 |  | H25_CTC_AGCATCAAGCCTCAAC | 0 | 0 | 0 | 0 | 1 |
| H41_CTC_GCCCTAAGTTCATCCC | 1 | 0 | 0 | 3 | 31 |  | H25_CTC_AGCATCAAGCCTCAGC | 0 | 0 | 0 | 23 | 50 |
| H41_CTC_GCCTACCTCGACTGAC | 0 | 0 | 0 | 0 | 13 |  | H25_CTC_AGCCACGCATATCTGG | 0 | 0 | 0 | 12 | 27 |
| H41_CTC_GCCTGGATCAGCCAAC | 0 | 0 | 0 | 3 | 22 |  | H25_CTC_AGCCAGCGTAGTGCGA | 0 | 0 | 0 | 16 | 101 |
| H41_CTC_GCCTGTTCAGTACTCC | 0 | 0 | 1 | 0 | 21 |  | H25_CTC_AGCGATTCATAGTCGT | 0 | 0 | 0 | 1 | 106 |
| H41_CTC_GCGATAAGTTAGTAGG | 0 | 0 | 0 | 0 | 54 |  | H25_CTC_AGGAATATCGACATAC | 0 | 0 | 0 | 9 | 50 |
| H41_CTC_GCGGACTCATGAATGC | 1 | 0 | 0 | 2 | 42 |  | H25_CTC_AGGAGGTCAACGACAG | 0 | 0 | 0 | 0 | 0 |
| H41_CTC_GCGGAGTAGGTACGTC | 0 | 0 | 0 | 2 | 10 |  | H25_CTC_AGGCATTTCATCCTAT | 0 | 0 | 0 | 0 | 1 |
| H41_CTC_GCGGCTATCCCGCGAA | 1 | 1 | 0 | 0 | 41 |  | H25_CTC_AGGGTCCAGTACAGAT | 0 | 0 | 0 | 14 | 57 |
| H41_CTC_GCGGGATAGGGCTCCA | 0 | 1 | 0 | 5 | 17 |  | H25_CTC_AGGGTGAAGGACTATA | 0 | 0 | 0 | 0 | 6 |
| H41_CTC_GCGGTAAAGTAGTCCA | 1 | 0 | 1 | 1 | 6 |  | H25_CTC_AGGTAGGTCGTTCCTG | 0 | 0 | 0 | 0 | 96 |
| H41_CTC_GCTAAACCACTCGGAA | 1 | 0 | 0 | 2 | 39 |  | H25_CTC_AGGTCATCAAAGGATT | 0 | 0 | 0 | 0 | 104 |
| H41_CTC_GCTCAGTAGTCATGTG | 0 | 0 | 0 | 0 | 4 |  | H25_CTC_AGGTCATCAGAGGTAC | 0 | 0 | 0 | 8 | 129 |
| H41_CTC_GCTCATCCATCACGTC | 0 | 0 | 0 | 0 | 5 |  | H25_CTC_AGGTCATCATGTGTCA | 0 | 0 | 0 | 0 | 15 |
| H41_CTC_GCTCCTGCAGCTAAGT | 0 | 0 | 0 | 1 | 5 |  | H25_CTC_AGGTCATGTTAGTCGT | 0 | 0 | 0 | 0 | 38 |
| H41_CTC_GCTCTTGTCCACCTAT | 0 | 0 | 0 | 0 | 3 |  | H25_CTC_AGGTTACAGAATTGCA | 0 | 0 | 0 | 12 | 116 |
| H41_CTC_GCTTGTAAGTTAGCGC | 0 | 0 | 0 | 8 | 7 |  | H25_CTC_AGTAGCTGTTTCGGCG | 0 | 0 | 0 | 0 | 59 |
| H41_CTC_GGATAGGTCACAGCTA | 0 | 0 | 0 | 9 | 36 |  | H25_CTC_AGTAGTCAGTGGCGAT | 0 | 0 | 0 | 1 | 51 |
| H41_CTC_GGATCGTTCAGCATCA | 1 | 0 | 0 | 6 | 36 |  | H25_CTC_AGTCAACGTCCCTAAA | 0 | 0 | 0 | 5 | 98 |
| H41_CTC_GGCAACCCATCGTACC | 1 | 0 | 0 | 1 | 24 |  | H25_CTC_AGTCATGGTGATCATC | 0 | 0 | 0 | 0 | 15 |
| H41_CTC_GGCAACTTCAGCTACA | 1 | 0 | 0 | 0 | 17 |  | H25_CTC_AGTCTCCAGCGAGTAC | 0 | 0 | 0 | 5 | 59 |
| H41_CTC_GGCACACCAATCGAGT | 1 | 0 | 0 | 0 | 36 |  | H25_CTC_AGTTCCCAGCTCGAAG | 0 | 0 | 0 | 0 | 117 |
| H41_CTC_GGCCTAACACCATGTC | 0 | 0 | 0 | 1 | 6 |  | H25_CTC_ATACCGACAACGCCCA | 0 | 0 | 0 | 0 | 7 |
| H41_CTC_GGCCTCAAGGGTTGGA | 0 | 0 | 0 | 0 | 2 |  | H25_CTC_ATACCTTAGACTAGAT | 0 | 0 | 0 | 6 | 61 |
| H41_CTC_GGCGCTATCCGTGCAG | 0 | 0 | 0 | 1 | 26 |  | H25_CTC_ATAGACCTCGTGGCTG | 0 | 0 | 0 | 0 | 1 |
| H41_CTC_GGGAACATCGCTTGAA | 1 | 0 | 0 | 0 | 33 |  | H25_CTC_ATAGGCTTCATGGGAG | 0 | 0 | 0 | 0 | 50 |
| H41_CTC_GGGCAAATCAGCACTA | 0 | 1 | 0 | 3 | 13 |  | H25_CTC_ATAGGCTTCTGAGCAT | 0 | 0 | 0 | 0 | 50 |
| H41_CTC_GGGCCTTGTGACTCGC | 0 | 0 | 0 | 3 | 7 |  | H25_CTC_ATATCCTTCTTAGCAG | 0 | 0 | 0 | 0 | 25 |
| H41_CTC_GGGGCAACAACATTAG | 0 | 0 | 0 | 2 | 12 |  | H25_CTC_ATCCCTGAGGCTTAAA | 0 | 0 | 0 | 0 | 0 |
| H41_CTC_GGGTATAGTTCTGGAT | 0 | 0 | 0 | 1 | 36 |  | H25_CTC_ATCCCTGGTTGCAAGG | 0 | 0 | 0 | 0 | 7 |
| H41_CTC_GGGTATGAGCCAGTTA | 0 | 0 | 0 | 0 | 19 |  | H25_CTC_ATCCGTCGTTGGCTAT | 0 | 0 | 0 | 0 | 11 |
| H41_CTC_GGGTCAAAGTCAGCGT | 0 | 0 | 0 | 0 | 12 |  | H25_CTC_ATCGCCTCAGTTCACA | 0 | 0 | 0 | 6 | 55 |
| H41_CTC_GGGTGTCCAATTGACA | 0 | 0 | 0 | 0 | 3 |  | H25_CTC_ATCGGATGTCACAGAG | 0 | 0 | 0 | 0 | 42 |
| H41_CTC_GGGTTGAAGCCAACCG | 1 | 0 | 0 | 4 | 30 |  | H25_CTC_ATCGGCGTCAAGGACG | 0 | 0 | 0 | 1 | 66 |
| H41_CTC_GGTCCTGAGCTTAGAT | 1 | 0 | 0 | 5 | 23 |  | H25_CTC_ATCTTCATCGTCTCAC | 0 | 0 | 0 | 0 | 42 |
| H41_CTC_GGTGCATTCGAATTAC | 1 | 0 | 0 | 0 | 17 |  | H25_CTC_ATGCGATGTATGTCAC | 0 | 0 | 0 | 0 | 19 |
| H41_CTC_GGTGGCAGTTGGCCGT | 0 | 0 | 0 | 0 | 2 |  | H25_CTC_ATGGGTTTCTTTCTAG | 0 | 0 | 0 | 0 | 143 |
| H41_CTC_GGTGGTAAGGCATCCG | 0 | 0 | 0 | 4 | 33 |  | H25_CTC_ATGGTTGGTCTTAGTG | 0 | 0 | 0 | 0 | 1 |
| H41_CTC_GGTGTCCGTCAATGTC | 1 | 0 | 0 | 3 | 18 |  | H25_CTC_ATGTCCCCAACCCGCA | 0 | 0 | 0 | 8 | 55 |
| H41_CTC_GGTTAGCTCGACTGCG | 1 | 0 | 0 | 0 | 16 |  | H25_CTC_ATTCCCGCAAGTGCTT | 0 | 0 | 0 | 12 | 66 |
| H41_CTC_GGTTCAGCACCAATCC | 0 | 0 | 0 | 3 | 36 |  | H25_CTC_ATTCTACAGGAATCGC | 0 | 0 | 0 | 3 | 94 |
| H41_CTC_GTAACCTGTGATGCTT | 0 | 0 | 1 | 12 | 29 |  | H25_CTC_ATTCTACTCCAGTGTA | 0 | 0 | 0 | 6 | 71 |
| H41_CTC_GTAAGTTTCCCCTTAG | 1 | 0 | 0 | 2 | 30 |  | H25_CTC_ATTGTTCCACCTGCGA | 0 | 0 | 0 | 0 | 2 |
| H41_CTC_GTAATCCGTGACTCCA | 0 | 0 | 0 | 8 | 32 |  | H25_CTC_ATTGTTCCAGACCCGT | 0 | 0 | 0 | 0 | 47 |
| H41_CTC_GTAATGGGTGAAGCCG | 0 | 0 | 0 | 2 | 13 |  | H25_CTC_ATTTACCGTGCCTGAC | 0 | 0 | 0 | 1 | 81 |
| H41_CTC_GTACGGGGTCAGGCTC | 1 | 0 | 0 | 4 | 32 |  | H25_CTC_ATTTCACGTAGCGCTC | 0 | 0 | 0 | 14 | 154 |
| H41_CTC_GTACTAACAATACCAA | 0 | 0 | 0 | 2 | 21 |  | H25_CTC_ATTTCTGTCTGGAAGG | 0 | 0 | 0 | 0 | 46 |
| H41_CTC_GTAGGAACAGCTAACG | 2 | 0 | 0 | 4 | 29 |  | H25_CTC_CAACCAACAGAAGCGT | 0 | 0 | 0 | 0 | 120 |
| H41_CTC_GTAGGTGAGCGATGGA | 0 | 0 | 0 | 1 | 8 |  | H25_CTC_CAACCAATCCCAGGCA | 0 | 0 | 0 | 0 | 4 |
| H41_CTC_GTAGTCGCACATGATA | 0 | 1 | 0 | 13 | 25 |  | H25_CTC_CAACCTCCACGACGTC | 0 | 0 | 0 | 0 | 83 |
| H41_CTC_GTATGGCTCCCTGATT | 0 | 0 | 0 | 0 | 3 |  | H25_CTC_CAACCTCCAGAAATTG | 0 | 0 | 0 | 0 | 40 |
| H41_CTC_GTATTAGCAACCGTCT | 1 | 0 | 0 | 2 | 15 |  | H25_CTC_CAATCGACAAAGGCTG | 0 | 0 | 0 | 1 | 29 |
| H41_CTC_GTCAATGTCGACATCA | 0 | 0 | 0 | 6 | 14 |  | H25_CTC_CAATCGACATATGCGT | 0 | 0 | 0 | 2 | 58 |
| H41_CTC_GTCACCCTCGCTAGAC | 0 | 1 | 0 | 8 | 35 |  | H25_CTC_CAATGACAGGATTTGA | 0 | 0 | 0 | 28 | 94 |
| H41_CTC_GTCCAGGTCTGGCCTC | 0 | 0 | 0 | 0 | 7 |  | H25_CTC_CAATGACAGGCGCTTC | 0 | 0 | 0 | 7 | 67 |
| H41_CTC_GTCGAGGGTTCGTCAG | 0 | 0 | 0 | 0 | 5 |  | H25_CTC_CAATGACTCAGCCGTA | 0 | 0 | 0 | 0 | 1 |
| H41_CTC_GTCGGGTCAAGCTATA | 0 | 0 | 0 | 1 | 7 |  | H25_CTC_CAATGACTCAGCTGTA | 0 | 0 | 0 | 0 | 54 |
| H41_CTC_GTCGGGTCAATGGGCC | 1 | 0 | 0 | 3 | 9 |  | H25_CTC_CACACAATCTTTGCGC | 0 | 0 | 0 | 1 | 92 |
| H41_CTC_GTCTACTTCCGGTACG | 0 | 1 | 0 | 0 | 29 |  | H25_CTC_CACGGGTTCATCGCCT | 0 | 0 | 0 | 0 | 61 |
| H41_CTC_GTCTTCACAAACTCGG | 1 | 0 | 0 | 0 | 9 |  | H25_CTC_CACGGGTTCGTCGCCT | 0 | 0 | 0 | 0 | 1 |
| H41_CTC_GTGACGAAGGATCCAC | 2 | 0 | 0 | 22 | 15 |  | H25_CTC_CACGTTCCAGCATACT | 0 | 0 | 0 | 5 | 34 |
| H41_CTC_GTGAGGATCCTTGGAA | 0 | 0 | 0 | 0 | 13 |  | H25_CTC_CACGTTCGTCACTAGT | 0 | 0 | 0 | 25 | 46 |
| H41_CTC_GTGAGTGAGTATGTCG | 1 | 0 | 0 | 5 | 13 |  | H25_CTC_CACTAAGGTCTCCTGT | 0 | 0 | 0 | 73 | 72 |
| H41_CTC_GTGATATTCCCCAACC | 0 | 0 | 0 | 0 | 11 |  | H25_CTC_CACTGGGGTGCGGCTT | 0 | 0 | 0 | 4 | 103 |
| H41_CTC_GTGATGGAGTCTAGGC | 0 | 0 | 0 | 1 | 5 |  | H25_CTC_CAGCAATCACGACAGA | 0 | 0 | 0 | 28 | 157 |
| H41_CTC_GTGGGTTTCCCCAATT | 0 | 0 | 1 | 0 | 26 |  | H25_CTC_CAGGTATAGCATGCAG | 1 | 0 | 0 | 60 | 68 |
| H41_CTC_GTTCACCCACTACCTA | 0 | 0 | 0 | 1 | 7 |  | H25_CTC_CAGTTAGTCAGGAACG | 0 | 0 | 0 | 2 | 30 |
| H41_CTC_GTTGCTGGTGGCTGAG | 0 | 0 | 0 | 9 | 15 |  | H25_CTC_CAGTTCCGTGATAGTA | 0 | 0 | 0 | 57 | 35 |
| H41_CTC_GTTGGGTTCCCCATAT | 0 | 0 | 0 | 2 | 20 |  | H25_CTC_CATACCCCATGCCGAC | 0 | 0 | 0 | 4 | 16 |
| H41_CTC_TAACACTAGGGCTAGA | 0 | 0 | 0 | 2 | 28 |  | H25_CTC_CATCGGGGTACGCTAT | 0 | 0 | 0 | 10 | 124 |
| H41_CTC_TAACGAGGTGAACTGA | 0 | 0 | 0 | 0 | 7 |  | H25_CTC_CATCGGGTCAATCTTC | 0 | 0 | 0 | 0 | 78 |
| H41_CTC_TAACTCCTCAGCATCA | 0 | 0 | 0 | 0 | 15 |  | H25_CTC_CATGAGTAGCTAAACA | 0 | 0 | 0 | 0 | 100 |
| H41_CTC_TAAGCAAAGCAACCGT | 0 | 0 | 0 | 0 | 13 |  | H25_CTC_CATGCGGTCAGGAAAT | 0 | 0 | 0 | 0 | 52 |
| H41_CTC_TAAGCCTGTCAAGGAC | 1 | 1 | 0 | 3 | 51 |  | H25_CTC_CATGGATAGCCAAGGT | 0 | 0 | 0 | 5 | 77 |
| H41_CTC_TAATGGGGTAACCGGG | 0 | 0 | 0 | 1 | 14 |  | H25_CTC_CATGGATTCTGTAACG | 0 | 0 | 0 | 0 | 2 |
| H41_CTC_TAATGGGGTGCGTAGG | 1 | 0 | 0 | 0 | 8 |  | H25_CTC_CATTGAGGTTAAAGTG | 0 | 0 | 0 | 1 | 41 |
| H41_CTC_TACACTAGTATGTGTC | 0 | 1 | 0 | 1 | 23 |  | H25_CTC_CATTGAGGTTGGATCT | 0 | 0 | 0 | 0 | 40 |
| H41_CTC_TACACTTCATCCCAAC | 0 | 0 | 0 | 0 | 13 |  | H25_CTC_CATTGAGTCTCGCTCA | 0 | 0 | 0 | 4 | 101 |
| H41_CTC_TACCAAGTCTATGAAC | 1 | 0 | 0 | 0 | 20 |  | H25_CTC_CATTGTTCATGGGATG | 0 | 0 | 0 | 13 | 76 |
| H41_CTC_TACCACAAGATAGGCG | 1 | 0 | 0 | 2 | 12 |  | H25_CTC_CCAAGCGAGACCCTTA | 0 | 0 | 0 | 0 | 20 |
| H41_CTC_TACCCAAAGGTTACTG | 0 | 1 | 0 | 1 | 1 |  | H25_CTC_CCACTTGCATCTGTTT | 0 | 0 | 0 | 0 | 1 |
| H41_CTC_TACCGCCTCCAGCAAC | 1 | 0 | 0 | 3 | 20 |  | H25_CTC_CCATAAGGTGGCTGCT | 0 | 0 | 0 | 0 | 114 |
| H41_CTC_TACGTCAAGATTCGTG | 0 | 0 | 1 | 10 | 16 |  | H25_CTC_CCCAACTTCTTACACT | 0 | 0 | 0 | 0 | 79 |
| H41_CTC_TACTGTTCATAACGGT | 1 | 0 | 0 | 0 | 6 |  | H25_CTC_CCCGAAGGTGAGCGAT | 0 | 0 | 0 | 1 | 65 |
| H41_CTC_TAGCTGATCTATCGGA | 1 | 0 | 0 | 5 | 36 |  | H25_CTC_CCCTAACAGCTAGAGC | 0 | 0 | 0 | 0 | 37 |
| H41_CTC_TAGGCCACAAGCTTCC | 0 | 1 | 0 | 2 | 54 |  | H25_CTC_CCCTGATTCAGCACCG | 0 | 0 | 0 | 1 | 177 |
| H41_CTC_TAGGGTGAGGTAGTAA | 1 | 0 | 0 | 6 | 37 |  | H25_CTC_CCGATCTAGAACGCGT | 0 | 0 | 0 | 0 | 50 |
| H41_CTC_TAGGTAGGTAATGGCA | 0 | 0 | 0 | 4 | 4 |  | H25_CTC_CCGCAAGCACACCGCA | 0 | 0 | 0 | 0 | 18 |
| H41_CTC_TAGTCAACATCGTTCA | 0 | 0 | 0 | 2 | 8 |  | H25_CTC_CCGGTAGTCGCTAAAC | 0 | 0 | 0 | 0 | 30 |
| H41_CTC_TAGTGGCTCCAGATGC | 0 | 0 | 0 | 0 | 3 |  | H25_CTC_CCGGTGACATATAGCC | 0 | 0 | 0 | 17 | 30 |
| H41_CTC_TATATCCCAAATCATG | 1 | 0 | 0 | 1 | 3 |  | H25_CTC_CCGTTCACACCCTGAG | 0 | 0 | 0 | 1 | 79 |
| H41_CTC_TATCATGGTTAATGGG | 0 | 1 | 0 | 1 | 9 |  | H25_CTC_CCTCACAGTCACCACG | 0 | 0 | 0 | 1 | 83 |
| H41_CTC_TATCCTTGTTTACGCG | 1 | 0 | 0 | 5 | 19 |  | H25_CTC_CCTCCAATCGGACGTC | 0 | 0 | 0 | 43 | 87 |
| H41_CTC_TATGGCTGTTGCGCTA | 0 | 0 | 0 | 0 | 5 |  | H25_CTC_CGAAGTTAGCGGATCA | 0 | 0 | 0 | 0 | 12 |
| H41_CTC_TCAAAGCCATCGACTC | 1 | 0 | 0 | 0 | 29 |  | H25_CTC_CGAAGTTTCCTGTAAG | 0 | 0 | 0 | 0 | 31 |
| H41_CTC_TCAACCTTCCTGCGTC | 0 | 0 | 0 | 0 | 16 |  | H25_CTC_CGAATTGCACAACCGC | 0 | 0 | 0 | 1 | 30 |
| H41_CTC_TCACAATAGCCAGGAA | 1 | 0 | 0 | 6 | 29 |  | H25_CTC_CGACAGCAGAGCTGAC | 0 | 0 | 0 | 2 | 29 |
| H41_CTC_TCACGCCTCAGCTGGT | 0 | 0 | 1 | 11 | 55 |  | H25_CTC_CGACAGCGTTTGATCG | 0 | 0 | 0 | 0 | 112 |
| H41_CTC_TCACGTTTCCCCTGGT | 0 | 2 | 0 | 1 | 14 |  | H25_CTC_CGAGAAGCACATAACC | 0 | 0 | 0 | 0 | 38 |
| H41_CTC_TCAGAGGAGTCGCGTG | 0 | 0 | 1 | 0 | 32 |  | H25_CTC_CGAGGAAGTTGAGGAC | 0 | 0 | 0 | 0 | 21 |
| H41_CTC_TCAGCTGGTAATGAAC | 0 | 0 | 0 | 1 | 18 |  | H25_CTC_CGAGGAATCCATTTGT | 0 | 0 | 0 | 0 | 0 |
| H41_CTC_TCATCGAAGCTGGTCG | 0 | 0 | 0 | 1 | 17 |  | H25_CTC_CGATGGCAGCGGCTCT | 0 | 0 | 0 | 28 | 61 |
| H41_CTC_TCATGCTTCTATGCGA | 0 | 0 | 0 | 5 | 49 |  | H25_CTC_CGATGGCGTCAATCTG | 0 | 0 | 0 | 1 | 104 |
| H41_CTC_TCATTAGGTAATGCCT | 2 | 0 | 0 | 13 | 34 |  | H25_CTC_CGGAACCGTGGAAGTC | 0 | 0 | 0 | 0 | 43 |
| H41_CTC_TCCACACCAAGCCAAT | 0 | 0 | 0 | 2 | 18 |  | H25_CTC_CGGAACCTCTCTCCGA | 0 | 0 | 0 | 20 | 112 |
| H41_CTC_TCCATAAGTTGCTAAC | 0 | 1 | 0 | 0 | 45 |  | H25_CTC_CGGACACAGGGATCAC | 0 | 0 | 0 | 1 | 25 |
| H41_CTC_TCCCTGATCGCGTCCT | 0 | 1 | 0 | 0 | 10 |  | H25_CTC_CGGACACAGGGATTAC | 0 | 0 | 0 | 0 | 1 |
| H41_CTC_TCGCATTTCTGGTGAC | 0 | 1 | 0 | 0 | 42 |  | H25_CTC_CGGCAGTGTGACCGTC | 0 | 0 | 0 | 0 | 1 |
| H41_CTC_TCGCCAAGTTGTCCTC | 1 | 0 | 0 | 1 | 3 |  | H25_CTC_CGTAATGCACTTTAGG | 0 | 0 | 0 | 0 | 0 |
| H41_CTC_TCGCCTATCCTCTATA | 0 | 0 | 0 | 7 | 37 |  | H25_CTC_CGTAGTACATACAGGG | 0 | 0 | 0 | 2 | 86 |
| H41_CTC_TCGCGCAAGGAGCTGA | 0 | 1 | 0 | 0 | 11 |  | H25_CTC_CGTGCTTTCATGCCAA | 0 | 0 | 0 | 0 | 49 |
| H41_CTC_TCGCTTACAAGTGAAG | 0 | 0 | 0 | 5 | 11 |  | H25_CTC_CGTGTCTCACAAGTGG | 0 | 0 | 0 | 21 | 57 |
| H41_CTC_TCGGGGTCATTCCTAG | 0 | 0 | 0 | 0 | 2 |  | H25_CTC_CGTTAGAAGCTGCGAA | 0 | 0 | 0 | 0 | 116 |
| H41_CTC_TCGTCCATCCAATGTG | 0 | 1 | 0 | 0 | 23 |  | H25_CTC_CTAACCCGTCATCGGC | 0 | 0 | 0 | 1 | 11 |
| H41_CTC_TCGTTAAGTTCTGGGC | 0 | 1 | 0 | 2 | 39 |  | H25_CTC_CTAACTTCAGAGTAAT | 0 | 0 | 0 | 0 | 0 |
| H41_CTC_TCTCGAGGTAGCAGTA | 0 | 0 | 0 | 1 | 57 |  | H25_CTC_CTAACTTTCCGCACGA | 0 | 0 | 0 | 120 | 108 |
| H41_CTC_TCTCGATTCTGGTGAC | 0 | 0 | 1 | 0 | 5 |  | H25_CTC_CTACATTTCTCTGACC | 0 | 0 | 0 | 4 | 239 |
| H41_CTC_TCTGATGAGTCGCTAA | 0 | 0 | 0 | 3 | 36 |  | H25_CTC_CTACGGGAGCAAGGAA | 0 | 0 | 0 | 0 | 0 |
| H41_CTC_TGAACAGGTTTCGGAT | 0 | 1 | 0 | 0 | 22 |  | H25_CTC_CTACGGGTCCGATGTA | 0 | 0 | 0 | 0 | 85 |
| H41_CTC_TGACTGATCATGGCGT | 0 | 0 | 0 | 0 | 10 |  | H25_CTC_CTAGACAGTACAATAG | 0 | 0 | 0 | 9 | 56 |
| H41_CTC_TGAGCCACACTTACCA | 2 | 1 | 0 | 0 | 49 |  | H25_CTC_CTATAGGAGCGTTGTT | 0 | 0 | 0 | 0 | 67 |
| H41_CTC_TGAGCTTGTGGCCAAG | 0 | 1 | 0 | 0 | 45 |  | H25_CTC_CTCAACCCAACCCGCA | 0 | 0 | 0 | 0 | 0 |
| H41_CTC_TGAGTAAAGCTTACGA | 0 | 1 | 0 | 0 | 23 |  | H25_CTC_CTCAATTTCAGAGTTC | 0 | 1 | 0 | 3 | 12 |
| H41_CTC_TGATTAGCATGGTGAT | 0 | 2 | 0 | 3 | 49 |  | H25_CTC_CTCACTGCAAACCATC | 0 | 0 | 0 | 7 | 90 |
| H41_CTC_TGCAATGTCACGGACT | 0 | 2 | 0 | 0 | 24 |  | H25_CTC_CTCAGAAGTAGCACAG | 0 | 0 | 0 | 13 | 44 |
| H41_CTC_TGCACGTAGTGGCAGA | 0 | 0 | 1 | 3 | 20 |  | H25_CTC_CTCAGTCCAGAGAATT | 0 | 0 | 0 | 0 | 0 |
| H41_CTC_TGCCAGGTCCAATGTG | 0 | 1 | 0 | 0 | 73 |  | H25_CTC_CTCATCGGTCCCTGTT | 0 | 0 | 0 | 1 | 16 |
| H41_CTC_TGCCTCTGTCAGCCAC | 0 | 0 | 0 | 0 | 7 |  | H25_CTC_CTCATGCCATGGACAG | 0 | 0 | 0 | 1 | 82 |
| H41_CTC_TGCCTTTAGGTGAACG | 1 | 0 | 0 | 0 | 14 |  | H25_CTC_CTCATTAGTTTGTTCT | 0 | 0 | 0 | 0 | 2 |
| H41_CTC_TGCTGGACATAATTCC | 0 | 0 | 0 | 1 | 34 |  | H25_CTC_CTCCAACTCTCCCAAC | 0 | 0 | 0 | 0 | 84 |
| H41_CTC_TGGACGAAGGCGATTC | 0 | 0 | 0 | 0 | 12 |  | H25_CTC_CTCCACAGTTGTAAAG | 0 | 0 | 0 | 14 | 97 |
| H41_CTC_TGGACGTTCATGTCCT | 0 | 0 | 0 | 0 | 11 |  | H25_CTC_CTCCATGAGCTATCTG | 0 | 0 | 0 | 1 | 17 |
| H41_CTC_TGGAGCAAGTGATGCC | 0 | 1 | 0 | 0 | 14 |  | H25_CTC_CTCTCAGAGACCAGCA | 0 | 0 | 0 | 1 | 66 |
| H41_CTC_TGGTAAGGTCTTGATA | 0 | 0 | 0 | 1 | 1 |  | H25_CTC_CTCTCGAAGAAATTCG | 0 | 0 | 0 | 41 | 67 |
| H41_CTC_TGGTCAATCCGGTTGG | 0 | 0 | 1 | 0 | 53 |  | H25_CTC_CTCTGGTTCGCCAGTG | 0 | 0 | 0 | 0 | 0 |
| H41_CTC_TGTAATGCAAGCCGAC | 0 | 0 | 0 | 0 | 7 |  | H25_CTC_CTGCATCGTTCTATCT | 0 | 0 | 0 | 0 | 7 |
| H41_CTC_TGTATCTCAGGAAGGT | 0 | 0 | 0 | 0 | 6 |  | H25_CTC_CTGCCATTCCTCGATC | 0 | 0 | 0 | 0 | 101 |
| H41_CTC_TGTCCCTGTCATCGTG | 0 | 0 | 0 | 0 | 0 |  | H25_CTC_CTGGACGGTATGGAAT | 0 | 0 | 0 | 2 | 58 |
| H41_CTC_TGTCTCACACCTGCCA | 0 | 0 | 0 | 0 | 7 |  | H25_CTC_CTGTCGTTCGCTAAAC | 0 | 0 | 0 | 0 | 51 |
| H42_CTC_AAACCCTGTGCGTGCT | 0 | 0 | 0 | 0 | 10 |  | H25_CTC_CTTACCGAGAGGTCAC | 0 | 0 | 0 | 1 | 158 |
| H42_CTC_AAAGCTAAGCATAGTT | 1 | 0 | 0 | 1 | 29 |  | H25_CTC_CTTACCGTCCCAACTC | 0 | 0 | 0 | 3 | 53 |
| H42_CTC_AAAGGTTGTGATGCTT | 1 | 0 | 0 | 0 | 2 |  | H25_CTC_CTTCAATTCGAGTGAG | 0 | 0 | 0 | 12 | 28 |
| H42_CTC_AAAGTTGAGAATCACT | 2 | 1 | 0 | 0 | 26 |  | H25_CTC_CTTGATTGTGTTGACT | 0 | 0 | 0 | 0 | 22 |
| H42_CTC_AAATGGGCACATACCC | 0 | 0 | 0 | 1 | 16 |  | H25_CTC_CTTGATTTCAGGAAGC | 0 | 0 | 0 | 0 | 76 |
| H42_CTC_AACCATTAGCATGTAT | 0 | 0 | 1 | 0 | 27 |  | H25_CTC_GAAGCGAAGAGCTGCA | 0 | 0 | 0 | 0 | 43 |
| H42_CTC_AACCCTGGTTTGGGAA | 1 | 0 | 0 | 0 | 18 |  | H25_CTC_GACATCAGTATAGGAT | 0 | 0 | 0 | 0 | 3 |
| H42_CTC_AACCTAGTCATGCAGC | 0 | 2 | 0 | 0 | 24 |  | H25_CTC_GACCAATAGAAATCCA | 0 | 0 | 0 | 0 | 2 |
| H42_CTC_AACGCCACACTCAGCT | 0 | 1 | 0 | 0 | 7 |  | H25_CTC_GACCCAGAGACGTCCC | 0 | 0 | 0 | 21 | 23 |
| H42_CTC_AACGGTGAGTCAACTG | 0 | 2 | 0 | 0 | 2 |  | H25_CTC_GACCCAGCACTTTATC | 0 | 0 | 0 | 4 | 40 |
| H42_CTC_AACGTAAAGGCTCTCC | 1 | 0 | 0 | 0 | 18 |  | H25_CTC_GACCCTTAGATGAATC | 0 | 0 | 0 | 0 | 162 |
| H42_CTC_AACTAGAAGCAGGTGA | 1 | 0 | 0 | 0 | 0 |  | H25_CTC_GACTATGCAGCAGACA | 0 | 0 | 0 | 1 | 14 |
| H42_CTC_AACTTCCTCAATTCGG | 1 | 2 | 0 | 0 | 17 |  | H25_CTC_GACTCAATCCTAACAG | 0 | 0 | 0 | 7 | 75 |
| H42_CTC_AACTTCCTCAGTATCG | 1 | 0 | 0 | 0 | 15 |  | H25_CTC_GAGAAATAGTTATGGA | 0 | 0 | 0 | 8 | 84 |
| H42_CTC_AACTTTGGTGACGAGT | 0 | 1 | 0 | 2 | 26 |  | H25_CTC_GAGAAATTCCTCAGGG | 0 | 0 | 0 | 13 | 61 |
| H42_CTC_AACTTTGGTTTACTGA | 0 | 1 | 0 | 0 | 32 |  | H25_CTC_GAGGGATGTAAGATAC | 0 | 0 | 0 | 0 | 39 |
| H42_CTC_AAGCTAGGTTCACTGG | 0 | 1 | 0 | 0 | 45 |  | H25_CTC_GAGTCTATCATCACAG | 0 | 0 | 0 | 0 | 82 |
| H42_CTC_AAGGCCAAGGCTCCAG | 0 | 1 | 0 | 0 | 5 |  | H25_CTC_GAGTTACAGGACTATA | 0 | 0 | 0 | 0 | 16 |
| H42_CTC_AAGGGGTCATAGGTTA | 0 | 4 | 0 | 0 | 28 |  | H25_CTC_GAGTTGTAGCGCTTCG | 0 | 0 | 0 | 0 | 24 |
| H42_CTC_AAGTACGGTGCATCTG | 0 | 0 | 0 | 0 | 4 |  | H25_CTC_GAGTTTGAGTTAACGA | 0 | 0 | 0 | 0 | 30 |
| H42_CTC_AAGTGTCCATGCAGAT | 1 | 0 | 0 | 10 | 36 |  | H25_CTC_GATAGAACACGCGCTA | 0 | 0 | 0 | 5 | 63 |
| H42_CTC_AATACGAAGTATGGCC | 1 | 0 | 0 | 0 | 18 |  | H25_CTC_GATCATGCAGCAGATG | 0 | 0 | 0 | 0 | 3 |
| H42_CTC_AATACGGGTTCCTGAG | 1 | 1 | 0 | 0 | 35 |  | H25_CTC_GATGATCAGCGCCTTG | 0 | 0 | 0 | 4 | 43 |
| H42_CTC_AATCAGCCATCACGTC | 0 | 0 | 0 | 0 | 44 |  | H25_CTC_GATGCTAAGTGGTCAG | 0 | 0 | 0 | 5 | 46 |
| H42_CTC_AATGTGAAGTCGCTGG | 1 | 0 | 0 | 0 | 10 |  | H25_CTC_GCACGTGCAGTACTAC | 0 | 0 | 0 | 0 | 31 |
| H42_CTC_AATTGTCAGGCACAGA | 1 | 0 | 0 | 0 | 11 |  | H25_CTC_GCAGCCAGTTGGACTT | 0 | 0 | 0 | 0 | 93 |
| H42_CTC_ACAAGCCTCAATGGTT | 0 | 0 | 0 | 0 | 5 |  | H25_CTC_GCAGGCTAGTACCATC | 0 | 0 | 0 | 1 | 16 |
| H42_CTC_ACAATGAAGGTCAATT | 0 | 0 | 0 | 1 | 32 |  | H25_CTC_GCATCGGTCAGCGCGT | 0 | 0 | 0 | 4 | 16 |
| H42_CTC_ACAATGGGTAGCCTTG | 0 | 1 | 0 | 1 | 40 |  | H25_CTC_GCATTAGCAAGCGGAT | 0 | 0 | 0 | 1 | 71 |
| H42_CTC_ACACATTAGCCACGCA | 0 | 1 | 0 | 0 | 11 |  | H25_CTC_GCATTAGCAGCGTTGC | 0 | 0 | 0 | 11 | 18 |
| H42_CTC_ACAGACAGTTATGGCT | 1 | 0 | 0 | 0 | 97 |  | H25_CTC_GCCATGGAGACTGGGT | 0 | 0 | 0 | 0 | 66 |
| H42_CTC_ACAGCTCAGCCAGCCA | 0 | 2 | 0 | 0 | 54 |  | H25_CTC_GCGAGAAGTGGCCTCA | 0 | 0 | 0 | 0 | 1 |
| H42_CTC_ACAGGATAGGCACATC | 0 | 1 | 0 | 0 | 8 |  | H25_CTC_GCGATCGAGCCGAATG | 0 | 0 | 0 | 0 | 80 |
| H42_CTC_ACAGGGGCAAGTCCAG | 0 | 0 | 1 | 0 | 5 |  | H25_CTC_GCTGCAGGTCTCGCGA | 0 | 0 | 0 | 0 | 29 |
| H42_CTC_ACATAGAAGTGACGAG | 0 | 0 | 0 | 3 | 12 |  | H25_CTC_GCTTCACTCGCTAATG | 0 | 0 | 0 | 0 | 74 |
| H42_CTC_ACATCCGGTCTAGCAT | 1 | 0 | 0 | 1 | 13 |  | H25_CTC_GGAACCCAGGTAAGTT | 0 | 0 | 0 | 0 | 23 |
| H42_CTC_ACATGAAAGGTTCCTT | 0 | 3 | 0 | 0 | 10 |  | H25_CTC_GGAGAACCAACTGGTT | 0 | 0 | 0 | 2 | 35 |
| H42_CTC_ACATGGTCAATTAGCA | 1 | 0 | 0 | 0 | 4 |  | H25_CTC_GGAGCAATCACGTCCT | 0 | 0 | 0 | 8 | 30 |
| H42_CTC_ACCAAGTCAACCTGCT | 0 | 0 | 0 | 0 | 28 |  | H25_CTC_GGAGCAATCAGGGATG | 0 | 0 | 0 | 0 | 101 |
| H42_CTC_ACCCAGACACTCATAT | 0 | 0 | 0 | 6 | 57 |  | H25_CTC_GGAGGTAGTACGTAGG | 0 | 0 | 0 | 0 | 47 |
| H42_CTC_ACCCCTTGTGATGCGG | 0 | 0 | 0 | 1 | 24 |  | H25_CTC_GGATCTAAGTTGGAAT | 0 | 0 | 0 | 0 | 35 |
| H42_CTC_ACCCGCAAGCCGATTA | 0 | 0 | 0 | 3 | 19 |  | H25_CTC_GGATCTATCACTGAAC | 0 | 0 | 0 | 1 | 24 |
| H42_CTC_ACCCGCAAGCGGACTT | 0 | 0 | 0 | 0 | 18 |  | H25_CTC_GGATGTTGTCCGGCAT | 0 | 0 | 0 | 11 | 34 |
| H42_CTC_ACCTATGAGCTACGCG | 1 | 0 | 0 | 0 | 1 |  | H25_CTC_GGCTTGGAGCCGTCGT | 0 | 0 | 0 | 7 | 118 |
| H42_CTC_ACCTGGGTCTAACCCT | 2 | 0 | 0 | 0 | 29 |  | H25_CTC_GGCTTGGTCCGAGTGC | 0 | 0 | 0 | 0 | 51 |
| H42_CTC_ACGACATCAAACTGAC | 0 | 0 | 0 | 0 | 8 |  | H25_CTC_GGGACAAGTAACGCGA | 0 | 0 | 0 | 0 | 5 |
| H42_CTC_ACGAGTGAGAAGGATC | 1 | 0 | 0 | 0 | 17 |  | H25_CTC_GGGACAATCTTAGCTT | 0 | 0 | 0 | 0 | 4 |
| H42_CTC_ACGATAGGTGAAGGCT | 0 | 3 | 0 | 0 | 26 |  | H25_CTC_GGGACCTAGGGCTGAT | 0 | 0 | 0 | 4 | 121 |
| H42_CTC_ACGCCCAAGCGCTTAA | 0 | 0 | 0 | 0 | 11 |  | H25_CTC_GGGAGTACAAAGACGC | 0 | 0 | 0 | 13 | 113 |
| H42_CTC_ACGGAGGCATGCTCTA | 0 | 0 | 0 | 0 | 5 |  | H25_CTC_GGGTATTCAATGTCTG | 0 | 0 | 0 | 4 | 85 |
| H42_CTC_ACGGCTTAGTACGCCG | 0 | 0 | 0 | 1 | 34 |  | H25_CTC_GGGTTTAGTGTGTGGA | 0 | 0 | 0 | 0 | 1 |
| H42_CTC_ACGTCACAGAACGGCA | 0 | 0 | 0 | 0 | 7 |  | H25_CTC_GGTGGCTCAATAACCC | 0 | 0 | 0 | 13 | 38 |
| H42_CTC_ACGTTTAGTCCATATA | 0 | 1 | 0 | 0 | 66 |  | H25_CTC_GGTTCTCTCGCCGTGA | 0 | 0 | 0 | 0 | 117 |
| H42_CTC_ACTAAGGGTCTGTCGA | 0 | 1 | 0 | 0 | 21 |  | H25_CTC_GTAAGTCGTCATATGC | 0 | 0 | 0 | 16 | 33 |
| H42_CTC_ACTATCCTCTGTCTGT | 1 | 0 | 0 | 1 | 23 |  | H25_CTC_GTAATCGAGTGTGTTC | 0 | 0 | 0 | 0 | 21 |
| H42_CTC_ACTCACTTCCAGCAAC | 1 | 0 | 0 | 0 | 13 |  | H25_CTC_GTAGGTTAGCCTCTTC | 0 | 0 | 0 | 57 | 117 |
| H42_CTC_ACTGTGATCGCTAGTA | 1 | 0 | 0 | 0 | 50 |  | H25_CTC_GTAGGTTAGTCTCTTC | 0 | 0 | 0 | 1 | 8 |
| H42_CTC_ACTGTGATCTTGACTC | 1 | 0 | 0 | 1 | 3 |  | H25_CTC_GTAGTACGTCTCGGGT | 0 | 0 | 0 | 0 | 1 |
| H42_CTC_ACTGTTAGTCCATCGA | 0 | 0 | 0 | 0 | 5 |  | H25_CTC_GTATTGGAGTACCATC | 0 | 0 | 0 | 2 | 46 |
| H42_CTC_ACTTGGGCATCACTGC | 0 | 0 | 0 | 1 | 32 |  | H25_CTC_GTCCCATAGCACGTCC | 0 | 0 | 0 | 1 | 56 |
| H42_CTC_AGACAACAGGCGTACT | 0 | 0 | 0 | 0 | 3 |  | H25_CTC_GTCCCATTCTGCACCT | 0 | 0 | 0 | 2 | 80 |
| H42_CTC_AGACACAGTAAGTCGA | 1 | 0 | 0 | 0 | 6 |  | H25_CTC_GTCGAATCAAAGAGTT | 0 | 0 | 0 | 6 | 26 |
| H42_CTC_AGACACAGTGAGACAC | 0 | 1 | 0 | 0 | 5 |  | H25_CTC_GTCGAATCACTTGGGC | 0 | 0 | 0 | 4 | 43 |
| H42_CTC_AGAGTCAGTCTCACTC | 0 | 1 | 0 | 0 | 16 |  | H25_CTC_GTCGAATTCGCGATCG | 0 | 0 | 0 | 2 | 64 |
| H42_CTC_AGCACCACAACCCTGT | 0 | 0 | 0 | 0 | 3 |  | H25_CTC_GTCGTAAAGTTGGAAT | 0 | 0 | 0 | 0 | 80 |
| H42_CTC_AGCACTATCACCCTGC | 0 | 1 | 0 | 0 | 70 |  | H25_CTC_GTCGTTCCACACCTTC | 0 | 0 | 0 | 0 | 36 |
| H42_CTC_AGCATCGCATAACTAG | 1 | 0 | 0 | 0 | 12 |  | H25_CTC_GTCTAGAGTTCCACGG | 0 | 0 | 0 | 0 | 76 |
| H42_CTC_AGCCCCAAGATTACGT | 0 | 0 | 0 | 0 | 1 |  | H25_CTC_GTCTGTCAGCAAACAT | 0 | 0 | 0 | 9 | 35 |
| H42_CTC_AGCCTGACAATCGTGG | 2 | 4 | 0 | 0 | 22 |  | H25_CTC_GTGAGCCGTGTGGTCC | 0 | 0 | 0 | 12 | 26 |
| H42_CTC_AGCGAGGCAGCCAGTA | 1 | 0 | 0 | 0 | 4 |  | H25_CTC_GTGCTGGAGTATGGCG | 0 | 0 | 0 | 15 | 21 |
| H42_CTC_AGCGATGAGTCAGGGA | 0 | 1 | 0 | 0 | 23 |  | H25_CTC_GTGCTGGCATCCGAGC | 0 | 0 | 0 | 4 | 73 |
| H42_CTC_AGCGCAGGTTCGTGGC | 0 | 1 | 0 | 0 | 17 |  | H25_CTC_GTGCTGGGTGATTAGA | 0 | 0 | 0 | 1 | 158 |
| H42_CTC_AGCGGGACAAGCCTCT | 0 | 1 | 0 | 0 | 24 |  | H25_CTC_GTGCTGGTCTGATGGT | 0 | 0 | 0 | 0 | 0 |
| H42_CTC_AGCTTAGTCGATACTG | 0 | 1 | 0 | 0 | 1 |  | H25_CTC_GTGTGGCTCCCATGGG | 0 | 0 | 0 | 7 | 38 |
| H42_CTC_AGGAAATCAAGTAAGG | 0 | 1 | 0 | 0 | 7 |  | H25_CTC_GTTAGTGCAAAGGGCT | 0 | 0 | 0 | 6 | 70 |
| H42_CTC_AGGCCATTCAATCCCT | 1 | 2 | 0 | 0 | 24 |  | H25_CTC_GTTAGTGTCAAACCCA | 0 | 0 | 0 | 0 | 4 |
| H42_CTC_AGGCGAATCTATCGGA | 0 | 0 | 0 | 0 | 11 |  | H25_CTC_GTTATGGTCGCCAACG | 0 | 0 | 0 | 0 | 37 |
| H42_CTC_AGGGCAATCCTTCGGC | 0 | 0 | 0 | 0 | 17 |  | H25_CTC_GTTCATTTCTGAGGTT | 0 | 0 | 0 | 0 | 11 |
| H42_CTC_AGGGTCAAGCGGTAGA | 0 | 3 | 0 | 0 | 7 |  | H25_CTC_GTTGCGGGTCTGCCTT | 0 | 0 | 0 | 34 | 75 |
| H42_CTC_AGTAACCCATGGATCG | 0 | 0 | 0 | 0 | 30 |  | H25_CTC_GTTGCTCCAGTTAAAG | 0 | 0 | 0 | 12 | 72 |
| H42_CTC_AGTCACGCAGCTCTAC | 1 | 0 | 0 | 0 | 38 |  | H25_CTC_GTTGTAGCAGGCCTGT | 0 | 0 | 0 | 0 | 1 |
| H42_CTC_AGTCCCAGTAACGGGT | 0 | 1 | 0 | 0 | 11 |  | H25_CTC_GTTGTAGTCTGCGGAC | 0 | 0 | 0 | 1 | 35 |
| H42_CTC_AGTCCCAGTAGTTGCT | 0 | 0 | 0 | 0 | 10 |  | H25_CTC_GTTGTCCCACACAGCC | 0 | 0 | 0 | 10 | 16 |
| H42_CTC_AGTGAGACAAACCGAT | 0 | 0 | 0 | 0 | 12 |  | H25_CTC_GTTTACTAGCACCGAA | 1 | 0 | 0 | 39 | 58 |
| H42_CTC_AGTGAGTAGTTAGATC | 0 | 1 | 0 | 0 | 44 |  | H25_CTC_GTTTACTGTCACTCTC | 0 | 0 | 0 | 0 | 108 |
| H42_CTC_AGTGATTTCTAACCCT | 0 | 0 | 0 | 6 | 22 |  | H25_CTC_GTTTGGATCGTCGATA | 0 | 0 | 0 | 0 | 49 |
| H42_CTC_AGTGCAAGTGGCTGTC | 0 | 2 | 0 | 1 | 32 |  | H25_CTC_TAAGCACAGCCTTTCC | 0 | 0 | 0 | 0 | 93 |
| H42_CTC_AGTGTGAGTTGAATCC | 0 | 0 | 0 | 0 | 18 |  | H25_CTC_TAATTCCAGAAGCTCG | 0 | 0 | 0 | 0 | 63 |
| H42_CTC_AGTGTTGTCACAGACC | 0 | 1 | 0 | 0 | 4 |  | H25_CTC_TAATTCCGTGGTCTAT | 0 | 0 | 0 | 0 | 0 |
| H42_CTC_ATAGTCCGTGGGTTAA | 0 | 1 | 0 | 6 | 61 |  | H25_CTC_TACCCACAGACGCAGT | 0 | 0 | 0 | 0 | 71 |
| H42_CTC_ATAGTCCGTTTAGTTG | 0 | 1 | 0 | 0 | 17 |  | H25_CTC_TACCGAACATTACTCT | 0 | 0 | 0 | 0 | 0 |
| H42_CTC_ATATGGCCAAGCATAC | 0 | 0 | 0 | 0 | 0 |  | H25_CTC_TACCGGGTCTAATTCC | 0 | 0 | 0 | 0 | 1 |
| H42_CTC_ATCAGGTCATAGATGC | 0 | 1 | 0 | 0 | 26 |  | H25_CTC_TACCTGCAGGATGTTA | 0 | 0 | 0 | 0 | 4 |
| H42_CTC_ATCCCCATCGCTATCT | 0 | 0 | 0 | 0 | 9 |  | H25_CTC_TACCTGCCATTGGGAG | 0 | 0 | 0 | 0 | 156 |
| H42_CTC_ATCCTAGAGGAACCGA | 0 | 0 | 0 | 0 | 37 |  | H25_CTC_TACGCTCTCAGCCTCT | 0 | 0 | 0 | 13 | 38 |
| H42_CTC_ATCCTGTTCACGTTAG | 0 | 1 | 0 | 4 | 30 |  | H25_CTC_TAGATCGGTCTGTCCT | 0 | 0 | 0 | 0 | 73 |
| H42_CTC_ATCGAGGAGTAGACCC | 0 | 1 | 0 | 0 | 7 |  | H25_CTC_TAGCACAGTTCTTGCC | 0 | 0 | 0 | 2 | 17 |
| H42_CTC_ATCGGTCAGGTCAACC | 1 | 0 | 0 | 0 | 9 |  | H25_CTC_TAGGAGGGTTAAACCC | 0 | 0 | 0 | 0 | 151 |
| H42_CTC_ATCGTCAAGCCTACCC | 1 | 0 | 0 | 1 | 9 |  | H25_CTC_TATACCTGTACAGCGA | 0 | 0 | 0 | 3 | 5 |
| H42_CTC_ATCTACCTCAGCATTG | 0 | 0 | 0 | 1 | 37 |  | H25_CTC_TATCTGTAGGCAGGGA | 0 | 0 | 0 | 2 | 36 |
| H42_CTC_ATCTATGGTTAGACGA | 0 | 2 | 0 | 0 | 10 |  | H25_CTC_TATGTTCAGATGCTAA | 0 | 0 | 0 | 4 | 104 |
| H42_CTC_ATCTCATCACATACGG | 1 | 0 | 0 | 0 | 13 |  | H25_CTC_TATGTTCAGCAAGTGC | 0 | 0 | 0 | 4 | 64 |
| H42_CTC_ATCTGCAAGTTGCATG | 0 | 1 | 0 | 0 | 28 |  | H25_CTC_TCAAGCAAGCATTTGC | 0 | 0 | 0 | 2 | 25 |
| H42_CTC_ATGATGAAGTTAACCG | 0 | 2 | 0 | 0 | 25 |  | H25_CTC_TCAAGCAAGCTACTGT | 0 | 0 | 0 | 66 | 85 |
| H42_CTC_ATGCTTGAGGTGGACA | 0 | 1 | 0 | 0 | 27 |  | H25_CTC_TCAAGCACATCGGAGA | 0 | 0 | 0 | 22 | 52 |
| H42_CTC_ATGGGCAAGGGGATCA | 0 | 0 | 0 | 0 | 7 |  | H25_CTC_TCAATTCTCCATACTT | 0 | 0 | 0 | 0 | 14 |
| H42_CTC_ATGGGGATCAGCATCA | 0 | 0 | 0 | 0 | 7 |  | H25_CTC_TCACGCTTCGTTGTAG | 0 | 0 | 0 | 0 | 63 |
| H42_CTC_ATGGGTGAGATAGTTC | 2 | 0 | 0 | 0 | 8 |  | H25_CTC_TCAGCAAAGGTGCTTT | 0 | 0 | 0 | 0 | 0 |
| H42_CTC_ATGGTTTAGCGAGACA | 0 | 1 | 0 | 2 | 18 |  | H25_CTC_TCAGGGCTCATCGCTC | 0 | 0 | 0 | 1 | 160 |
| H42_CTC_ATGTACCGTCTAAGCT | 0 | 1 | 0 | 0 | 31 |  | H25_CTC_TCAGTCCTCGTTCTGC | 0 | 0 | 0 | 0 | 1 |
| H42_CTC_ATGTGAGGTGGGTCTC | 0 | 1 | 0 | 0 | 5 |  | H25_CTC_TCATCATAGTCGGCAA | 0 | 0 | 0 | 1 | 2 |
| H42_CTC_ATGTGCATCCAGAGGA | 0 | 0 | 0 | 2 | 24 |  | H25_CTC_TCATCCGGTGCCGGTT | 0 | 0 | 0 | 0 | 1 |
| H42_CTC_ATGTGTTAGGTATGAA | 0 | 1 | 0 | 0 | 11 |  | H25_CTC_TCATGCCCAAATGCTC | 0 | 0 | 0 | 0 | 0 |
| H42_CTC_ATGTTCCTCCTCGTTG | 0 | 2 | 0 | 0 | 10 |  | H25_CTC_TCATTGTAGAGGTCGT | 0 | 0 | 0 | 1 | 48 |
| H42_CTC_ATTCCAAGTGATAGGC | 1 | 0 | 0 | 0 | 16 |  | H25_CTC_TCCACGTCACGTGTGC | 0 | 0 | 0 | 2 | 83 |
| H42_CTC_ATTGACTAGCTAACGC | 1 | 0 | 0 | 0 | 15 |  | H25_CTC_TCCTCGAGTACACTCA | 0 | 0 | 0 | 11 | 219 |
| H42_CTC_ATTGACTAGGAGGCGT | 0 | 1 | 0 | 0 | 1 |  | H25_CTC_TCGATTTAGTAAAGCT | 0 | 0 | 0 | 0 | 29 |
| H42_CTC_CAAACCGCATTACCGC | 0 | 1 | 0 | 0 | 8 |  | H25_CTC_TCGCAGGGTCCAGTTA | 0 | 0 | 0 | 1 | 81 |
| H42_CTC_CAAAGGCGTCAGTTAC | 0 | 0 | 0 | 3 | 32 |  | H25_CTC_TCGCAGGGTTTCACTT | 0 | 0 | 0 | 27 | 98 |
| H42_CTC_CAACAGTCATGACACG | 0 | 0 | 0 | 0 | 30 |  | H25_CTC_TCGGGACCAACGCATT | 0 | 0 | 0 | 0 | 6 |
| H42_CTC_CAACCCAGTAGTGCAA | 1 | 1 | 0 | 0 | 4 |  | H25_CTC_TCGTAGAGTGTTCAGT | 0 | 0 | 0 | 0 | 48 |
| H42_CTC_CAACGAAGTTAGACAG | 0 | 0 | 0 | 0 | 48 |  | H25_CTC_TCGTCCACAGACAAAT | 0 | 0 | 0 | 0 | 66 |
| H42_CTC_CAACGAAGTTTACAGC | 1 | 0 | 0 | 0 | 14 |  | H25_CTC_TCTACCGAGAAGCCAC | 0 | 0 | 0 | 1 | 104 |
| H42_CTC_CAACGGGGTTTAAGCC | 2 | 0 | 0 | 1 | 35 |  | H25_CTC_TCTCAGCCAAAGAGTT | 0 | 0 | 0 | 1 | 58 |
| H42_CTC_CAACTGATCCTGCCTA | 1 | 0 | 0 | 0 | 8 |  | H25_CTC_TCTGCCAGTTTCGTTT | 0 | 0 | 0 | 0 | 22 |
| H42_CTC_CAAGCCCGTTATCGCG | 0 | 0 | 0 | 0 | 11 |  | H25_CTC_TCTGGCTAGGCTCTCG | 0 | 0 | 0 | 0 | 70 |
| H42_CTC_CACAATTAGTTACGAT | 0 | 1 | 0 | 0 | 0 |  | H25_CTC_TCTTAGTGTCTCCCTA | 0 | 0 | 0 | 0 | 26 |
| H42_CTC_CACATTAGTCAGGAAC | 1 | 0 | 0 | 0 | 25 |  | H25_CTC_TGACAGTTCGTAGCTA | 0 | 0 | 0 | 12 | 23 |
| H42_CTC_CACCAAATCGCTCATT | 0 | 0 | 1 | 0 | 9 |  | H25_CTC_TGACGCGCATGGGATG | 0 | 0 | 0 | 0 | 34 |
| H42_CTC_CACGACTAGGCTCCTC | 0 | 0 | 0 | 0 | 10 |  | H25_CTC_TGAGCGCCACAAGTGG | 0 | 0 | 0 | 0 | 0 |
| H42_CTC_CACGAGGGTCTGGCAC | 0 | 0 | 0 | 3 | 21 |  | H25_CTC_TGAGTCAGTCGGATTT | 0 | 0 | 0 | 0 | 19 |
| H42_CTC_CACTACCCACCATGAG | 1 | 0 | 0 | 0 | 20 |  | H25_CTC_TGAGTCATCTTTGATC | 0 | 0 | 0 | 1 | 57 |
| H42_CTC_CACTACTTCACACCGG | 0 | 0 | 0 | 0 | 4 |  | H25_CTC_TGATCAGCATCTGCGG | 0 | 0 | 0 | 14 | 46 |
| H42_CTC_CACTTCACATAGATTG | 1 | 0 | 0 | 0 | 1 |  | H25_CTC_TGATCTTCAAGCAATA | 0 | 0 | 0 | 1 | 53 |
| H42_CTC_CAGCCAACAGTTGCGA | 0 | 0 | 0 | 0 | 32 |  | H25_CTC_TGATTCTAGCGCTGAA | 0 | 0 | 0 | 0 | 31 |
| H42_CTC_CAGCGATTCCAGCGAT | 1 | 0 | 0 | 0 | 16 |  | H25_CTC_TGCATCCGTGTTCCTC | 0 | 0 | 0 | 23 | 58 |
| H42_CTC_CAGGATAGTGAACGTA | 0 | 1 | 0 | 0 | 10 |  | H25_CTC_TGCCGAGAGCATTGTC | 0 | 0 | 0 | 10 | 35 |
| H42_CTC_CAGGCACCAGCATGAT | 0 | 1 | 0 | 0 | 17 |  | H25_CTC_TGCGACGCAGGTTACT | 0 | 0 | 0 | 14 | 73 |
| H42_CTC_CAGGTCCGTGCGTGCT | 0 | 0 | 0 | 17 | 33 |  | H25_CTC_TGCTCGTCAAGTCCCG | 0 | 0 | 0 | 8 | 75 |
| H42_CTC_CAGTTATAGGCACTCG | 0 | 1 | 0 | 0 | 6 |  | H25_CTC_TGCTGAACACGTTCGG | 0 | 0 | 0 | 11 | 75 |
| H42_CTC_CATACAACAGCACTGA | 0 | 0 | 0 | 0 | 17 |  | H25_CTC_TGGAGAGAGTACGAGC | 0 | 0 | 0 | 14 | 41 |
| H42_CTC_CATAGGGAGTGCCCAG | 1 | 0 | 0 | 1 | 24 |  | H25_CTC_TGGCGTGCATTCAGCA | 0 | 0 | 0 | 21 | 51 |
| H42_CTC_CATATGGTCTCACTTA | 0 | 0 | 0 | 0 | 13 |  | H25_CTC_TGGGTTAAGCATCCTA | 0 | 0 | 0 | 0 | 79 |
| H42_CTC_CATCCCTGTGAACTGA | 0 | 0 | 0 | 1 | 28 |  | H25_CTC_TGGGTTAAGTTTCTTC | 0 | 0 | 0 | 0 | 8 |
| H42_CTC_CATGCGCGTATGGCAA | 0 | 2 | 0 | 0 | 17 |  | H25_CTC_TGGTGATGTTCACCGG | 0 | 0 | 0 | 1 | 104 |
| H42_CTC_CCAAATGTCAAGTGAA | 0 | 1 | 0 | 0 | 15 |  | H25_CTC_TGGTTAGAGTCACAGG | 0 | 0 | 0 | 1 | 64 |
| H42_CTC_CCAACTAAGCGCGATT | 1 | 0 | 0 | 0 | 3 |  | H25_CTC_TGTAGACAGTCGGCCT | 0 | 0 | 0 | 1 | 103 |
| H42_CTC_CCACACAGTCAATCTA | 0 | 0 | 0 | 0 | 3 |  | H25_CTC_TGTCAGAAGCACCCAC | 0 | 0 | 0 | 0 | 42 |
| H42_CTC_CCACCTGCATAGATGC | 0 | 0 | 0 | 0 | 24 |  | H25_CTC_TGTCAGATCCAACACA | 0 | 0 | 0 | 6 | 44 |
| H42_CTC_CCAGAGGGTTTGACCG | 0 | 1 | 0 | 0 | 14 |  | H25_CTC_TGTGAGTAGGTAGCCA | 0 | 0 | 0 | 0 | 76 |
| H42_CTC_CCATACTTCTTGATCC | 0 | 1 | 0 | 0 | 8 |  | H25_CTC_TGTGAGTTCTTCGTAT | 0 | 0 | 0 | 0 | 69 |
| H42_CTC_CCATGCCTCGCGCAAT | 0 | 0 | 0 | 2 | 28 |  | H25_CTC_TTACCATGTGGAGGTT | 0 | 0 | 0 | 0 | 49 |
| H42_CTC_CCCATAGGTATGGCTT | 0 | 1 | 0 | 5 | 62 |  | H25_CTC_TTACGCCGTCCAGCCA | 0 | 0 | 0 | 0 | 65 |
| H42_CTC_CCCATCGCACATACCC | 1 | 0 | 0 | 0 | 13 |  | H25_CTC_TTAGGCATCGAAGCAG | 0 | 0 | 0 | 0 | 60 |
| H42_CTC_CCCGATGAGCGATAGG | 0 | 1 | 0 | 3 | 9 |  | H25_CTC_TTAGGGTAGCCATGCC | 0 | 0 | 0 | 0 | 6 |
| H42_CTC_CCCGCATTCCAGCCCT | 1 | 0 | 0 | 0 | 27 |  | H25_CTC_TTAGTCTGTTAGAAAC | 0 | 0 | 0 | 0 | 87 |
| H42_CTC_CCCTATGTCAGTAGCC | 1 | 1 | 0 | 0 | 25 |  | H25_CTC_TTATTGCGTAACATAG | 0 | 0 | 0 | 0 | 34 |
| H42_CTC_CCGCAAGTCGTGAGAG | 1 | 0 | 0 | 0 | 33 |  | H25_CTC_TTCATTGGTCTGTAAC | 0 | 0 | 0 | 0 | 104 |
| H42_CTC_CCGGATTAGGCACGTT | 0 | 0 | 0 | 1 | 17 |  | H25_CTC_TTCCGTGTCCAAATGC | 0 | 0 | 0 | 0 | 0 |
| H42_CTC_CCGGTATAGCCTCTAT | 1 | 0 | 0 | 0 | 10 |  | H25_CTC_TTCCTTCCAATGGCCC | 0 | 0 | 0 | 0 | 5 |
| H42_CTC_CCGTAACAGCCGAGTT | 0 | 1 | 0 | 0 | 4 |  | H25_CTC_TTCGGTCCAAGATTGA | 0 | 0 | 0 | 1 | 32 |
| H42_CTC_CCGTGTTCACTAGCAG | 1 | 0 | 0 | 0 | 12 |  | H25_CTC_TTCTAGTAGCTACTAC | 0 | 0 | 0 | 0 | 13 |
| H42_CTC_CCTATGTTCACACCTT | 0 | 1 | 0 | 0 | 9 |  | H25_CTC_TTCTAGTGTCGGTGTC | 0 | 0 | 0 | 0 | 28 |
| H42_CTC_CCTATTATCCCAGACG | 0 | 0 | 0 | 0 | 18 |  | H25_CTC_TTCTGTACACTAGGCC | 0 | 0 | 0 | 0 | 0 |
| H42_CTC_CCTCACGCATCACGGA | 0 | 1 | 0 | 0 | 2 |  | H25_CTC_TTCTTCCTCGCTTTAT | 0 | 0 | 0 | 0 | 2 |
| H42_CTC_CCTCGATCATTGCCGT | 0 | 1 | 0 | 0 | 1 |  | H25_CTC_TTCTTGAGTGGGATTG | 0 | 0 | 0 | 0 | 99 |
| H42_CTC_CCTGAGACAACTAATG | 0 | 1 | 0 | 0 | 4 |  | H25_CTC_TTGATGGGTATGTGTC | 0 | 0 | 0 | 0 | 53 |
| H42_CTC_CCTGGCAAGTACCAGC | 0 | 1 | 0 | 0 | 32 |  | H25_CTC_TTGATGGGTGGGAGAG | 0 | 0 | 0 | 0 | 104 |
| H42_CTC_CCTGGTTCACCTTAAT | 1 | 0 | 0 | 0 | 8 |  | H25_CTC_TTGATGGTCTCCATAT | 0 | 0 | 0 | 0 | 0 |
| H42_CTC_CCTTAATCAACTGCCT | 0 | 0 | 0 | 0 | 7 |  | H25_CTC_TTGCCTGCATCCTCAC | 0 | 0 | 0 | 12 | 79 |
| H42_CTC_CCTTCGATCTCACACT | 0 | 0 | 0 | 0 | 38 |  | H25_CTC_TTGGTTTAGAATTTGG | 0 | 0 | 0 | 0 | 1 |
| H42_CTC_CGAATTTAGTAGCACC | 0 | 0 | 0 | 0 | 18 |  | H25_CTC_TTGTGTTGTAGATTAG | 0 | 0 | 0 | 0 | 57 |
| H42_CTC_CGATCACAGCTTCCGC | 1 | 1 | 0 | 0 | 2 |  | H25_CTC_TTTATGCAGCGTCTCG | 0 | 0 | 0 | 6 | 31 |
| H42_CTC_CGATCAGCAATCGAGT | 0 | 0 | 0 | 0 | 5 |  | H25_CTC_TTTCACAAGCATGTTC | 0 | 0 | 0 | 7 | 130 |
| H42_CTC_CGATCAGCACCTGCTG | 0 | 0 | 1 | 5 | 43 |  | H25_CTC_TTTCACATCTGCGTCT | 0 | 0 | 0 | 99 | 64 |
| H42_CTC_CGCACAACAAGTGTCG | 2 | 0 | 0 | 0 | 7 |  | H25_CTC_TTTGACTAGTGATAGT | 0 | 0 | 0 | 0 | 21 |
| H42_CTC_CGCACAACATTACATC | 0 | 0 | 0 | 0 | 26 |  | H25_CTC_TTTGGAGGTTGGTAGG | 0 | 0 | 0 | 16 | 29 |
| H42_CTC_CGCAGATTCAATGGTT | 0 | 0 | 0 | 0 | 15 |  | H25_CTC_TTTGTTGCATTGAGGG | 0 | 0 | 0 | 0 | 3 |
| H42_CTC_CGCCCAAAGGTTGGGA | 1 | 0 | 0 | 0 | 44 |  | H26_CTC_AACTCCCGTCAAACTC | 0 | 0 | 0 | 0 | 0 |
| H42_CTC_CGCTATGGTTGACTGT | 0 | 0 | 0 | 1 | 25 |  | H26_CTC_AACTTTCTCACAACGT | 0 | 0 | 0 | 0 | 1 |
| H42_CTC_CGGCCAATCGTACCGG | 1 | 0 | 0 | 0 | 147 |  | H26_CTC_ACATGGTTCTGTTGAG | 0 | 0 | 0 | 0 | 0 |
| H42_CTC_CGGCTAGTCCCGCGAA | 0 | 0 | 0 | 0 | 17 |  | H26_CTC_ACCAGTACATTCTTAC | 0 | 0 | 0 | 0 | 0 |
| H42_CTC_CGGCTTGAGGGCTTCG | 0 | 1 | 0 | 0 | 17 |  | H26_CTC_ACGAGGAGTCACAAGG | 0 | 0 | 0 | 0 | 0 |
| H42_CTC_CGGGACTCAGGCATTG | 0 | 0 | 0 | 0 | 16 |  | H26_CTC_ACTGAGTAGCCTATGT | 0 | 0 | 0 | 0 | 1 |
| H42_CTC_CGGGTTTAGGGGCATA | 1 | 2 | 0 | 0 | 11 |  | H26_CTC_AGACGTTAGCTAGTGG | 0 | 0 | 0 | 0 | 0 |
| H42_CTC_CGGTGTTAGTAGGAAC | 0 | 0 | 0 | 0 | 15 |  | H26_CTC_AGCAGCCCAGGCAGTA | 0 | 0 | 0 | 0 | 0 |
| H42_CTC_CGTAACATCGACCATA | 0 | 0 | 0 | 0 | 38 |  | H26_CTC_AGGGATGTCGTTTAGG | 0 | 0 | 0 | 0 | 0 |
| H42_CTC_CGTACGTAGCCTATCT | 1 | 0 | 0 | 0 | 4 |  | H26_CTC_AGGGTGAAGGATCGCA | 0 | 0 | 0 | 0 | 1 |
| H42_CTC_CGTCAAATCCATAGTG | 0 | 0 | 0 | 1 | 14 |  | H26_CTC_AGTGGGAAGTGTCTCA | 0 | 0 | 0 | 1 | 0 |
| H42_CTC_CGTCACCTCCTTCCTC | 1 | 1 | 0 | 0 | 7 |  | H26_CTC_ATTACTCAGAGTGAGA | 0 | 0 | 0 | 0 | 0 |
| H42_CTC_CGTCATCCAAGTCGCA | 1 | 0 | 0 | 0 | 10 |  | H26_CTC_ATTACTCGTATCGCAT | 0 | 0 | 0 | 0 | 1 |
| H42_CTC_CGTCCATCACCTGCCA | 0 | 1 | 0 | 0 | 17 |  | H26_CTC_ATTATCCCACTGTCGG | 0 | 0 | 0 | 0 | 0 |
| H42_CTC_CGTCCTTGTCAGGCGA | 0 | 0 | 0 | 0 | 27 |  | H26_CTC_ATTGGTGAGAGGTTGC | 0 | 0 | 0 | 0 | 0 |
| H42_CTC_CGTGAAAGTCTCACTC | 1 | 1 | 0 | 0 | 19 |  | H26_CTC_CACAGTATCGGCTTGG | 0 | 0 | 0 | 0 | 0 |
| H42_CTC_CGTGGGTCATTAGCAA | 0 | 1 | 0 | 0 | 9 |  | H26_CTC_CACCTTGCACCCATGG | 0 | 0 | 0 | 0 | 0 |
| H42_CTC_CGTTTAAGTGGACTAA | 1 | 0 | 0 | 0 | 31 |  | H26_CTC_CATGCCTAGTACTTGC | 0 | 0 | 0 | 0 | 0 |
| H42_CTC_CTAACCAAGGGTATGG | 0 | 0 | 0 | 0 | 17 |  | H26_CTC_CCACGGAAGAAACCAT | 0 | 0 | 0 | 0 | 1 |
| H42_CTC_CTACAGGCATCCATTG | 0 | 0 | 0 | 0 | 8 |  | H26_CTC_CCGGTAGGTGGTTTCA | 0 | 0 | 0 | 0 | 0 |
| H42_CTC_CTACAGGCATCCCATA | 0 | 1 | 0 | 0 | 10 |  | H26_CTC_CCTTACGCAGGAATGC | 0 | 0 | 0 | 0 | 1 |
| H42_CTC_CTACCCGCAAGGGAAC | 0 | 1 | 0 | 0 | 17 |  | H26_CTC_CGAGAAGGTTAGTGGG | 0 | 0 | 0 | 0 | 0 |
| H42_CTC_CTACGACAGAATCACT | 0 | 0 | 0 | 0 | 4 |  | H26_CTC_CGCTATCCAAGGGTCA | 0 | 0 | 0 | 0 | 1 |
| H42_CTC_CTACGCCTCAGGTTGC | 0 | 1 | 0 | 0 | 15 |  | H26_CTC_CGTTAGAGTTGAGGTG | 0 | 0 | 0 | 0 | 0 |
| H42_CTC_CTAGAAGGTTTCGATA | 0 | 1 | 0 | 0 | 43 |  | H26_CTC_CTGATAGCAATGTTGC | 0 | 0 | 0 | 0 | 0 |
| H42_CTC_CTAGTATAGCCCGTAG | 0 | 0 | 0 | 1 | 15 |  | H26_CTC_CTGATCCAGATGTTAG | 0 | 0 | 0 | 0 | 1 |
| H42_CTC_CTATATGGTCATCGTG | 1 | 0 | 0 | 1 | 15 |  | H26_CTC_CTGATCCTCGTAGATC | 0 | 0 | 0 | 0 | 1 |
| H42_CTC_CTATCGTTCTGTGAGC | 1 | 0 | 0 | 1 | 12 |  | H26_CTC_GAATAAGAGCGTAATA | 0 | 0 | 0 | 0 | 1 |
| H42_CTC_CTATGCAAGGCATCGT | 0 | 2 | 0 | 0 | 10 |  | H26_CTC_GAATAAGCACTGAAGG | 0 | 0 | 0 | 2 | 0 |
| H42_CTC_CTATGGGCATAACGCG | 0 | 1 | 0 | 0 | 28 |  | H26_CTC_GACGTGCGTCATGCAT | 0 | 0 | 0 | 0 | 1 |
| H42_CTC_CTCAAAGTCACACCTT | 1 | 0 | 0 | 0 | 10 |  | H26_CTC_GACGTTAAGTCACGCC | 0 | 0 | 0 | 0 | 1 |
| H42_CTC_CTCAAAGTCTGGTAAT | 1 | 0 | 0 | 1 | 71 |  | H26_CTC_GATCAGTGTACTTGAC | 0 | 0 | 0 | 0 | 0 |
| H42_CTC_CTCAATTCACTTGACC | 1 | 0 | 0 | 4 | 38 |  | H26_CTC_GCAAACTGTAATCACC | 0 | 0 | 0 | 0 | 0 |
| H42_CTC_CTCAGCTCAGGCTAAT | 0 | 1 | 0 | 1 | 32 |  | H26_CTC_GCAAACTTCATCGCTC | 0 | 0 | 0 | 0 | 1 |
| H42_CTC_CTCAGTAAGCATTGAT | 0 | 1 | 0 | 0 | 29 |  | H26_CTC_GCAATCATCACCACCT | 0 | 0 | 0 | 0 | 0 |
| H42_CTC_CTCATCACACCTCACG | 0 | 3 | 0 | 0 | 44 |  | H26_CTC_GCACATACAGACACTT | 0 | 0 | 0 | 0 | 0 |
| H42_CTC_CTCATCACAGGAAGTA | 0 | 3 | 0 | 0 | 9 |  | H26_CTC_GCGACCAGTAACGTTC | 0 | 0 | 0 | 0 | 0 |
| H42_CTC_CTCATCACAGGTAGTT | 1 | 0 | 0 | 0 | 11 |  | H26_CTC_GCTGCGAAGCAGGTCA | 0 | 0 | 0 | 0 | 1 |
| H42_CTC_CTCATTATCGATCACA | 1 | 0 | 0 | 1 | 7 |  | H26_CTC_GCTGCTTCAATGACCT | 0 | 0 | 0 | 0 | 0 |
| H42_CTC_CTCCAAGGTAATCGGA | 0 | 1 | 0 | 0 | 12 |  | H26_CTC_GGCGACTCAATTCCTT | 0 | 0 | 0 | 0 | 1 |
| H42_CTC_CTCCAATTCTAGCTTC | 1 | 0 | 0 | 0 | 9 |  | H26_CTC_TAAGCGTGTGATGATA | 0 | 0 | 0 | 0 | 0 |
| H42_CTC_CTCCATTAGGAACCGA | 0 | 1 | 0 | 0 | 13 |  | H26_CTC_TAAGCGTTCGCGCCAA | 0 | 0 | 0 | 0 | 1 |
| H42_CTC_CTCCTAGTCACCATAG | 1 | 0 | 0 | 0 | 40 |  | H26_CTC_TACGGTAGTTGATTCG | 0 | 0 | 0 | 0 | 0 |
| H42_CTC_CTCCTAGTCGAATGAA | 1 | 1 | 0 | 0 | 16 |  | H26_CTC_TACTCATCAGTAAGCG | 0 | 0 | 0 | 0 | 1 |
| H42_CTC_CTCCTTGAGTCGAGAG | 0 | 0 | 0 | 0 | 32 |  | H26_CTC_TAGGCATTCTGACCTC | 0 | 0 | 0 | 0 | 1 |
| H42_CTC_CTCGCGAAGATTGCAT | 0 | 1 | 0 | 0 | 20 |  | H26_CTC_TAGTGGTTCAGCACAT | 0 | 0 | 0 | 0 | 0 |
| H42_CTC_CTCGCGTTCCCGGCAA | 0 | 0 | 0 | 1 | 64 |  | H26_CTC_TCAGATGTCTACCAGA | 0 | 0 | 0 | 0 | 0 |
| H42_CTC_CTCGCTTGTTCTGGTG | 0 | 1 | 0 | 0 | 20 |  | H26_CTC_TCAGGATTCCTAAGTG | 0 | 0 | 0 | 0 | 0 |
| H42_CTC_CTCGTTGTCCTGTGCC | 0 | 0 | 0 | 0 | 22 |  | H26_CTC_TCTTCGGGTCAATACC | 0 | 0 | 0 | 0 | 0 |
| H42_CTC_CTCTAGCCAATTAGCA | 0 | 1 | 0 | 0 | 15 |  | H26_CTC_TGCCCTATCTTGCATT | 0 | 0 | 0 | 0 | 0 |
| H42_CTC_CTCTGCATCATCGTGA | 0 | 0 | 0 | 0 | 14 |  | H26_CTC_TGGGAAGAGATGCCTT | 0 | 0 | 0 | 0 | 0 |
| H42_CTC_CTCTTAATCGCTTCCA | 1 | 0 | 0 | 2 | 3 |  | H26_CTC_TGGTTAGAGGAGTAGA | 0 | 0 | 0 | 0 | 0 |
| H42_CTC_CTGCATCCAAACCTAA | 0 | 4 | 0 | 0 | 6 |  | H26_CTC_TGTGGTAGTATATGGA | 0 | 0 | 0 | 0 | 0 |
| H42_CTC_CTGCTCTGTTGTCTGG | 0 | 0 | 0 | 1 | 8 |  | H26_CTC_TTCGGTCGTCTCACCT | 0 | 0 | 0 | 0 | 0 |
| H42_CTC_CTGCTTACAATTAGTG | 2 | 0 | 0 | 0 | 12 |  | H26_CTC_TTGACTTCAGTAAGAT | 0 | 0 | 0 | 0 | 1 |
| H42_CTC_CTGGCAACAATTGACA | 0 | 0 | 0 | 0 | 8 |  | H26_CTC_TTGGCAATCAGTTGAC | 0 | 0 | 0 | 0 | 0 |
| H42_CTC_CTGGGCATCATGCAGC | 1 | 0 | 0 | 0 | 26 |  | H26_CTC_TTTACTGTCTATCCTA | 0 | 0 | 0 | 0 | 0 |
| H42_CTC_CTGTGATGTTGCTCAG | 1 | 2 | 0 | 0 | 7 |  | H26_CTC_TTTATGCGTGCAGTAG | 0 | 0 | 0 | 0 | 2 |
| H42_CTC_CTGTTCCGTTCTGGGC | 0 | 0 | 0 | 0 | 9 |  | H26_CTC_TTTGCGCGTACGAAAT | 0 | 0 | 0 | 0 | 0 |
| H42_CTC_CTTAACCTCAGCTGCG | 0 | 0 | 0 | 0 | 3 |  | H26_CTC_TTTGTCACACCCATGG | 0 | 0 | 0 | 0 | 0 |
| H42_CTC_CTTACGCCACAGGCAC | 0 | 0 | 0 | 0 | 33 |  |  |  |  |  |  |  |
| H42_CTC_CTTCGATTCTAAGTCG | 1 | 0 | 0 | 1 | 15 |  |  |  |  |  |  |  |
| H42_CTC_CTTCGCATCTGGAAGG | 0 | 2 | 0 | 0 | 0 |  |  |  |  |  |  |  |
| H42_CTC_CTTCGTTAGTAACGAA | 1 | 0 | 0 | 0 | 6 |  |  |  |  |  |  |  |
| H42_CTC_CTTGCAAAGAAGGATC | 0 | 0 | 0 | 0 | 5 |  |  |  |  |  |  |  |
| H42_CTC_CTTGCAAAGCTACTCT | 0 | 1 | 0 | 0 | 19 |  |  |  |  |  |  |  |
| H42_CTC_CTTGCATTCCATGAGC | 0 | 0 | 0 | 0 | 5 |  |  |  |  |  |  |  |
| H42_CTC_CTTGGCCTCCCGCATC | 1 | 1 | 0 | 0 | 43 |  |  |  |  |  |  |  |
| H42_CTC_GCAATTGTCCCTGGAG | 0 | 1 | 0 | 0 | 5 |  |  |  |  |  |  |  |
| H42_CTC_GCACAATCATGGCTTC | 1 | 1 | 0 | 0 | 17 |  |  |  |  |  |  |  |
| H42_CTC_GCACCGATCCTCACCA | 0 | 1 | 0 | 0 | 12 |  |  |  |  |  |  |  |
| H42_CTC_GCACGAAAGGAGTATA | 0 | 1 | 0 | 0 | 12 |  |  |  |  |  |  |  |
| H42_CTC_GCATAACCATCAATCT | 0 | 0 | 0 | 0 | 46 |  |  |  |  |  |  |  |
| H42_CTC_GCATTATAGGTTCCGG | 0 | 1 | 0 | 0 | 19 |  |  |  |  |  |  |  |
| H42_CTC_GCCAAATCAGTTGCTC | 0 | 1 | 0 | 1 | 4 |  |  |  |  |  |  |  |
| H42_CTC_GCCACATAGCCGCAAT | 0 | 0 | 0 | 0 | 3 |  |  |  |  |  |  |  |
| H42_CTC_GCCACTTCATGCAGAT | 0 | 1 | 0 | 0 | 29 |  |  |  |  |  |  |  |
| H42_CTC_GCCATCAGTTGCGTAT | 1 | 0 | 0 | 0 | 5 |  |  |  |  |  |  |  |
| H42_CTC_GCCCGTAAGCAATCGC | 0 | 2 | 0 | 0 | 11 |  |  |  |  |  |  |  |
| H42_CTC_GCCTGCAAGTCGCGCA | 1 | 0 | 0 | 1 | 25 |  |  |  |  |  |  |  |
| H42_CTC_GCCTGTTCAAGTGCGC | 1 | 0 | 0 | 0 | 6 |  |  |  |  |  |  |  |
| H42_CTC_GCGATAAGTGATGCGG | 0 | 1 | 0 | 0 | 34 |  |  |  |  |  |  |  |
| H42_CTC_GCGCTAGTCCCTCACA | 0 | 1 | 0 | 2 | 45 |  |  |  |  |  |  |  |
| H42_CTC_GCGGAGTAGGTAATCT | 0 | 0 | 0 | 1 | 5 |  |  |  |  |  |  |  |
| H42_CTC_GCGGATGGTAGTGTCT | 0 | 1 | 0 | 0 | 4 |  |  |  |  |  |  |  |
| H42_CTC_GCGGTAAAGGTAGGAT | 1 | 0 | 0 | 0 | 4 |  |  |  |  |  |  |  |
| H42_CTC_GCGTAATCATGAGCCA | 0 | 0 | 0 | 0 | 12 |  |  |  |  |  |  |  |
| H42_CTC_GCGTCAACACTTCATG | 0 | 1 | 0 | 0 | 16 |  |  |  |  |  |  |  |
| H42_CTC_GCGTTAATCCGGAACT | 0 | 1 | 0 | 4 | 12 |  |  |  |  |  |  |  |
| H42_CTC_GCGTTATGTTAGACCT | 0 | 0 | 0 | 0 | 19 |  |  |  |  |  |  |  |
| H42_CTC_GCGTTTCCATGATAGC | 1 | 0 | 0 | 6 | 23 |  |  |  |  |  |  |  |
| H42_CTC_GCTCACCTCTATGTCC | 0 | 0 | 0 | 1 | 39 |  |  |  |  |  |  |  |
| H42_CTC_GCTCATAAGTGCGGAA | 0 | 0 | 0 | 0 | 13 |  |  |  |  |  |  |  |
| H42_CTC_GCTCCATCAATATACG | 0 | 0 | 0 | 1 | 18 |  |  |  |  |  |  |  |
| H42_CTC_GCTGCCTTCCTGCCTA | 0 | 1 | 0 | 0 | 15 |  |  |  |  |  |  |  |
| H42_CTC_GCTTGCCTCTAATGGT | 1 | 0 | 0 | 0 | 22 |  |  |  |  |  |  |  |
| H42_CTC_GGAAATGCAAGGGCTC | 0 | 0 | 0 | 0 | 12 |  |  |  |  |  |  |  |
| H42_CTC_GGAAGGTCAATCAATG | 2 | 0 | 0 | 0 | 2 |  |  |  |  |  |  |  |
| H42_CTC_GGACATAGTGCAACGG | 0 | 1 | 0 | 8 | 28 |  |  |  |  |  |  |  |
| H42_CTC_GGACGATGTTTCAGTA | 0 | 0 | 0 | 0 | 9 |  |  |  |  |  |  |  |
| H42_CTC_GGAGCATGTTCGATAC | 0 | 2 | 0 | 0 | 5 |  |  |  |  |  |  |  |
| H42_CTC_GGAGTAACATTGGGCA | 0 | 0 | 0 | 1 | 15 |  |  |  |  |  |  |  |
| H42_CTC_GGAGTCAAGCCGATTA | 1 | 0 | 0 | 0 | 55 |  |  |  |  |  |  |  |
| H42_CTC_GGATCGCCAAGTGAAG | 1 | 0 | 0 | 0 | 13 |  |  |  |  |  |  |  |
| H42_CTC_GGCGGTAGTGATAGAT | 0 | 0 | 0 | 0 | 6 |  |  |  |  |  |  |  |

### Table S4. Clinical characteristics of a prospective cohort of patients for CTC isolation and characterization.

| **Patient** | **Dataset** | **Age** | **Gender** | **Stage** | **Type of cancer** | **Distant Metastasis** | **Metastatic sites** | **Cancer subtype** |
| --- | --- | --- | --- | --- | --- | --- | --- | --- |
| P1 | Inhouse | 52 | male | T2N1M0 | lung cancer | no | No | Adenocarcinoma |
| P2 | Inhouse | 49 | female | T3N1M0 | lung cancer | no | No | squamous cell carcinoma |
| P3 | Inhouse | 45 | male | T3N2M0 | lung cancer | no | No | squamous cell carcinoma |
| P4 | Inhouse | 54 | male | T4bN1M1 | lung cancer | yes | Bone | squamous cell carcinoma |
| P5 | Inhouse | 69 | female | T4bN0M1 | lung cancer | yes | Bone | Adenocarcinoma |
| P6 | Inhouse | 39 | female | T4bN3M1 | lung cancer | yes | Bone,Liver | squamous cell carcinoma |
| P7 | Inhouse | 58 | male | / | lung cancer | yes | brain | Adenocarcinoma |
| P8 | Inhouse | 61 | male | T2NXM1 | lung cancer | yes | brian | Adenocarcinoma |
| P9 | Inhouse | 73 | male | / | lung cancer | yes | Mediastinal lymph node | Smal cell |
| P10 | Inhouse | 61 | male | T3N1M0 | melanoma | no | No | Mucosal melanoma of head and neck |
| P11 | Inhouse | 51 | female | T4N2M0 | melanoma | no | No | Mucosal melanoma of head and neck |
| P12 | Inhouse | 51 | female | T3N2M0 | melanoma | no | No | Mucosal melanoma of head and neck |
| P13 | Inhouse | 67 | female | T4N3M1 | melanoma | yes | Bone,Lung | Mucosal melanoma of head and neck |
| P14 | Inhouse | 56 | male | T4N1M1 | melanoma | yes | Lung,Pleura | Mucosal melanoma of head and neck |
| P15 | Inhouse | 60 | female | T1aN0M0 | melanoma | yes | No | acral malignant melanoma |
| P16 | Inhouse | 40 | male | TxN2cM1d | melanoma | yes | Brain,Left inguinal lymph node | acral malignant melanoma |
| P17 | Inhouse | 63 | male | T3bN3cM1d | melanoma | yes | Brain,Lung,Right inguinal lymph node,Right popliteal lymph node | acral malignant melanoma |
| P18 | Inhouse | 62 | male | T4bN1M1 | melanoma | yes | Brain,Lung,Bone | Mucosal melanoma of head and neck |

### Table S5. List of siRNA sequences.

| Name | 5'-3' |
| --- | --- |
| CBX3 si1 | GCAAATATGAAGTGTCCTCAA |
| CBX3 si2 | CGACGTGTAGTGAATGGGAAA |
| EP300 si1 | CAAUUCCGAGACAUCUUGAGATT |
| EP300 si2 | GCCUUCACAAUUCCGAGACAUTT |

### Table S6. List of antibody catalog numbers.

| pan Cytokeratin | abcam | ab215838 | RRID:AB_2922672 |
| --- | --- | --- | --- |
| NG2 | abcam | ab275024 | RRID:AB_2922401 |
| CBX3 | abcam | ab217999 | RRID:AB_3697705 |
| GPX4 | abcam | ab125066 | RRID:AB_10973901 |
| EPCAM | CST | #14452 | RRID:AB_2736866 |
| EP300 | CST | #54062 | RRID:AB_2799450 |

### Table S7. List of qRT-PCR primer sequences.

|  | 5'-3' |  |
| --- | --- | --- |
| GPX4-CHIP | forward primer | AGTCCTGACTACGGCCTCCATACCCGCCCGTCTCCC |
|  | reverse primer | CAGTCCTGACTACGGCCTCCCATACCCGCCCGTCTCCGCTGGACGAGGGGAGGAG |
| GPX4 | forward primer | GAGGCAAGACCGAAGTAAACTAC |
|  | reverse primer | CCGAACTGGTTACACGGGAA |
| CBX3 | forward primer | TAGATCGACGTGTAGTGAATGGG |
|  | reverse primer | TGTCTGTGGCACCAATTATTCTT |
| PCBP2 | forward primer | ACTCTCACCATCCGGCTACTT |
|  | reverse primer | TCGCGCATCTTCTTAACTGATTC |
| NFE2L2 | forward primer | TCAGCGACGGAAAGAGTATGA |
|  | reverse primer | CCACTGGTTTCTGACTGGATGT |
| G6PD | forward primer | CGAGGCCGTCACCAAGAAC |
|  | reverse primer | GTAGTGGTCGATGCGGTAGA |
