# Supplementary material for: CBX3 confers ferroptosis resistance during blood-borne metastasis
Source: J Hematol Oncol. 2026 Jan 15;19:9. doi: 10.1186/s13045-025-01777-0 (PMC12809899; doi:10.1186/s13045-025-01777-0)
Supplement: Supplementary file 3 — Supplementary Material 3 [file 13045_2025_1777_MOESM3_ESM.docx]

**CBX3 confers** **f****erroptosis resistance during blood-borne metastasis**

Chun Wu^1,2, #^, Xuefei Liu^1, 3, #^, Boxi Zhao^1, #^, Mao Zhao^4^, Binyu Zhang^1,5^, Guanyin Huang^1^, Yixin Cheng^2^, Shuqian Zheng^1^, Jianyang Hu^1^, Ling Guo^2^, Weinan Guo^4,^*, Jun Tan^6,7^*, Xin Hong^1,8,9, 10,^ *

**Content**

[Supplementary Methods 3](#_Toc215659210)

[**Clinical samples collection** 3](#_Toc215659211)

[**Cell lines and Cell culture** 3](#_Toc215659212)

[**CTCs isolation** 3](#_Toc215659213)

[**Small interfering (si) RNA and Plasmid transfection** 4](#_Toc215659214)

[**Construction of stable cell lines** 4](#_Toc215659215)

[**Western blot analysis** 4](#_Toc215659216)

[**Quantitative real-time PCR** 5](#_Toc215659217)

[**Cell viability, migration and invasion assay** 5](#_Toc215659218)

[**Multiplex Immunohistochemistry (mIHC)** 5](#_Toc215659219)

[**Immunofluorescence staining and confocal microscopy** 6](#_Toc215659220)

[**Chromatin Immunoprecipitation–qPCR analysis** 6](#_Toc215659221)

[**Cell viability assay** 7](#_Toc215659222)

[**Annexin V/PI double staining and FACS analysis** 7](#_Toc215659223)

[**Lipid peroxidation assessed by BODIPY-C11** 8](#_Toc215659224)

[**Mice** 8](#_Toc215659225)

[**Sample preparation and scRNA sequencing** 8](#_Toc215659226)

[CITE-Seq of antibody-derived tags (ADT) Staining Protocol 9](#_Toc215659227)

[**Single-cell RNA sequencing data analysis** 9](#_Toc215659228)

[**Differential expression analysis between cell types** 10](#_Toc215659229)

[**Copy number variation (CNV) calling from single cell RNA sequencing** 10](#_Toc215659230)

[**Genomic instability estimation** 10](#_Toc215659231)

[**Pathway enrichment analysis** 11](#_Toc215659232)

[**Whole exome sequencing (WES) data processing** 11](#_Toc215659233)

[**Chip-seq and data analysis** 11](#_Toc215659234)

[**Transcription factor module analysis** 11](#_Toc215659235)

[**Single-cell mutation analysis** 11](#_Toc215659236)

[**Public datasets used in this study** 12](#_Toc215659237)

[Statistical analysis 12](#_Toc215659238)

[References 13](#_Toc215659239)

**Supplementary Methods**

**Clinical samples collection**

Fresh LUAD-BrM specimens, along with tumor tissues and fresh blood samples, were procured and consented from the Central South University Xiangya Hospital under item number 202407141. 4 patients diagnosed with LUAD-BrM provided written informed consent for participation in the study. The collection and utilization of these clinical specimens were sanctioned by the Central South University Xiangya Hospital ethical review board. The clinical information of each patient is as follows:

Patient H25 (Case1):

A 58-year-old male patient presented with right-sided motor dysfunction and visual field impairment. Preoperative neuroimaging revealed a left parieto-occipital lesion measuring 19×17 mm. A chest CT scan identified a 9×7 mm nodule in the apicoposterior segment of the left upper lobe. Histopathological evaluation confirmed a poorly differentiated adenocarcinoma of pulmonary origin.

Patient H26 (Case2):

A 35-year-old male patient was initially diagnosed in 2015 with synchronous lesions located in the right frontal lobe, right occipital lobe, and lung apex. Surgical excision of the larger frontal lesion was performed, and histopathological analysis confirmed the presence of lung adenocarcinoma. In 2018, recurrence of the lesion in the right frontal lobe and progression of the lesion in the right occipital lobe were noted. Subsequent surgical resection once again identified adenocarcinoma of pulmonary origin. By 2024, the patient experienced a recurrence in the right occipital lobe, with pathological findings indicating a moderately differentiated lung adenocarcinoma.

Patient H41 (Case3):

A 67-year-old male patient was admitted with symptoms of headache and an unsteady gait. MRI examination revealed a cystic-solid mass in the left cerebellum, measuring 45 × 35 mm. A chest CT scan identified a 44×33 mm mass involving the left hilar region and the left upper lobe. Pathological examination of the resected intracranial lesion indicated a poorly differentiated squamous cell carcinoma of pulmonary origin.

Patient H42 (Case4):

A 53-year-old female patient presented with progressively worsening weakness in the right-sided limbs. Preoperative magnetic resonance imaging (MRI) identified a lesion in the left frontoparietal region, measuring approximately 28×24 mm, accompanied by significant perilesional edema. A chest computed tomography (CT) scan revealed a 12 ×10 mm nodule located in the right middle lobe. Surgical resection of the intracranial lesion was performed, and histopathological analysis confirmed the presence of a moderately to poorly differentiated adenocarcinoma, consistent with a primary pulmonary origin.

**Cell lines and Cell culture**

The H1975 human lung cancer cell line and A375 melanoma cell line, obtained from the Sun Yat-sen University Cancer Center on September 2, 2022, underwent similar STR verification and contamination screening. These cell lines were cultured in DMEM medium (Gibco, Canada) containing 10% fetal bovine serum (Sigma, USA) and incubated at 37°C under 5% CO₂. Indirect coculture experiments utilized 0.4 μm transwell inserts (Corning, NY, USA).

**CTCs isolation**

Circulating tumor cells (CTCs) were isolated from peripheral blood samples of lung cancer and melanoma patients using a four-stage spiral microfluidic device (Celutriate Chip 1) based on inertial microfluidics[1]. This label-free enrichment system separates CTCs from whole blood by exploiting size-dependent inertial focusing and Dean flow effects, effectively depleting hematopoietic cells while retaining larger target cells. Fresh peripheral blood (20 mL) was injected directly into the microfluidic device without pretreatment, reducing sample handling and maintaining cell viability. The sample passed sequentially through four spiral microchannel stages incorporating lateral displacement geometry, where hydrodynamic forces directed cells according to size. This design efficiently separated larger CTCs from smaller blood components, including erythrocytes and leukocytes. The complete enrichment process required only 12 minutes, demonstrating both rapid processing and high throughput. Reported results showed CTC recovery efficiencies of 52.3-65.8% alongside >99.95% leukocyte depletion, substantially enhancing the purity of isolated CTC populations.

**Small interfering (si) RNA and Plasmid transfection**

CBX3 and negative control (NC) siRNAs were synthesized by GenePharma (China), and their sequences (5' to 3') are provided in **Supplementary Table 5**. The CBX3 overexpression plasmid was constructed by Miaoling (China) through insertion of the CBX3 coding sequence into the expression vector. Cells were transiently transfected with either siRNAs or overexpression plasmids using Lipofectamine 3000 (Invitrogen, USA) according to the manufacturer's protocol and were harvested 48 hours post-transfection.

**Construction of stable cell lines**

CBX3 overexpression stable A375 and H1975 cells were established by infecting cells with lentivirus expressing the pLV3-CMV-CBX3(human)-EF1a-Fluc-Puro.

**Western blot analysis**

Cell lysis was performed using RIPA buffer (Beyotime, China) containing 1% protease and phosphatase inhibitors (Thermo Fisher Scientific, USA). Protein extracts were sonicated and quantified using a BCA Protein Assay Kit (Beyotime, China). Equal amounts of protein samples were loaded onto 10% SDS-PAGE gels for electrophoretic separation, followed by electro transfer onto 0.45 μm PVDF membranes (Merck-Millipore, Germany). Membranes were blocked with QuickBlock™ Blocking Buffer (Beyotime, China) for 15 minutes at room temperature, then incubated with primary antibodies overnight at 4°C. Subsequently, membranes were incubated with HRP-conjugated secondary antibodies for 1 hour at room temperature. Immunoreactive bands were detected using an enhanced chemiluminescence detection system (Millipore, MA, USA). Detailed antibody information is presented in **Supplementary Table 6**.

**Quantitative real-time PCR**

Quantitative real-time PCR (qRT-PCR) was employed to measure mRNA expression levels of target genes. Total RNA was extracted using TRIzol reagent (Thermo Fisher, USA) and reverse transcribed into cDNA with a PrimeScriptTM RT Kit (Takara, China). PCR amplification was conducted using the SYBR Green PCR kit (Thermo Fisher Scientific, USA) on a CFX96 Touch Real-Time PCR Detection System (Bio-rad, USA). Target gene expression was normalized to GAPDH and calculated using the 2-ΔΔCT method. The primer sequences used for RT-qPCR are listed in **Supplementary Table 7**.

**Cell viability, migration and invasion assay**

Cell proliferation was evaluated using the Cell Counting Kit-8 (CCK-8; Beyotime, China). Cells were plated at a density of 1000 cells per well in 96-well plates and optical density was measured at 450 nm using a microplate reader (Bio-Rad Laboratories, USA) at designated time intervals. For migration analysis, 8 μm pore transwell inserts were positioned in 24-well plates containing 700μL culture medium with 10% FBS. For invasion assessment, insert membranes were pre-coated with 30% Matrigel (Corning, USA) diluted in DMEM. A total of 5×10^4 serum-deprived cells were seeded into the upper chamber and cultured for 24 hours. Following medium removal, cells were fixed with methanol and stained with crystal violet solution. Microscopic images were acquired using an OLYMPUS microscope, and cell quantification was conducted using ImageJ software.

**Multiplex Immunohistochemistry (mIHC)**

Multiplex immunohistochemistry was conducted using the PANO 7-plex IHC kit (Panovue, Beijing, China). Tissue sections were incubated at 65°C for 2 hours to improve adherence, followed by deparaffinization in xylene and rehydration through descending ethanol concentrations (100%, 95%, 70%, 50%). Sections were fixed in 10% neutral buffered formalin for 30 minutes prior to antigen retrieval using EDTA buffer (pH 9.0, ZSGB-Bio, Beijing, China) with microwave heating. Nonspecific binding was minimized through blocking procedures before sequential application of primary antibodies combined with horseradish peroxidase (HRP)-conjugated secondary antibodies. Tyramide signal amplification (TSA) was employed following each primary antibody incubation to enhance detection sensitivity. Biotinylated rabbit polyclonal anti-rabbit and rabbit anti-mouse secondary antibodies were subsequently applied, followed by HRP-conjugated streptavidin according to the manufacturer's protocol (Panovue, Beijing, China). Chromogenic detection utilized biotinylated secondary antibodies with streptavidin-linked alkaline phosphatase, while streptavidin-conjugated fluorophores (excitation wavelengths: 480, 520, 570, 690, or 780 nm) facilitated immunofluorescence visualization. Multispectral image acquisition was performed using the Vectra Polaris Automated Quantitative Pathology Imaging System (Akoya Biosciences, Delaware, USA).

**Immunofluorescence staining and confocal microscopy**

Cells were fixed using 0.4% paraformaldehyde, permeabilized with 0.5% Triton X-100, and blocked with 1% BSA-PBS solution. Primary antibodies were applied overnight at 4°C for target protein detection. Coverslips were then incubated with HRP-conjugated secondary antibodies for 30 minutes at room temperature, followed by tyramine signal amplification to enhance fluorescence intensity. Antibody stripping between sequential incubations was accomplished using elution buffer. Confocal dishes were sealed with Mounting Medium containing DAPI (abcam, USA) to prevent fluorescence decay, and images were acquired using an OLYMPUS FV1000 (OLYMPUS, Japan) confocal microscope. Comprehensive antibody information for immunofluorescence staining is provided in **Supplementary Table 6.**

**Chromatin Immunoprecipitation–qPCR analysis**

Chromatin immunoprecipitation (ChIP) experiments were performed following the manufacturer's instructions provided with a commercial kit from CST (USA). Briefly, cells were treated with 1% formaldehyde for crosslinking, followed by nuclear extraction and chromatin fragmentation through sonication after cell lysis. The fragmented chromatin was then diluted in an appropriate buffer and distributed for immunoprecipitation procedures. Following the addition of anti-CBX3 antibody, samples were incubated overnight at 4°C under continuous rotation. Antibody-chromatin complexes were subsequently captured using magnetic protein A/G beads. The isolated DNA was analyzed by quantitative real-time PCR using primers detailed in **Supplementary Table 7**.

**Cell viability assay**

Cell viability was assessed using the Cell Counting Kit-8 (CCK-8, Dojindo) assay. A375 and H1975 cells were plated in 96-well plates at a density of 2×10⁴cells per well. Following a 24-hour treatment with Ferrostatin-1(MedChem Express, MCE) or RSL3(MedChem Express, MCE) the next day, cells were incubated with 10 μl of CCK-8 solution (diluted in 100 μl culture medium per well) for 1 hour at 37°C under 5% CO₂ atmosphere. Absorbance measurements were conducted at 450 nm using a FLUOstar Omega microplate reader (BMG Labtech).

**Annexin V/PI double staining and FACS analysis**

Flow cytometry analysis following annexin V-FITC staining was conducted to assess phosphatidylserine exposure, an initial indicator of apoptotic cell death. Following designated treatments, A375 and H1975 cells were harvested, resuspended, and labeled with annexin V-FITC/PI dye solution (Yeasen Biotechnology) as per the supplier's protocol. Cellular analysis was carried out using a FACScan flow cytometer (BD Biosciences, Le Pont de Claix, France) with 488 nm laser excitation, employing a 515 nm band-pass filter for fluorescein signal acquisition and a >600 nm filter for propidium iodide detection. FlowJo software was utilized for data processing (Becton Dickinson Co., Mountain View, CA, USA). Following proper gating of negative and positive control populations, the proportions of annexin V+/PI− and annexin V+/PI+ cellular subsets were quantified and contrasted against untreated reference samples.

**Lipid peroxidation assessed by BODIPY-C11**

A375 or H1975 cells (20,000 cells/well) that had undergone respective treatments were seeded in 24-well plates. For BODIPY-C11 staining, cells were washed twice with PBS, then incubated in 1 mL Hank's Balanced Salt Solution (HBSS, Gibco) containing 5 um BODIPY 581/591 C11(Sigma) for 15 minutes at 37°C in a tissue culture incubator. Following washing, cells were resuspended in 200 μl fresh HBSS and immediately analyzed by flow cytometry or fluorescence inverted microscopy. Both non-oxidized C11 (PE channel) and oxidized C11 (FITC channel) signals were simultaneously monitored. The ratio of mean fluorescence intensity (MFI) of FITC to MFI of PE was calculated for each sample. In other cases, only the oxidized C11 signal was monitored and the MFI of FITC was calculated. Data were normalized to control samples and expressed as relative lipid ROS.

**Mice**

All mouse experiments conducted in this study received approval from the Institutional Animal Care and Use Committee of Sun Yat-Sen University (IACUC No. L102012022003W). NCG nude mice (aged 4–6 weeks, SPF level, 20–22 g) were obtained from the Guangdong Medical Laboratory Animal Center, Guangdong, China. In the tail vein metastasis model, the experiment was categorized into two distinct groups: the control group and the CBX3 overexpression group. A total of 1 × 10^6 A375 cells or FTOOE A375 cells were administered via tail vein injection into NCG mice. Weekly in vivo imaging of lung and liver metastases was conducted post-inoculation utilizing a small animal fluorescence imaging system (IVIS Spectrum). Brain, liver and lung tissues were excised and preserved in 4% phosphate-buffered neutral formalin. Subsequent analysis of lung tissues was carried out using hematoxylin and eosin (HE) staining and multiplex immunohistochemistry (mIHC).

**Sample preparation and scRNA sequencing**

10x Genomics Cell Preparation Guide describes best practices and general protocols for washing, counting and concentrating cells from both abundant and limited cell suspensions (greater than or less than 100000 total cells, respectively) in preparation for use in10x Genomics Single Cell Protocols. Cell viability was measured with Acridine Orange/Propidium Iodide (AO/PI) kit. scRNA-seq libraries were prepared using ChromiumTM Single Cell G Chip and Chromium Single Cell 5’ Library & Gel Bead Kit v2 and sequencing was accomplished on an Illumina NovaSeq6000 System using a paired-end 150 bp.

## CITE-Seq of antibody-derived tags (ADT) Staining Protocol

Collect the cell suspension to be processed and centrifuge at 400×g for 5 minutes. Discard the supernatant, resuspend the cell pellet in 95 μL of PBS containing 0.04% BSA, then add 5 μL of blocking reagent. Incubate at 4°C in the dark for 15 minutes. After incubation, resuspend the cells in 1 mL of PBS containing 0.04% BSA, centrifuge again at 400×g for 5 minutes, and discard the supernatant. Resuspend the pellet in 98 μL of PBS containing 0.04% BSA, then add 1 μL of CD326 antibody and 1 μL of CD68 antibody. Continue incubating at 4°C in the dark for 15 minutes (gently pipette the cell suspension 15-20 times every 5 minutes during incubation to ensure sufficient antibody-cell binding and prevent cell clumping). After incubation, resuspend the cells in 1 mL of PBS containing 0.04% BSA, centrifuge at 400×g for 5 minutes, and discard the supernatant. Resuspend the cell pellet in an appropriate volume of buffer (e.g., PBS containing 0.04% BSA). Following passing quality control (e.g., verification of cell viability and antibody binding efficiency), the cells can be loaded onto the instrument for subsequent detection.

**Single-cell RNA sequencing data analysis**

The Cell Ranger Single-Cell toolkit (v7.2.0) was applied to align reads for LC-BM tissue and PBMC based on the human reference genome GRCh38. Single cell downstream analysis was based on the Seurat R package[2]. Further quality control was applied to cells based on the following thresholds: 1) a count of expressed genes exceeding 150 but not surpassing 6,000; 2) cells containing mitochondrial RNA content lower than 10%. The DoubletFinder R package was used to remove potential doublets[3]. The gene expression data was then processed by normalizing and scaling each sample’s filtered gene expression matrix using the functions, “NormalizeData” and “ScaleData” in the Seurat package. Batch effects across this case and other tissues were harmonized and the gene expression matrices from all samples were integrated using the Harmony R package[4]. Finally, we identified 36,601 genes. We performed principal component analysis (PCA) on the corrected expression matrix using highly variable genes (HVGs) identified by the “FindVariableGenes” function. The most representative principal components were used to determine different cell types with the “FindCluster” function.

**Differential expression analysis between cell types**

To identify differentially expressed genes for each cell subtype, the “FindAllMarkers” functions from the Seurat package were used with default parameters[2]. The expression differences with P < 0.05 and log2(fold change) > 0.3 were considered differentially expressed genes.

**Copy number variation (CNV) calling from single cell RNA sequencing**

We used the inferCNV R package (inferCNV of the Trinity CTAT Project, provided at https://github.com/broadinstitute/inferCNV) to infer the large-scale chromosomal copy number variations of each cell and verify benign cells and malignant cells. The other parameters were set to defaults. We defined each chromosome amplification or deletion as a CNV event, and the sum of all CNV events determined the CNV score for each cell.

**Genomic instability estimation**

To estimate the genomic instability of each malignant cell, we used the genomicInstability R package, which uses the aREA algorithm to quantify the enrichment of sets of contiguous genes (loci-blocks), on the gene expression profiles to estimate the Genomic Instability Score (GIS) for each analyzed cell.

**Pathway enrichment analysis**

We used the GSEA to analyze genes exhibiting logFC >0.3 and p-value <0.05 within the CTC subpopulation[5]. We used irGSEA package to calculate ferroptosis and apoptosis scores for single cell RNA sequencing data[6].

**Whole exome sequencing (WES) data processing**

For four patients, the WES data was aligned by BWA[7] mem software with the genome reference hg38, and then duplicates were removed using sambamba[8] markdup, realign, and recal with GATK[9] RealignerTargetCreator, IndelRealigner and BaseRecalibrator tools. Somatic copy number variants (SCNVs) were called using Control-FREEC v11.1[10].

**Chip-seq and data analysis**

All chip-seq data for CBX3 were downloaded in public database ChIP-Atlas[11]. We used IGV to visualize the genomic regions, and the enhancer regions were divided into nonoverlapping windows of different sizes (the windows with reads ≤ 1 / 20 of window size were filtered out).

**Transcription factor module analysis**

For transcription factor module analysis, we applied the pySCENIC[12] workflow to detect active transcription factor modules in CTCs, using transcription factor motif scores for hg38 human reference genome from the RcisTarget database.

## Single-cell mutation analysis

We utilized the downstream bam file from samtools, and the vcf file obtained from the Mutect2 program in the GATK software to identify somatic mutations in the WES analysis. We used cellsnp-lite[13] software to determine the mutations that have the same genomic positions in each cell, as identified in the WES analysis. The parameters were -minMAF 0.1 and --minCOUNT 20.

**Public datasets used in this study**

We downloaded the available CTCs from previous study GSE109761 and PJNA662599 from the Gene Expression Omnibus database (GEO, https://www.ncbi.nlm.nih.gov/geo/). We also included the public CTCs RNA-seq datasets from CNGBdb database (CNP0000095). We also included the lung cancer single cell RNA-seq datasets from GEO (GSE131907). Transcriptomic data and clinical information of The Cancer Genome Atlas (TCGA) cohort were downloaded from the UCSC Xena data portal (<https://xenabrowser.net>).

**Statistical analysis**

Comparisons between two groups were performed using two-tailed Student’s t-test under the normality assumption. One-way ANOVA with Dunnett’s T3 multiple-comparison test was used to compare several groups. Spearman’s correlation was used to measure the correlation between two continuous variables and r > 0.3 and *P* < 0.05 was considered significant. Log-rank test was used for univariate survival analyses and showed as the Kaplan-Meier plot. All statistical analyses and visualization were performed using R or GraphPad Prism. The lines in the middle of the box plot are median and the upper and lower lines indicate 25th and 75th percentiles. *P* < 0.05 was considered statistically significant. The number of replicates and statistical tests used in figures were shown in corresponding figure legends.

**References**

1. Huang Y, Yu S, Chao S, Wu L, Tao M, Situ B et al. Isolation of circulating fetal trophoblasts by a four-stage inertial microfluidic device for single-cell analysis and noninvasive prenatal testing. Lab Chip 2020; 20: 4342–4348.

2. Satija R, Farrell JA, Gennert D, Schier AF, Regev A. Spatial reconstruction of single-cell gene expression data. Nature biotechnology 2015; 33: 495–502.

3. McGinnis CS, Murrow LM, Gartner ZJ. DoubletFinder: Doublet Detection in Single-Cell RNA Sequencing Data Using Artificial Nearest Neighbors. Cell systems 2019; 8: 329–337.e324.

4. Korsunsky I, Millard N, Fan J, Slowikowski K, Zhang F, Wei K et al. Fast, sensitive and accurate integration of single-cell data with Harmony. Nature methods 2019; 16: 1289–1296.

5. Subramanian A, Tamayo P, Mootha VK, Mukherjee S, Ebert BL, Gillette MA et al. Gene set enrichment analysis: a knowledge-based approach for interpreting genome-wide expression profiles. Proc Natl Acad Sci U S A 2005; 102: 15545–15550.

6. Fan C, Chen F, Chen Y, Huang L, Wang M, Liu Y et al. irGSEA: the integration of single-cell rank-based gene set enrichment analysis. Brief Bioinform 2024; 25.

7. Li H, Durbin R. Fast and accurate short read alignment with Burrows-Wheeler transform. Bioinformatics (Oxford, England) 2009; 25: 1754–1760.

8. Tarasov A, Vilella AJ, Cuppen E, Nijman IJ, Prins P. Sambamba: fast processing of NGS alignment formats. Bioinformatics (Oxford, England) 2015; 31: 2032–2034.

9. McKenna A, Hanna M, Banks E, Sivachenko A, Cibulskis K, Kernytsky A et al. The Genome Analysis Toolkit: a MapReduce framework for analyzing next-generation DNA sequencing data. Genome research 2010; 20: 1297–1303.

10. Boeva V, Popova T, Bleakley K, Chiche P, Cappo J, Schleiermacher G et al. Control-FREEC: a tool for assessing copy number and allelic content using next-generation sequencing data. Bioinformatics (Oxford, England) 2012; 28: 423–425.

11. Zou Z, Ohta T, Oki S. ChIP-Atlas 3.0: a data-mining suite to explore chromosome architecture together with large-scale regulome data. Nucleic Acids Res 2024; 52: W45–w53.

12. Aibar S, Gonzalez-Blas CB, Moerman T, Huynh-Thu VA, Imrichova H, Hulselmans G et al. SCENIC: single-cell regulatory network inference and clustering. Nature methods 2017; 14: 1083–1086.

13. Huang X, Huang Y. Cellsnp-lite: an efficient tool for genotyping single cells. Bioinformatics 2021; 37: 4569–4571.
