# Supplementary material for: CBX3 confers ferroptosis resistance during blood-borne metastasis
Source: J Hematol Oncol. 2026 Jan 15;19:9. doi: 10.1186/s13045-025-01777-0 (PMC12809899; doi:10.1186/s13045-025-01777-0)
Supplement: Supplementary file 4 — Supplementary Material 4 [file 13045_2025_1777_MOESM4_ESM.docx]

**CBX3 confers** **f****erroptosis resistance during blood-borne metastasis**

Chun Wu^1,2, #^, Xuefei Liu^1, 3, #^, Boxi Zhao^1, #^, Mao Zhao^4^, Binyu Zhang^1,5^, Guanyin Huang^1^, Yixin Cheng^2^, Shuqian Zheng^1^, Jianyang Hu^1^, Ling Guo^2^, Weinan Guo^4,^*, Jun Tan^6,7^*, Xin Hong^1,8,9, 10,^ *

^1^ Department of Biochemistry, SUSTech Homeostatic Medicine Institute, School of Medicine, Southern University of Science and Technology, Shenzhen 518055, Guangdong, China.

^2^ Department of Nasopharyngeal Carcinoma, Sun Yat-sen University Cancer Center, State Key Laboratory of Oncology in South China, Guangzhou, China.

^3^ Department of Hematology and Oncology, Shenzhen Children’s Hospital and School of Medicine, Southern University of Science and Technology, Shenzhen, China.

^4^ Department of Dermatology, Xijing Hospital, Fourth Military Medical University, Xi'an, China.

^5^ Department of Physiology & Pathophysiology, School of Basic Medical Sciences, Capital Medical University, Beijing, 100069, China.

^6^ Department of Neurosurgery, Xiangya Hospital, Central South University, Changsha, Hunan, China.

^7^National Clinical Research Center for Geriatric Disorders, Xiangya Hospital, Central South University, Changsha, Hunan, China

^8^ Key University Laboratory of Metabolism and Health of Guangdong, Southern University of Science and Technology, Shenzhen, Guangdong, China.

^9^ Guangdong Provincial Key Laboratory of Cell Microenvironment and Disease Research, Southern University of Science and Technology, Shenzhen, Guangdong, China.

^#^These authors contributed equally to this work

^10^ Lead contact

*Correspondence should be addressed to Xin Hong (hongx@sustech.edu.cn) or Jun Tan (tanjunsea@csu.edu.cn) or Weinan Guo (guown@fmmu.edu.cn)

**Running Title**: CBX3 counteracts ferroptosis in CTCs

**ABSTRACT**

The survival mechanisms of circulating tumor cells (CTCs) remain poorly understood while these rare cell populations transit through the blood stream and colonize distant organs including the brain. Using single-cell RNA-seq of microfluidically-enriched CTCs and patient-matched brain metastatic tumor cells of lung adenocarcinoma (LUAD), we revealed CTC-selective upregulation of core gene signatures associated with ferroptosis. The malignancy of CTCs was confirmed through comparative genomic instability and copy number variation (CNV) analyses of paired CTCs and metastatic tumor cells using both transcriptomic and whole-exome sequencing. Among the transcription factors elevated in CTCs, Chromobox 3 (CBX3) was the top hit, which was tightly correlated with GPX4 expression. Functionally, CBX3 co-operated with EP300 to protect CTCs from ferroptosis by upregulating GPX4 expression. Genetic depletion of CBX3 triggered ferroptosis and substantially reduced tumor cell survival and invasiveness in two independent cancer cell models. Conversely, CBX3 overexpression was sufficient to promote tumor growth, migration, and invasion *in vitro*, and metastatic progression *in vivo*. Clinically, CBX3 expression was significantly correlated with TNM stage and was predictive of both progression-free interval and overall survival in LUAD. In a prospective cohort of LUAD and melanoma patients, CTCs co-expressing CBX3 and GPX4 were selectively elevated in the blood samples of metastatic cases compared to the non-metastatic group, highlighting the clinical association between ferroptosis-resistant CTCs and metastatic progression.

**Key words:** **CTCs; Ferroptosis resistance; CBX3; GPX4; Clinical correlation**

**To the editor,**

Brain metastases (BrM) occur when CTCs colonize the brain, a lethal process that frequently found in lung cancer and melanoma [1, 2]. Ferroptosis, a novel form of cell death driven by lipid peroxidation, is critically involved in cancer metastasis[3]. Chromobox 3 (CBX3), a member of the heterochromatin protein 1 (HP1) family, regulates epigenetics and transcription of cancer-associated genes [4]. Here, we integrated microfluidic isolation of patient CTCs with single-cell omics and uncovered that CBX3 co-operated with EP300 to protect CTCs from ferroptosis by modulating GPX4 expression.

We performed single-cell omic profiling of CTCs and BrM tumors from four LUAD patients (**Figure 1A-B and S1A, Table S1**). CTCs (~0.1%-10% purity) were enriched using a size-based microfluidic separation platform (**Figure S1B**) [5], and subjected to scRNA-seq with BrM tumors, generating 42215 single cells across 16 major types (**Figure 1C-D**). A distinct cluster of 1153 CTCs expressed ZEB2, NRG1, and the epithelial markers (*KRT8*, *KRT18* and *EPCAM*) at low levels (**Figure 1D-E, Table S2-3**) [6]. Antibody-derived tags (ADT) technology [7] was employed to simultaneously analyze EPCAM protein and mRNA in one patient (H41). We confirmed that CTCs specifically upregulated EPCAM expression, but not in contaminating immune cells (**Figure 1F-G and S1C**).

Genomic instability is an intrinsic feature of malignant cells that distinguishes them from normal cells [8]. Genomic instability and inferCNV analyses revealed the highest genomic instability in tumors and CTCs as validated by whole-exome sequencing (WES) (**Figure 1H-J, S1D**). At least one patient-matched somatic mutation was found in 5.46% (63/1153) of CTCs using scRNA-seq analysis (**Figure 1K and S1E**). Consistently, a significant downregulation of epithelial marker mRNAs in CTCs was observed in HCC (**Figure S1F**). Thus, 1153 potential true CTCs were validated in four LUAD patients with BrM.

These CTCs were enriched with Ferroptosis, but not Apoptosis signatures (**Figure 2A-B and S2A**). CTCs exhibited elevated FTL and TFRC, and the “Iron uptake and transport pathway”, suggesting dysregulated iron homeostasis (**Figure S2B-E**). Transcription factor (TF) analysis identified CBX3 showing the most elevated TF activity score in CTCs, correlating with upregulated ferroptosis genes (**Figure 2C-D**). Functionally, CBX3 knockdown (KD) in H1975 and A375 cells increased lipid peroxidation, elevated Fe^2+^/Fe^3+^, reduced GSH/GSSG, and enhanced susceptibility to RSL3-induced death, which was rescued by ferrostatin-1 (**Figure 2E-F, S2F, S3A-D**), without affecting apoptosis (**Figure S3E**). Thus, CBX3 suppressed ferroptosis in CTCs.

The heterogenous CTCs were stratified into CBX3-high and low groups based on the median CBX3 expression. CBX3-high CTCs upregulated anti-ferroptosis genes including GPX4 (**Figure 2G**). GPX4 expression decreased significantly after CBX3 KD (**Figure 2H-I**). CBX3 and GPX4 expressions were positively correlated in several cancers (**Figure 2J**). The binding of CBX3 at the GPX4 promoter was confirmed by ChIP-seq and ChIP-qPCR (**Figure 2K and S4A**). CBX3 was reported to interact with EP300 to regulate gene transcription in glioblastoma [9]. Interestingly, both CBX3 and EP300 were elevated in CTCs (**Figure 2C**). EP300 KD abolished GPX4 induction in CBX3 overexpression (OE) cells, and abrogated the binding of CBX3 to GPX4 promoter, indicating CBX3 co-operated with EP300 to modulate GPX4 expression (**Figure S4B-C**).

Oncogenic pathways like PI3K signaling were enriched in CBX3-high CTCs (**Figure S5A**). CBX3 KD impaired proliferation, clonogenicity, migration, and invasion (**Figure S5B-D**), whereas CBX3 OE enhanced these traits (**Figure S5E-H**). CBX3 OE A375 cells promoted metastases in immunocompromised mice (**Figure 2L-M**). Multiplexed IHC confirmed elevated NG2^+^CBX3^+^GPX4^+^ metastases in lung, brain, heart, and spine of the OE group compared to NC controls (**Figure 2N and S6A**). Thus, CBX3 promoted metastasis. In TCGA-LUAD cohort, CBX3 expression was correlated with overall survival and TNM stages (**Figure S7A-B**). In melanoma CTCs, high CBX3 was associated with disease progression (**Figure S7C**) [10]. Importantly, a prospective analysis of LUAD and melanoma patient cohort demonstrated that metastatic patients harbored significantly more CBX3^+^GPX4^+^ CTCs than non-metastatic cases (**Figure 2O, Table S4**).

In conclusion, we identified CBX3 as a central driver conferring ferroptosis resistance in CTCs. CBX3^+^GPX4^+^ CTCs could serve as noninvasive biomarkers for the precise monitoring of metastatic progression (**Figure 2P**). CBX3 may affect other stress-response pathways [11], and other TFs like SPI1 may regulate CTC biology. While our patient samples are limited, exploration using a larger patient cohort coupled with validations in CTC cell lines would provide deeper mechanistic insights into CTC-mediated metastasis.

**Abbreviations**

***CTCs:*** Circulating Tumor Cells

***LUAD:*** Lung Adenocarcinoma

***HCC:*** Hepatocellular Carcinoma

***BRCA***: Breast Invasive Carcinoma

***CNV:*** Copy Number Variation

***CBX3:*** Chromobox 3

***HP1:*** Heterochromatin Protein 1

***CAF:*** Cancer-associated Fibroblasts

***cDC:*** Conventional Dendritic Cells

***pDC:*** Plasmacytoid Dendritic Cells

***ADT:*** Antibody-derived Tags

***WES:*** Whole-exome Sequencing

***PD:*** Progressive Disease

***RD:*** Responding Disease

**References**

1. Achrol AS, Rennert RC, Anders C, Soffietti R, Ahluwalia MS, Nayak L et al. Brain metastases. Nat Rev Dis Primers 2019; 5: 5.

2. Pantel K, Alix-Panabières C. Liquid biopsy and minimal residual disease - latest advances and implications for cure. Nat Rev Clin Oncol 2019; 16: 409–424.

3. Jiang X, Stockwell BR, Conrad M. Ferroptosis: mechanisms, biology and role in disease. Nat Rev Mol Cell Biol 2021; 22: 266–282.

4. Wahab MA, Del Gaudio N, Gargiulo B, Quagliariello V, Maurea N, Nebbioso A et al. Exploring the Role of CBX3 as a Potential Therapeutic Target in Lung Cancer. Cancers (Basel) 2024; 16.

5. Huang Y, Yu S, Chao S, Wu L, Tao M, Situ B et al. Isolation of circulating fetal trophoblasts by a four-stage inertial microfluidic device for single-cell analysis and noninvasive prenatal testing. Lab Chip 2020; 20: 4342–4348.

6. Würth R, Donato E, Michel LL, Saini M, Becker L, Cheytan T et al. Circulating tumor cell plasticity determines breast cancer therapy resistance via neuregulin 1-HER3 signaling. Nat Cancer 2025; 6: 67–85.

7. Stoeckius M, Hafemeister C, Stephenson W, Houck-Loomis B, Chattopadhyay PK, Swerdlow H et al. Simultaneous epitope and transcriptome measurement in single cells. Nature methods 2017; 14: 865–868.

8. Killcoyne S, Yusuf A, Fitzgerald RC. Genomic instability signals offer diagnostic possibility in early cancer detection. Trends Genet 2021; 37: 966–972.

9. Wang S, Huang T, Wu Q, Yuan H, Wu X, Yuan F et al. Lactate reprograms glioblastoma immunity through CBX3-regulated histone lactylation. J Clin Invest 2024; 134.

10. Hong X, Roh W, Sullivan RJ, Wong KHK, Wittner BS, Guo H et al. The Lipogenic Regulator SREBP2 Induces Transferrin in Circulating Melanoma Cells and Suppresses Ferroptosis. Cancer Discov 2021; 11: 678–695.

11. Liu YF, Li F, Xu CY, Chen Y, Tu WP, Huang C. SETDB1 recruits CBX3 to regulate the SIRT4/PTEN axis, inhibiting autophagy and promoting ischemia-reperfusion-induced kidney injury. Faseb j 2025; 39: e70509.

**Data availability**

scRNA-seq and whole exome sequencing raw data have been deposited in the Genome Sequence Archive in BIG Data Center, Beijing Institute of Genomics, Chinese Academy of Sciences under accession number HRA012575 that are publicly accessible at https://ngdc.cncb.ac.cn/gsa-human/s/L8gE89XL. Other data is provided within the manuscript or supplementary information files.

**Acknowledgements**

We like to thank CHI BIOTECH CO. LTD. for assistance in single cell RNA sequencing.

**Funding**

This study was supported by the NSFC (No. 82573274 to X.H; No. 824B2089 to X.L; No. 82203397 to J.T.), Guangdong provincial funding awards: 2021QN02Y112, 2023A1515010287 (to X.H). Translational research funding from SUSTech SOM-ShangYiJian Joint Laboratory: 20230004056 (to X.H)

**Author information**

Chun Wu, Xuefei Liu and Boxi Zhao contributed equally to this work as co-first authors.

**Authors and Affiliations**

**Department of Biochemistry, SUSTech Homeostatic Medicine Institute, School of Medicine, Southern University of Science and Technology, Shenzhen 518055, Guangdong, China.**

Xuefei Liu, Boxi Zhao, Binyu Zhang, Guanyin Huang, Shuqian Zheng, Jianyang Hu & Xin Hong

**Department of Nasopharyngeal Carcinoma, Sun Yat-sen University Cancer Center, State Key Laboratory of Oncology in South China, Guangzhou, China.**

Chun Wu & Ling Guo

**Department of Hematology and Oncology, Shenzhen Children’s Hospital and School of Medicine, Southern University of Science and Technology, Shenzhen, China.**

Xuefei Liu

**Department of Dermatology, Xijing Hospital, Fourth Military Medical University, Xi'an, China.**

Mao Zhao & Weinan Guo

**Department of Physiology & Pathophysiology, School of Basic Medical Sciences, Capital Medical University, Beijing, 100069, China.**

Binyu Zhang

**Department of Neurosurgery, Xiangya Hospital, Central South University, Changsha, Hunan, China.**

Jun Tan

**National Clinical Research Center for Geriatric Disorders, Xiangya Hospital, Central South University, Changsha, Hunan, China**

Jun Tan

**Key University Laboratory of Metabolism and Health of Guangdong, Southern University of Science and Technology, Shenzhen, Guangdong, China.**

Xin Hong

**Guangdong Provincial Key Laboratory of Cell Microenvironment and Disease Research, Southern University of Science and Technology, Shenzhen, Guangdong, China.**

Xin Hong

**Author contributions**

C.W., X.L. and X.H. conceived and designed the entire project. W.G. and J.T. guided clinical sample preparation and analysis. C.W. and X.L. designed and supervised the research. C.W, and B.Z. prepared all samples for high-throughput sequencing. X.L. and G.H. performed single-cell RNA-seq. C.W., Y.C., B.Z., S.Z. and J.H. performed experiments in vitro. C.W. and J.H. performed experiments in vivo. X.L. and G.H. performed statistical and bioinformatics analysis of high-through sequencing data. Z.M., W.G., L.G. and J.T. were responsible for tissue sample preparation. C.W., X.L., W.G., J.T. and X.H. prepared the manuscript and all authors commented on the manuscript.

**Corresponding author**

Correspondence to Xin Hong (hongx@sustech.edu.cn) or Jun Tan (tanjunsea@csu.edu.cn) or Weinan Guo (guown@fmmu.edu.cn)

**Ethics declarations**

**Ethics approval and consent to participate**

All samples for sequencing were all collected at Central South University Xiangya Hospital under item number and were approved by the Ethics Committee of Central South University Xiangya Hospital (approval number: 202407141). All samples for mIF were collected at Central South University Xiangya Hospital (approval number: 202407141) and Fourth Military Medical University Xijing Hospital (approval number: KY20252289-C-1). All mouse experiments were approved by the Institutional Animal Care and Use Committee of Sun Yat-sen University Cancer Center (approval number: L102012022003W).

**Consent for publication**

Not applicable.

**Competing interests**

The authors declare no competing interests.

**
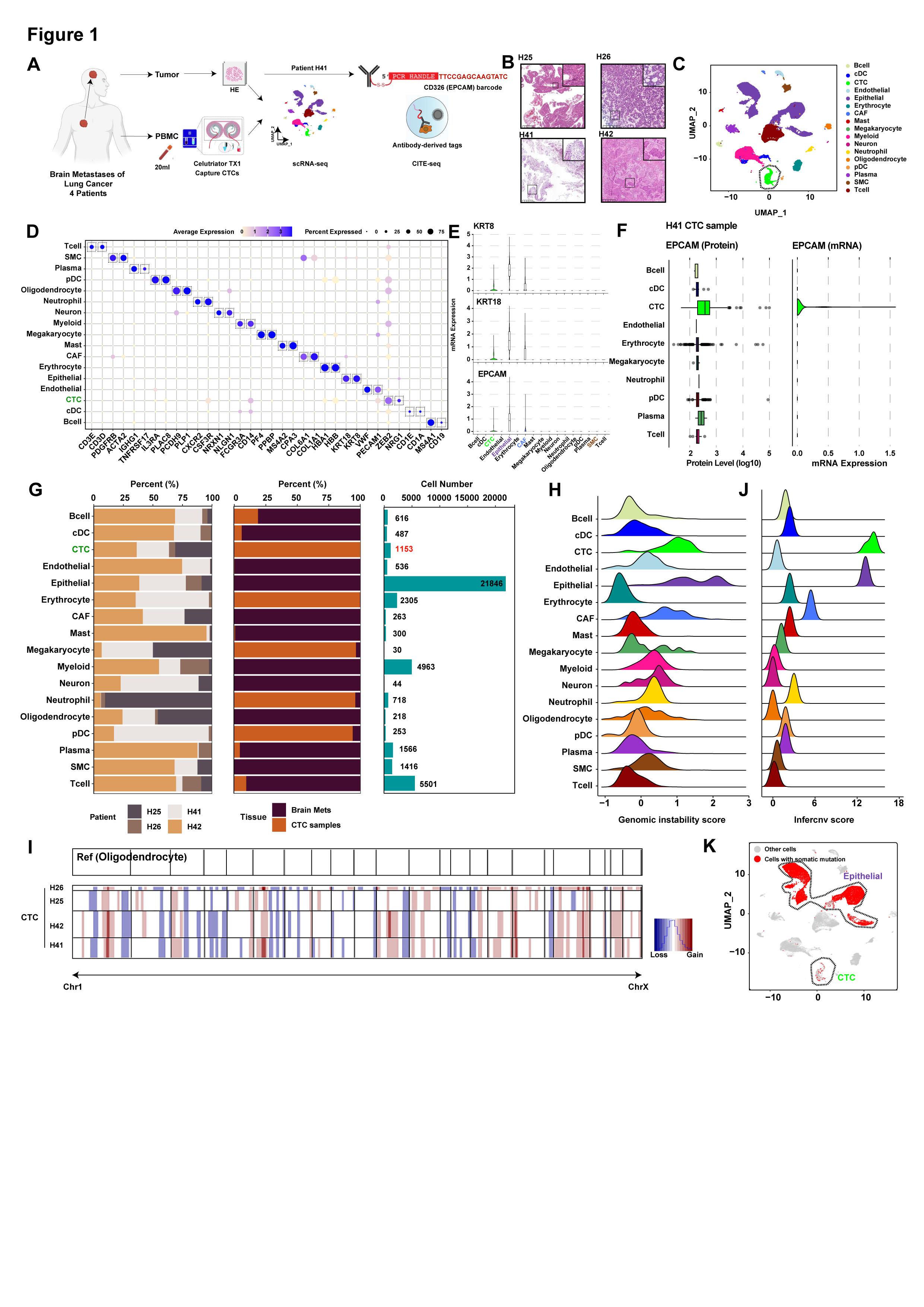
**

**Figure 1. Single-cell omic characterization of CTCs from LUAD patients with BrM.**

**A.** Schematic illustration of sample collection, processing, and single-cell analysis.

**B.** H&E-stained tissue sections showing the morphology of lung cancer brain metastasis.

**C.** UMAP plot showing the subtypes of all cells, each dot indicated a single cell. Color-coded for the cell type.

**D.** Dot plot showing the selected markers for each subtype of all cells. Dot size indicates the fraction of expressing cells and the colors represent normalized gene expression levels.

**E**. Violin plot showing the mRNA expression level of *KRT8*, *KRT18* and *EPCAM* for each subtype of all cells.

**F.** Boxplot showing EPCAM protein levels and mRNA levels across different subtypes of CTCs sample isolated from the whole blood of patient H41.

**G.** Bar plot showing the proportion of the different cell subtypes across patients (left) and tissues (middle). Bar plot showing the cell number of the different cell subtypes (right).

**H.** Ridgeline plot showing the genomic instability score of all cell subtypes. Cells with a genomic instability score >1 (dashed line) are classified as possessing high chromosomal instability, indicative of malignancy.

**I.** Heatmap showing large-scale CNVs for CTCs from four patients, inferring from single-cell RNA-seq analysis. Oligodendrocytes as reference cells. Colors indicate the CNV states. Red: amplifications (Gain); blue: deletions (Loss).

**J.** Ridgeline plot showing inferCNV score of all cell subtypes. The calculation of InferCNV scores is based on Figure 1I: a score of 1 is added for each chromosome harboring CNV, with the final score determined by the copy number alteration status across all chromosomes.

**K.** UMAP showing all cells from Figure 1C. Red highlights CTCs with at least one somatic mutation identified through WES.


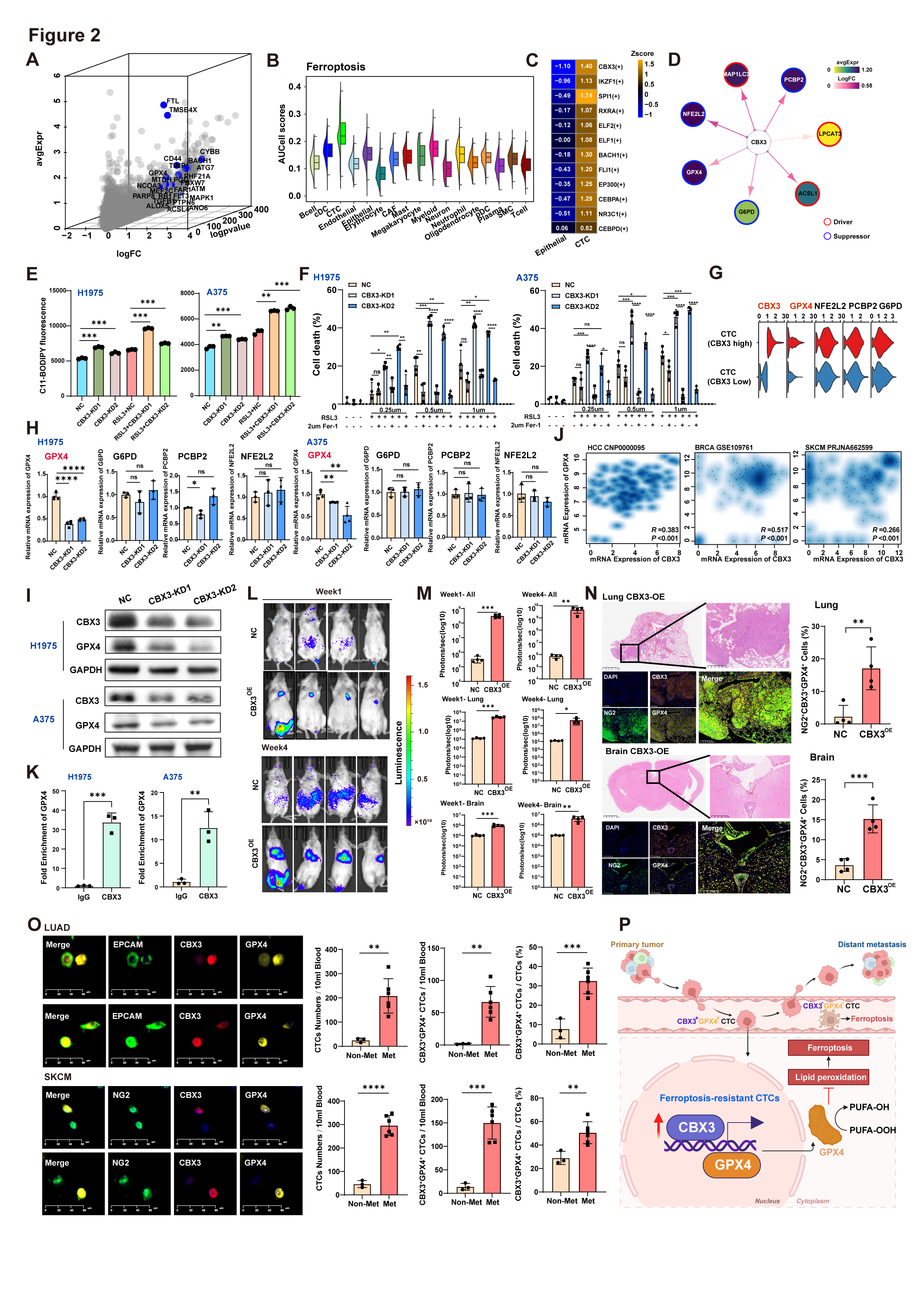


**Figure 2. Experimental and clinical validations of CBX3 in ferroptosis resistance and CTC-mediated metastatic progression.**

**A.** Three-dimensional plot showing genes with elevated expression in CTCs. Blue-highlighted genes are ferroptosis pathway components.

**B.** Box plot showing the ferroptosis scores among the all-cell subtypes. Ferroptosis scores were calculated using AUCell.

**C.** Heatmap showing the activity of regulatory TFs in CTCs calculated by pySCENIC.

**D.** Regulatory network of transcription factor CBX3 and its ferroptosis-associated target genes in CTCs.

**E.** Quantification of lipid peroxidation levels by flow cytometry in H1975 and A375 cells with or without treatment of the ferroptosis inducer RSL3 following CBX3 KD, compared to control cells. Lipid peroxidation is measured using the BODIPY^TM^ 581/591 C11 molecular probe (Therom, USA, cat#D3861). Data are calculated based on three independent biological repeats and statistical significance was assessed by two-tailed Student’s t-test.  **, *P*<0.01 and ***, *P*<0.001.

**F.** Cell viability assay in H1975 and A375 following CBX3 KD, compared with Control cells. RSL3 as a ferroptosis inducer enhances CBX3 KD-increased ferroptosis (0.25 μM, 0.5 μM and 1 μM). The cell viability phenotype is rescued by lipophilic antioxidants, Ferrostatin-1 (2 μM). P values were calculated using a two-tailed Student’s t-test. ns, no significance, *, *P*<0.05, **, *P*<0.01, ***, *P*<0.001 and ****, *P*<0.0001.

**G.** Violin plot showing the differential expression levels of ferroptosis resistance genes in CBX3-high and CBX3-low CTCs. The grouping of CTCs is based on mRNA expression levels of CBX3. CTCs were stratified into "CBX3 high" and "CBX3 low" groups based on the median value of CBX3 expression.

**H.** RT-qPCR analysis showing the mRNA expression levels of *GPX4*, *G6PD*, *PCBP2* and *NFE2L2* in H1975 and A375 cells following CBX3 KD. Data are calculated based on three independent biological repeats and statistical significance was assessed by two-tailed Student’s t-test. ns, no significance, *, *P*<0.05, **, *P*<0.01 and ****, *P*<0.0001.

**I.** Western blot analysis displaying the CBX3 and GPX4 expression in H1975 and A375 following CBX3 KD.

**J.** Scatter plot showing the correlation between CBX3 expression and GPX4 expression in the HCC (liver cancer), BRCA (breast cancer) and SKCM (melanoma) dataset.

**K.** ChIP-PCR assay was performed to detect the extent of CBX3 binding to the GPX4 promoter region in H1975 and A375 cells. P values were calculated using a two-tailed Student’s t-test **, *P*<0.01 and ***, *P*<0.001.

**L.** Tumor metastasis model established by tail vein injection of control A375 cells expressing luciferase and CBX3-OE A375 cells expressing luciferase into immunocompromised NCG mice. The image shows IVIS imaging captured at week 1 and week 4. All images were adjusted to the same radiance scale.

**M.** Bar plot shows the fluorescence values of whole body, lung and brain signal obtained from IVIS imaging captured at the week 1 and week 4 for the two experimental groups (NC and CBX3-OE groups). P values were calculated using a two-tailed Student’s t-test. *, *P*<0.05, **, *P*<0.01 and ***, *P*<0.001.

**N.** H&E staining of lungs and brains dissected from two experimental groups (NC and CBX3-OE) of the metastatic mouse model (Top). mIHC images showing the expression of NG2 (green), GPX4 (yellow) and CBX3 (orange) in xenograft tumors in different groups (Bottom). Barplot showing the fraction of NG2^+^CBX3^+^GPX4^+^ cells among all DAPI+ cells in xenograft tumors in different groups. Y-axis, % of NG2^+^CBX3^+^GPX4^+^ / DAPI^+^ cells. P values were calculated using a two-tailed Student’s t-test. *, *P*<0.05 and ***, *P*<0.001.

**O.** Multi-immunofluorescence (mIF) staining of CTCs showing DAPI (nuclei), EPCAM (epithelial marker) or NG2 (melanoma marker), CBX3, and GPX4 (left). EPCAM^+^ cells were identified as lung CTCs and NG2^+^ cells was identified as melanoma CTCs. Barplot showing the number of CTCs, the number of CBX3^+^GPX4^+^ CTCs and the fraction of CBX3^+^GPX4^+^ CTCs among all CTCs between non-metastasis and metastasis LUAD and SKCM patients. Data is calculated based on three independent non-metastasis patients and six independent metastasis patients were assessed by two-tailed Student’s t-test.  **, *P*<0.01, ***, *P*<0.001 and ****, *P*<0.0001.

**P.** Schematic illustration of the CTCs confers ferroptosis resistance through the CBX3/GPX4 axis to promote CTC survival and metastasis.
